# Systems biology-based analysis exploring shared biomarkers and pathogenesis of myocardial infarction combined with osteoarthritis

**DOI:** 10.3389/fimmu.2024.1398990

**Published:** 2024-07-17

**Authors:** Yuan Luo, Yongrui Liu, Weiqi Xue, Weifeng He, Di Lv, Huanyi Zhao

**Affiliations:** ^1^ The First Clinical Medical College, Guangzhou University of Chinese Medicine, Guangzhou, Guangdong, China; ^2^ Department of Emergency, The First Affiliated Hospital of Guangzhou University of Chinese Medicine, Guangzhou, Guangdong, China; ^3^ Department of Orthopedics, Taizhou Hospital of Traditional Chinese Medicine, Taizhou, Jiangsu, China; ^4^ Department of Cardiovascular Medicine, The First Affiliated Hospital of Guangzhou University of Chinese Medicine, Guangzhou, Guangdong, China

**Keywords:** myocardial infarction, osteoarthritis, systems biology, immune cell infiltration, biomarkers, MAPK signaling pathway

## Abstract

**Background:**

More and more evidence supports the association between myocardial infarction (MI) and osteoarthritis (OA). The purpose of this study is to explore the shared biomarkers and pathogenesis of MI complicated with OA by systems biology.

**Methods:**

Gene expression profiles of MI and OA were downloaded from the Gene Expression Omnibus (GEO) database. The Weighted Gene Co-Expression Network Analysis (WGCNA) and differentially expressed genes (DEGs) analysis were used to identify the common DEGs. The shared genes related to diseases were screened by three public databases, and the protein-protein interaction (PPI) network was built. GO and KEGG enrichment analyses were performed on the two parts of the genes respectively. The hub genes were intersected and verified by Least absolute shrinkage and selection operator (LASSO) analysis, receiver operating characteristic (ROC) curves, and single-cell RNA sequencing analysis. Finally, the hub genes differentially expressed in primary cardiomyocytes and chondrocytes were verified by RT-qPCR. The immune cell infiltration analysis, subtypes analysis, and transcription factors (TFs) prediction were carried out.

**Results:**

In this study, 23 common DEGs were obtained by WGCNA and DEGs analysis. In addition, 199 common genes were acquired from three public databases by PPI. Inflammation and immunity may be the common pathogenic mechanisms, and the MAPK signaling pathway may play a key role in both disorders. DUSP1, FOS, and THBS1 were identified as shared biomarkers, which is entirely consistent with the results of single-cell RNA sequencing analysis, and furher confirmed by RT-qPCR. Immune infiltration analysis illustrated that many types of immune cells were closely associated with MI and OA. Two potential subtypes were identified in both datasets. Furthermore, FOXC1 may be the crucial TF, and the relationship of TFs-hub genes-immune cells was visualized by the Sankey diagram, which could help discover the pathogenesis between MI and OA.

**Conclusion:**

In summary, this study first revealed 3 (DUSP1, FOS, and THBS1) novel shared biomarkers and signaling pathways underlying both MI and OA. Additionally, immune cells and key TFs related to 3 hub genes were examined to further clarify the regulation mechanism. Our study provides new insights into shared molecular mechanisms between MI and OA.

## 1 Introduction

Myocardial infarction (MI), characterized by the erosion or rupture of unstable coronary plaques, is a common and critical cardiovascular disease (1, 2). Osteoarthritis (OA) is a musculoskeletal disorder marked by degeneration and destruction of articular cartilage, which ranks alongside cardiovascular diseases and neurological disorders as the major cause of disability and mortality worldwide (3, 4). According to previous research, patients with OA have a much higher risk of MI than the general population (5). High body mass index, abnormal glucose and lipid metabolism are also promoted (6). Moreover, it has been shown that OA-related persistent pain may contribute to MI (7). Although corticosteroids and nonsteroidal anti-inflammatory medications are recommended to alleviate the symptoms of OA, some additional hazards cannot be ignored (3). For instance, early medicines like rofecoxib were kept out of the public eye due to cardiovascular safety concerns. Currently prescribed drugs like celecoxib and naproxen are also believed to be associated with an increased prevalence of MI (8–10). Short-term, low-dose corticosteroids induce few serious cardiovascular adverse events (11), while OA usually requires long-term drug maintenance and comprehensive management, which may pose a danger to glucose and lipid metabolism (12, 13). Furthermore, an observational study involving 467,779 knee replacements showed that MI was a barrier to further reducing perioperative mortality (14). Another large cohort research in the United Kingdom also revealed a considerably higher incidence of MI after joint replacement (15). Even while MI and OA are common comorbidities, especially in the elderly, it might be challenging to treat each patient individually using the single-disease guidelines (16). Therefore, continued research into the shared pathogenesis and potential diagnostic biomarkers of MI and OA are crucial for clinical purposes.

Recently, there is increasing evidence that aging, sex, and obesity are risk factors for both OA and MI (3, 17). It is now clear that age-related oxidative stress, cellular degradation, and cumulative exposure to various risk factors may work together to promote MI and OA (18, 19). Because of the cardioprotective properties of female estrogen, younger women experience fewer cardiovascular events, whereas postmenopausal women have a significantly higher incidence of MI and OA. Multisite OA that is influenced by the timing of menopause is also known as menopausal arthritis (20). In addition, obesity is a known risk factor for cardiovascular disease, and it can also cause mechanical stress in weight-bearing joints, which is a primary cause of OA (21). Recent evidence suggests that active substances produced by adipose tissue may contribute to the development of OA (22). Although the above risk factors explain the predisposing factors to some extent, they fall short of explaining the specific pathogenesis. Notably, some studies have hypothesized that MI and OA may be connected pathologically through chronic systemic inflammation (23). Contrary to the conventional belief that OA is not inflammatory, it has increasingly come to light that chronic inflammation could promote the occurrence and progression of OA by the production of inflammatory mediators and immune cell infiltration (24, 25). Meanwhile, inflammation has greatly advanced in causing MI by contributing to the development of atherosclerosis up until acute ischemia episodes (26). Nevertheless, it is still unknown how chronic inflammation affects the precise pathogenesis behind the comorbidity of MI and OA.

**Figure 1:**
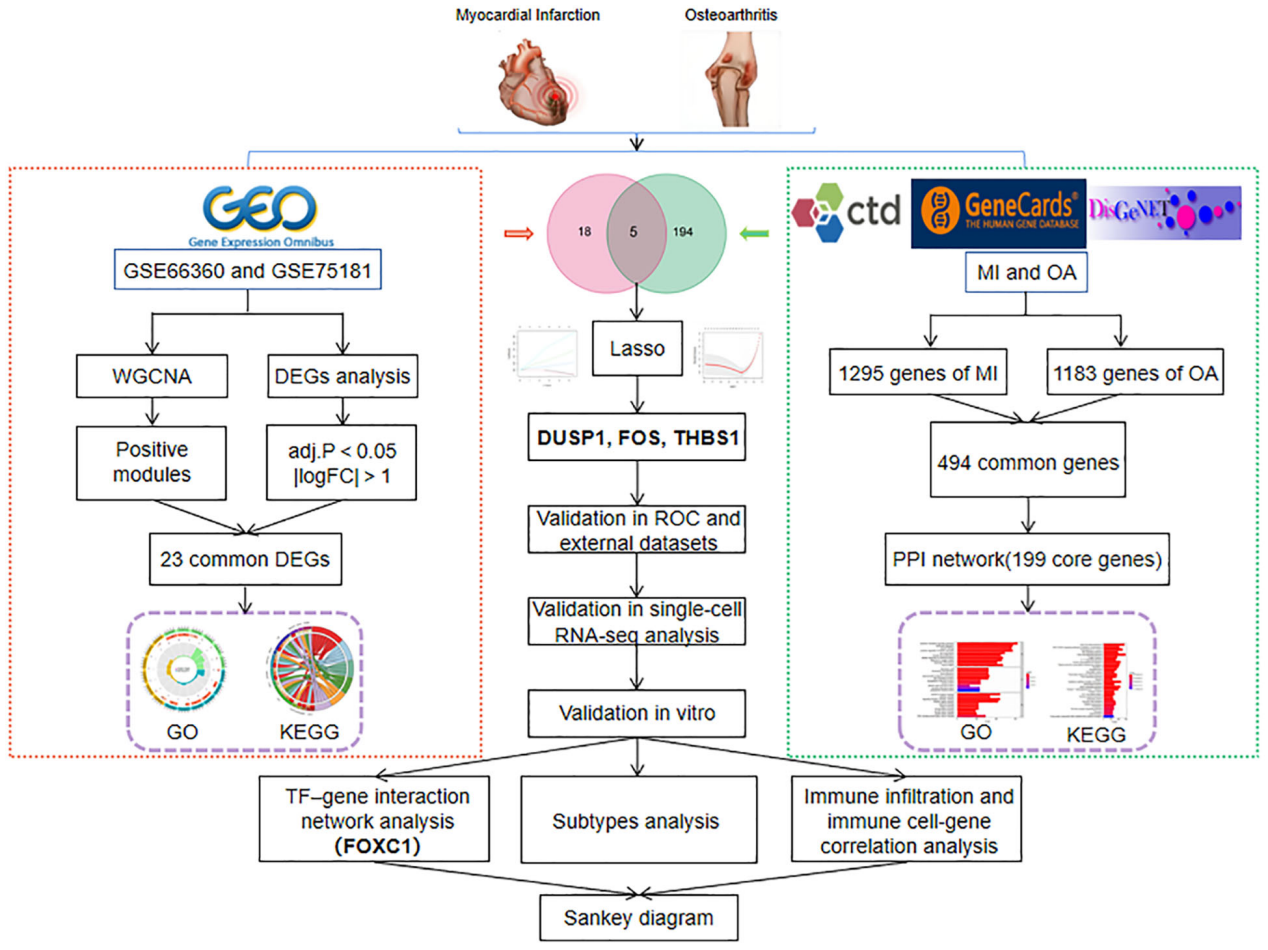
The flowchart of this research.

With the rapid development of life science and computer technology, bioinformatics analysis has made it possible to explore disease patterns in vast amounts of biological data (27). From the perspective of life as a whole, systems biology approach has the capacity to comprehend the multi-omics-based molecular mechanisms of diseases and offer suggestions for investigating probable pathogenesis and novel therapeutic approaches (28). In this study, by using integrated systems biology and bioinformatics analysis, we obtained common differentially expressed genes (DEGs) and disease-related genes between MI and OA from the Gene Expression Omnibus (GEO) database and three public disease databases (CTD, GeneCards, and DisGeNET), respectively. Then, we performed Gene Ontology (GO) and Kyoto Encyclopedia of Genes and Genomes (KEGG) enrichment analysis on the two parts of the genes to reveal the common signal pathways. The hub genes were then identified by intersection and verification. Finally, the relationship between the hub gene, transcription factors (TFs), immune cell infiltration, and subtypes analysis were predicted and visualized. The analysis flowchart of our study is shown in **Figure 1**. This study may be the first to explore the novel biomarkers and shared pathogenesis of MI and OA as comorbidity, and establish the relationship of TFs-hub genes-immune cells, which provides new insights into the combined therapeutic targets.

## 2 Materials and methods

### 2.1 Microarray datasets collection

Only datasets in which the illness group was compared with the control group or in which early disease was compared with late disease were considered, with a sample size of at least 20. We searched the GEO database (https://www.ncbi.nlm.nih.gov/geo) using the keywords “myocardial infarction” and “osteoarthritis” and then retrieved the gene expression profiling by array datasets GSE66360, GSE61144, GSE75181, and GSE55235. GSE66360 includes 49 MI patients and 50 healthy controls (Platform: GPL570 [HG-U133_Plus_2] Affymetrix Human Genome U133 Plus 2.0 Array), and GSE61144 contains 14 MI patients and 10 normal controls (Platform: GPL6106 Sentrix Human-6 v2 Expression BeadChip). GSE75181 comprises 12 OA patients and 12 normal controls (Platform: GPL10558 Illumina HumanHT-12 v4.0 Expression BeadChip), while GSE55235 contains 10 OA patients and 10 healthy controls (Platform: GPL96 [HG-U133A] Affymetrix Human Genome U133A Array). In our study, GSE66360 and GSE75181 were selected as the discovery cohort for WGCNA and DEGs analysis. GSE61144 and GSE55235 were used for the validation cohort later. The details of the involved GEO datasets are listed in **Table 1**.

**Table 1:** The details of involved GEO datasets.

ID	GSE number	Platform	PMID	Samples	Source types	Disease	Group
1	GSE66360	GPL570	28947747	49 patients vs 50 controls	endothelial cells	MI	Discovery cohort
2	GSE61144	GPL6106	26025919	14 patients vs 10 controls	peripheral blood	MI	Validation cohort
3	GSE75181	GPL10558	27275599	12 patients vs 12 controls	cartilage	OA	Discovery cohort
4	GSE55235	GPL96	24690414	10 patients vs 10 controls	synovial tissue	OA	Validation cohort

### 2.2 Weighted gene co-expression network analysis

The bioinformatics analysis tool Weighted gene co-expression network analysis (WGCNA) is utilized to find gene modules that are associated with clinical features, which can assist in identifying candidate biomarkers or targets (29). For WGCNA, 0.9 was used as the correlation coefficient threshold, and the soft-thresholding power was set to 20 after the outlier samples had been eliminated by the hierarchical clustering analysis. In order to identify essential modules, an adjacency matrix and hierarchical clustering were established. Correlation coefficients between the module and clinical characteristics in OA and MI were calculated separately, and the module with a high correlation coefficient was chosen for collecting candidate genes.

### 2.3 Identification of differential expressed genes

According to the downloaded datasets and platform files above, we annotated the data and obtained a matrix file of gene expression in each sample, and grouped each sample according to the clinical information related to it for subsequent analysis. The limma package in R was applied to normalize and analyze the GSE66360 and GSE75181 datasets. With the screening criteria of adjusted P-value < 0.05 and |log2FC| > 1, DEGs in two datasets were identified. Next, heatmap and volcano plots were drawn to visualize DEGs by applying the pheatmap R package.

### 2.4 Enrichment analysis of common DEGs

Common DEGs were identified by the intersection of the results from the above DEGs and WCGNA analysis. The colorspace, stringi, ggplot2, circlize, RColorBrewer, and ggpubr packages in R were utilized to carry out enrichment analysis of Gene Ontology (GO) and Kyoto Encyclopedia of Genes and Genomes (KEGG) pathways of common DEGs. For GO, biological processe (BP), cellular component (CC), and molecular function (MF) were enriched, and a P-value < 0.05 was considered significant. The results of the KEGG pathway analysis were also based on a P-value of 0.05. The outcomes of enrichment analysis were illustrated respectively in the loop graph and chord diagram ranked by P-value.

### 2.5 Screening common genes associated with MI and OA

In order to acquire common genes associated with MI and OA, “myocardial infarction” and “osteoarthritis” were entered into three open-source databases respectively, including CTD (http://ctdbase.org/) (30), GeneCards (https://www.genecards.org/) (31) and DisGeNET (http://www.disgenet.org) (32). The CTD database is a comprehensive genomics database in the field of comparative toxicology, which specifically concentrates on studies that investigate the connections between genes, phenotypes, and diseases. GeneCards is an extensive repository of human genes, encompassing both established and anticipated human genetic data pertaining to the genome, proteome, transcription, genetics, and their respective functions. The DisGeNET database serves as a comprehensive disease-related genetic repository, its primary objective is to facilitate the investigation of the molecular underpinnings of distinct human diseases and the examination of harmful gene attributes. Thus, genes associated with MI and OA included in all three databases were gathered, and the overlapping genes between MI and OA were further obtained by the Venn package in R.

### 2.6 Construction of protein-protein interaction network

Protein-protein interaction (PPI) network was constructed to further investigate the molecular mechanisms underlying MI and OA. Briefly, we imported common genes above into the STRING database (https://string-db.org/) (33) with organism limited to “homo sapiens”, while setting the minimum required interaction score to the highest confidence (0.9) and hiding disconnected nodes. PPI network was then visualized and analyzed by Cytoscape 3.9.0 software (https://cytoscape.org/), and the degree value of nodes was calculated by cytoHubba plugin. In our study, targets with degree value above the median were considered core genes of PPI network.

### 2.7 Enrichment analysis of core genes of PPI

GO and KEGG pathways enrichment analysis were also performed on core genes of PPI for validating the previous analysis by using the cluster profiler package in R. Similarly, P-value < 0.05 of GO and KEGG pathways were considered significantly enriched. The top 30 results of pathways and the top 10 terms of BP, CC, and MF were presented in the bubble chart ranked by P-value respectively.

### 2.8 Identification and validation of hub genes

When the common DEGs and core genes of PPI were intersected, overlapping candidate genes were obtained. Subsequently, least absolute shrinkage and selection operator (LASSO) regression was performed to determine the optimal variables from candidate genes, namely the hub genes. To further validate the accuracy of the hub genes, the receiver operating characteristic (ROC) curve was established to assess their sensitivity and specificity, and the diagnostic values were then validated in GSE61144 and GSE55235. We calculated the area under the curve (AUC) and 95% CI, while AUC > 0.7 was considered ideal.

### 2.9 ScRNA-seq dataset processing

MI single-nucleus RNA sequencing (snRNA-seq) dataset (34) was obtained from the CELLxGENE database (https://cellxgene.cziscience.com/). OA single-cell RNA sequencing (scRNA-seq) dataset (35) was obtained from the GEO database. The R software was used for single-cell analysis. Samples in OA scRNA-seq dataset were read with Seurat package. Low quality cells were filtered through the following criteria: (1) features < 200 and > 5000; (2) mitochondrial genes >10%. 2000 highly variable genes were identified for principal component analysis (PCA) after normalization of data. Then, samples were integrated using the first 30 principal components in PCA via the Harmony package. Subsequently, cells were categorized into cell clusters using the “FindNeighbors” and the “FindClusters” functions. Uniform manifold approximation and projection (UMAP) was applied to downscale the data.

### 2.10 Expression of hub genes in single-cell dataset

Genes specifically expressed in one cell cluster compared to others were identified with the “FindMarkers” function. Using these genes as markers for cell clusters, each cell cluster was annotated with reference to the CellMarker database (http://bio-bigdata.hrbmu.edu.cn/CellMarker), which is an updated database of cell markers identified by previous research (36). The obtained MI snRNA-seq dataset has been completed with preprocessing. The data was read and the cells were categorized into different cell types. The expression of the markers in each cell clusters was viewed by a dot plot to verify the reliability of the clustering. The single-cell dataset was then divided into a patient subset and a control subset. Nebulosa package was used to visualize the relative expression of 3 hub genes in each cell cluster. The expression of hub genes was scored with the “AddModuleScore” function, and differences in scoring between subsets were visualized by violin plots.

### 2.11 Cell isolation and culture

All animal experimental procedures were in accordance with the institutional and international guidelines and approved by the Ethics Committee of the First Affiliated Hospital of Guangzhou University of Chinese Medicine (GZTCMF1-20240009). Cardiac cell injury and death generated by hypoxia stimulation has been used as an appropriate in vitro model to study MI, according to previous research (37, 38). The hearts of neonatal Sprague Dawley (SD) rats were rapidly removed and washed instantly in cold phosphate-buffered saline (PBS, Biosharp, China) solution. Both ventricles were cut into l to 2 mm^3^ and dissociated in 0.25% trypsin (Solarbio, China) at 37°C for 1-2 min, then discard the supernatant carefully. Next, 0.1% type II collagenase solution (Solarbio, China) was added to digest the myocardial tissue several times until the digestion was complete. Cell suspensions were shifted out and neutralized with complete medium. Then all suspensions were pelleted by centrifugation at 1200 rpm for 5 min. The isolated cells were resuspended in DMEM/F12 (Gibco, USA) supplemented with 10% fetal bovine serum (FBS, Gibco, USA) and penicillin (100 U/ml)/streptomycin (100 U/ml), transferred into culture flask and cultured at 37°C in humid air with 5% CO_2_. After 90 min for fibroblast adherence, neonatal cardiomyocytes were plated into a 6-well plate at a density of 3×10^5^ cells per well. After 48 h culture, a sugar-free and serum-free medium was replaced to mimic nutrient deprivation, and neonatal cardiomyocytes were cultured in a hypoxic chamber (5% CO_2_ and 95% N_2_) for 9 hours (39).

The primary chondrocytes were obtained from the articular cartilage of neonatal SD rats and dealed with IL-1β according to previous research (40, 41). The knee cartilage was cut into 1 mm^3^ small pieces and then incubated with 0.25% trypsin (Solarbio, China) for 40 min, followed by incubation with 0.2% type II collagenase (Solarbio, China) for 2 h, the cells were collected and cultured in DMEM/F12 medium containing 10% FBS in a humidified atmosphere of 5% CO_2_ at 37°C. Then cells were plated at a density of 3×10^5^ cells/ml in a 6-well plate, and the media were changed every 2-3 days. Cells at 80-90% confluency were passaged using 0.25% trypsin-EDTA solution. Only chondrocytes from passage two were used in our study. After 24h, chondrocytes were washed with PBS, and 10 ng/mL IL-1β (Proteintech Group, Chicago, USA) were added and incubated for 48 h.

### 2.12 Quantitative real‐time PCR

After total RNA was isolated from cardiomyocytes and chondrocytes following the manufacturer’s instructions (Accurate Biology, Hunan, China). Reverse transcription was carried out using the Evo M-MLV RT Premix (Accurate Biology, Hunan, China) under the manufacturer’s instructions. SYBR Green Premix Pro Taq HS qPCR Kit (Accurate Biology, Hunan, China) was used for quantitative real-time PCR on QuantStudio™ 5 Real-Time PCR System (Thermo Fisher Scientific, USA), and results were analyzed using the 2^-ΔΔCt^ technique with GAPDH serving as an internal control for normalization. The primer sequences used in RT-qPCR were shown as follows: FOS (Forward: 5’-GACCATGTCAGGCGGCAGAG-3’; Reverse: 5’-GCAGCCATCTTATTCCTTTCCCTTC-3’), DUSP1 (Forward: 5’-GACAACCACAAGGCAGACATTAGC-3’; Reverse: 5’-ACAAACACCCTTCCTCCAGCATC-3’), THBS1 (Forward: 5’-AATGTGGTGCGTGTCCTCCTG-3’; Reverse: 5’-CCGATGTTCTCCGTTGTGATTGAAG-3’), FOXC1 (Forward: 5’-GACATCAAGACGGAGAACGGTACG-3’; Reverse: 5’-GGCTGCTGCTGCTGCTGTC-3’), GAPDH (Forward: 5’-GACATGCCGCCTGGAGAAAC-3’; Reverse: 5’-AGCCCAGGATGCCCTTTAGT-3’).

### 2.13 Construction of TFs-hub genes network

The JASPAR database (https://jaspar.genereg.net/) is a public website containing many TF-target regulatory relationships derived from published collections of humans to identify the key regulators (42). 3 hub genes were imported to predict the TFs-hub genes interaction network, which was visualized in Cytoscape 3.9.0 software. And the Scan tool in JASPAR was used to identify transcription factor binding sites for 3 hub genes.

### 2.14 Immune infiltration analysis and correlation with hub gene

To investigate the infiltration status of various immune cells of MI and OA, the CIBERSORT algorithm was used to quantify the levels of 22 different immune cell types, and the vioplot package in R was utilized for visualization. Then, the correlation matrix of 22 immune cell type proportions was constructed. Spearman correlation analysis was performed to explore how closely the expression of the hub genes related to immune cell enrichment. Finally, by using the ggalluvial package in R, a Sankey diagram was drawn to illustrate the relationship of TFs-hub genes-immune cells.

### 2.15 Identification of potential subtypes

Consensus clustering with K-means algorithms was applied to identify 3 hub genes-related subtypes correlated with gene expression, and the “ConsensuClusterPlus” package was adopted for the quantity and robustness of clusters to be determined with a consensus clustering algorithm realized.

### 2.16 Statistical analysis

The R programming language was applied to perform our bioinformatics analyses. Statistical analysis of different groups was compared by using Student’s t-test, P < 0.05 is considered statistically significant.

## 3 Results

### 3.1 Weighted gene co-expression network analysis

**Figure 2:**
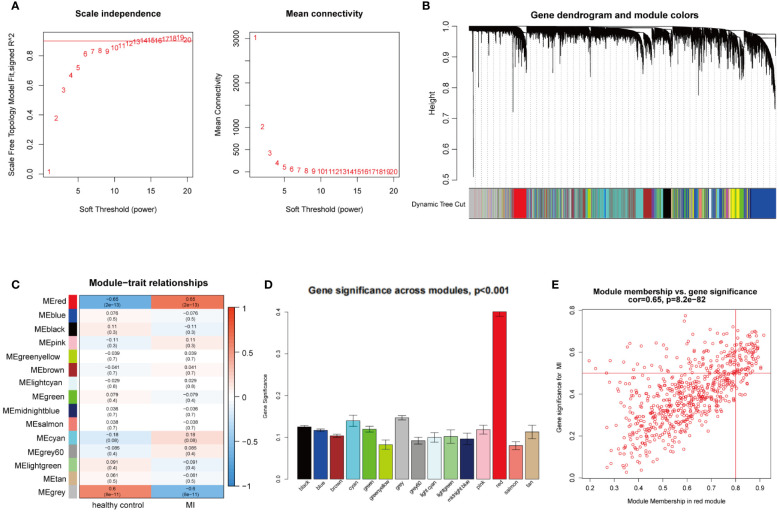
WGCNA analysis of the significant modules in MI. **(A)** Determination of the optimal soft thresholds for GSE66360. **(B)** Gene co-expression modules represented by different colors under the clustering tree. **(C)** Identification of highly correlated modules between MI and healthy controls. **(D)** Distribution of average gene significance in the each module. **(E)** Correlation plot between module membership and gene significance of genes included in the red module.

**Figure 3:**
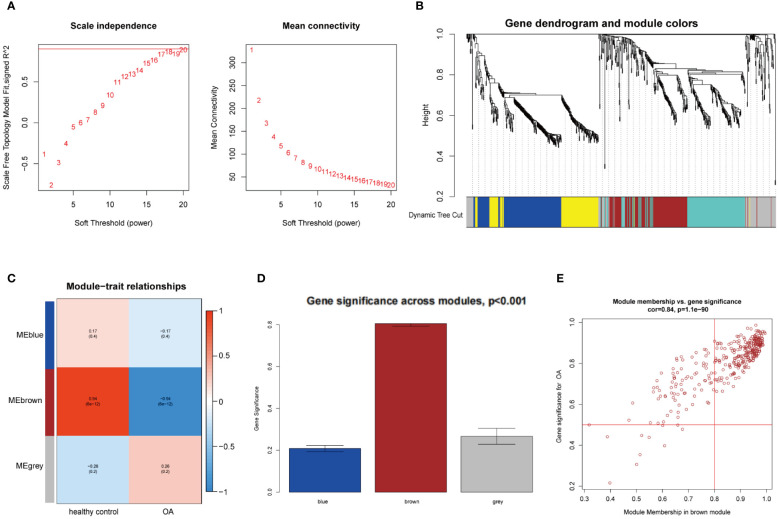
WGCNA analysis of the significant modules in OA. **(A)** Determination of the optimal soft thresholds for GSE75181. **(B)** Gene co-expression modules represented by different colors under the clustering tree. **(C)** Identification of highly correlated modules between OA and healthy controls. **(D)** Distribution of average gene significance in the each module. **(E)** Correlation plot between module membership and gene significance of genes included in the brown module.

WGCNA was utilized to investigate the link between key genes and clinical traits in MI and OA. For GSE66360, 12 was the ideal soft-thresholding power when R^2^ > 0.9 (**Figure 2A**). A total of 15 modules were identified after merging the associated modules, which was displayed by the clustering tree (**Figure 2B**). The red module had the strongest positive relation with MI (r = 0.65) in the module-clinical trait relationships heatmap (**Figure 2C**). In **Figure 2D**, the average gene significance in each module was estimated. We then chose the red module to build the correlation plot between module membership and gene significance, and 671 genes were obtained (cor = 0.65, P = 8.2e-82) (**Figure 2E**). In addition, we discovered that 18 was the optimal soft-thresholding power for GSE75181 when R^2^ > 0.9 (**Figure 3A**). The clustering tree was classified into 3 modules, among which the brown module had the highest correlation with OA (r = -0.94) (**Figures 3B, C**). The brown module was selected because of the greatest significance (**Figure 3D**), from which 336 genes were identified (cor = 0.84, P = 1.1e-90) (**Figure 3E**).

### 3.2 Identification of common DEGs of MI and OA

**Figure 4:**
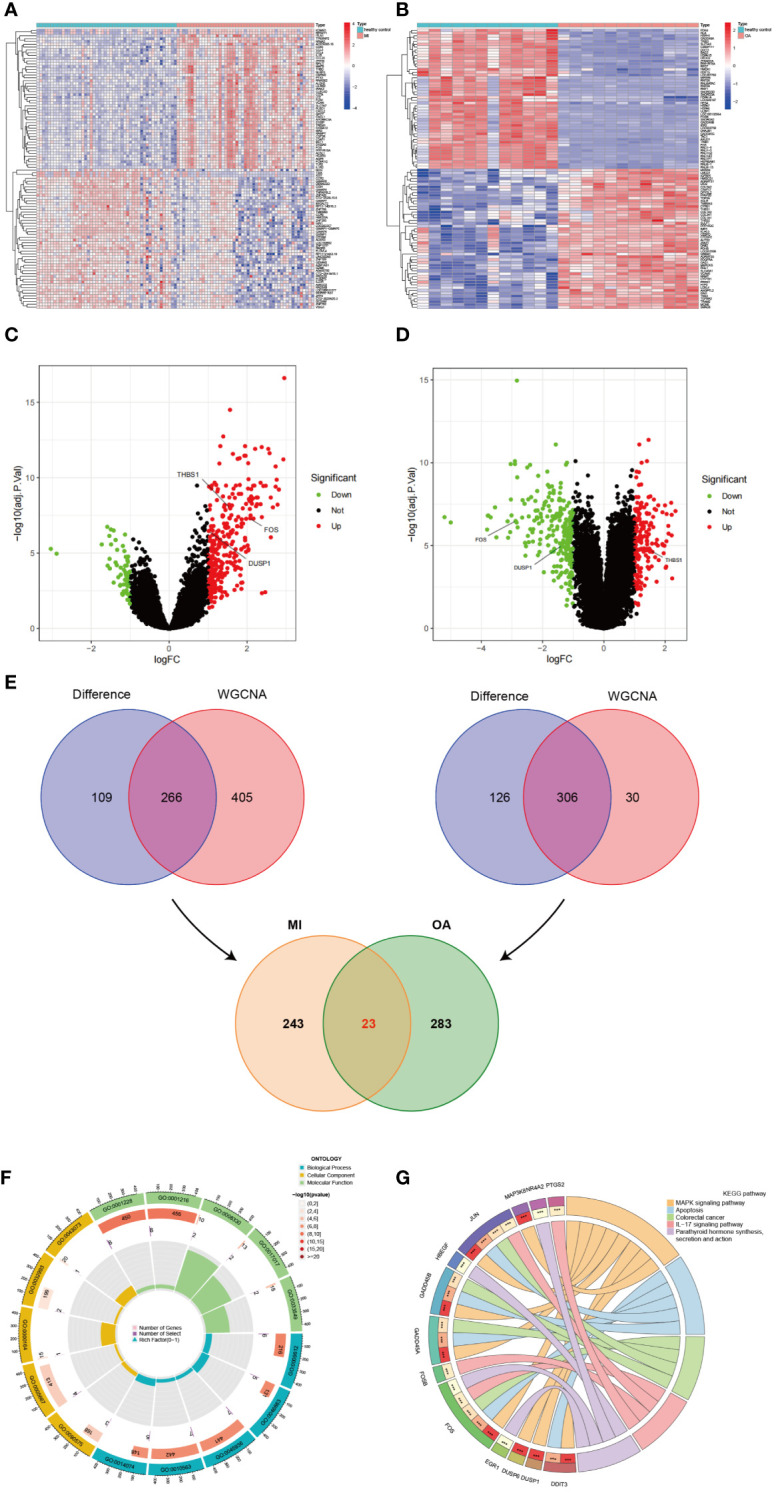
Identification of common DEGs and enrichment analysis. **(A)** Heatmap of all DEGs in GSE66360. **(B)** Heatmap of all DEGs in GSE75181. **(C)** Volcano plots of all DEGs in GSE66360. **(D)** Volcano plots of all DEGs in GSE75181. **(E)** Identification of common DEGs between MI and OA. **(F)** The loop graph showing significantly enriched top 5 GO (BP, CC, and MF) terms associated with common DEGs. **(G)** The chord diagram showing significantly enriched top 5 signaling pathways in KEGG and distribution of DEGs in each pathway.

According to the previous criteria, a total of 375 DEGs (309 upregulated and 66 downregulated) were identified in GSE66360 and visualized by heatmap and volcano plots (**Figures 4A, C**). Moreover, a total of 432 DEGs (189 upregulated and 243 downregulated) were identified in GSE75181, which also was represented in heatmap and volcano plots (**Figures 4B, D**). As shown in the Venn diagram (**Figure 4E**), DEGs were intersected to the results of WGCNA analysis in MI and OA respectively, and the overlapped genes of two parts were intersected again, 23 genes (PPP1R15A, SLC7A5, DDIT3, HBEGF, TRIB1, NR4A2, FOS, GADD45A, JUN, FOSB, MARCKS, MXD1, RLF, GADD45B, DUSP1, THBS1, CEBPD, EGR1, IER3, DUSP6, PTGS2, MAP3K8, and RGS2) were finally identified as the common DEGs of MI and OA.

### 3.3 Enrichment analysis of common DEGs

GO and KEGG enrichment analyses were performed on common DEGs to reveal the biological functions and demonstrate the underlying molecular interactions between MI and OA. A total of 597 GO terms were obtained by applying R packages, which consisted of 532 BP terms, 10 CC terms, and 55 MF terms. The top 5 terms of BP, CC, and MF were visualized in the loop graph with the minimum P-value (**Figure 4F**) and presented in **Supplementary Table S1**. BP enrichment terms mainly contained response to mechanical stimulus, response to organophosphorus, negative regulation of phosphate metabolic process, negative regulation of phosphorus metabolic process, and response to purine-containing compound. CC terms mainly included RNA polymerase II transcription regulator complex, transcription regulator complex, protein phosphatase type 1 complex, protein-DNA complex, and germ cell nucleus. DNA-binding transcription activator activity/RNA polymerase II-specific, DNA-binding transcription activator activity, protein tyrosine/threonine phosphatase activity, and MAP kinase tyrosine/serine/threonine phosphatase activity were the significantly enriched MF terms. Besides, a total of 59 pathway terms were identified, and the top 5 pathway terms were shown in the chord diagram ranked by P-value (**Figure 4G**) and listed in **Supplementary Table S2** (dual-positive). KEGG pathway enrichment terms indicated that common DEGs were mainly involved in MAPK signaling pathway, IL-17 signaling pathway, TNF signaling pathway, Oxytocin signaling pathway, and p53 signaling pathway.

### 3.4 Identification of common genes associated with MI and OA

**Figure 5:**
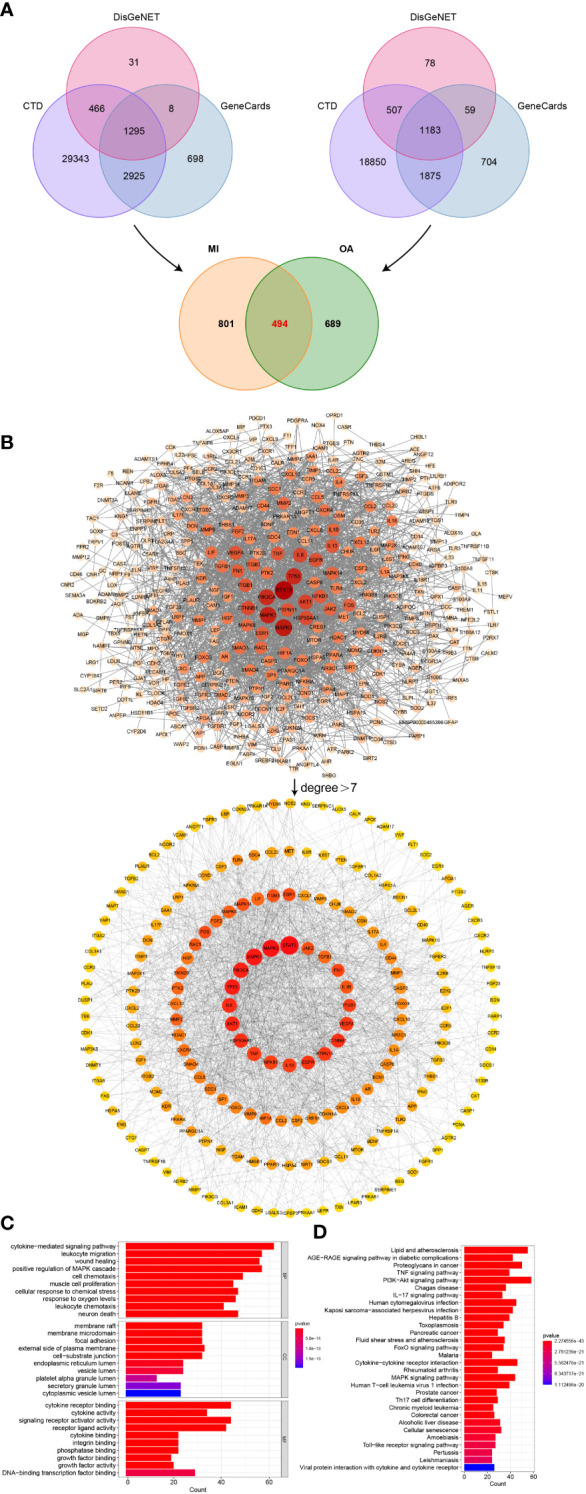
Identification of shared genes related to diseases, PPI network, and enrichment analysis. **(A)** Identification of common genes between MI and OA from three public disease databases. **(B)** Analysis of PPI network, the core genes were obtained with degree value greater than 7. **(C)** Top 10 terms in GO (BP, CC, and MF) enrichment analysis of the core genes. **(D)** Top 30 results in KEGG pathway enrichment analysis of the core genes.

CTD, GeneCards, and DisGeNET databases were searched by using the keywords “myocardial infarction” and “osteoarthritis” respectively, and only overlapped genes were saved. We then acquired 1295 genes associated with MI and 1183 genes associated with OA, then 494 common genes between MI and OA were identified in **Figure 5A**.

### 3.5 PPI network analysis

In order to determine the core genes, the degree values of the PPI network between 494 common genes were analyzed. We then obtained a network with 405 nodes and 2349 edges by setting the highest confidence level (0.9) and hiding the unconnected nodes. Degree values were calculated using the cytoHubba plugin in Cytoscape software, and the median degree was 7. Node sizes and color shades showed positive correlations ranked by degree values, and a total of 199 core genes in the PPI network were discovered to have degree values greater than 7 (**Figure 5B**). The corresponding degree values were listed in **Supplementary Table S3**.

### 3.6 Enrichment analysis of core genes of PPI

To further explore the biological functions and validate our results presented above, we also performed GO and KEGG enrichment analysis on the core genes of PPI. According to the bar graph, the results of functional enrichment and pathway analysis were ranked by P-value (**Figure 5C**), cytokine-mediated signaling pathway, leukocyte migration, wound healing, positive regulation of MAPK cascade, response to oxygen levels and leukocyte chemotaxis were the mainly enriched terms of BP, and membrane raft, membrane microdomain, focal adhesion, external side of plasma membrane and cell-substrate junction were primarily involved in CC terms, MF terms mainly contained cytokine receptor binding, cytokine activity, signaling receptor activator activity, and receptor ligand activity. Additionally, the significantly enriched pathway terms of KEGG were MAPK signaling pathway and so on, which indicated similar results to our previous analysis (**Figure 5D**).

### 3.7 Identification and validation of the hub genes

**Figure 6:**
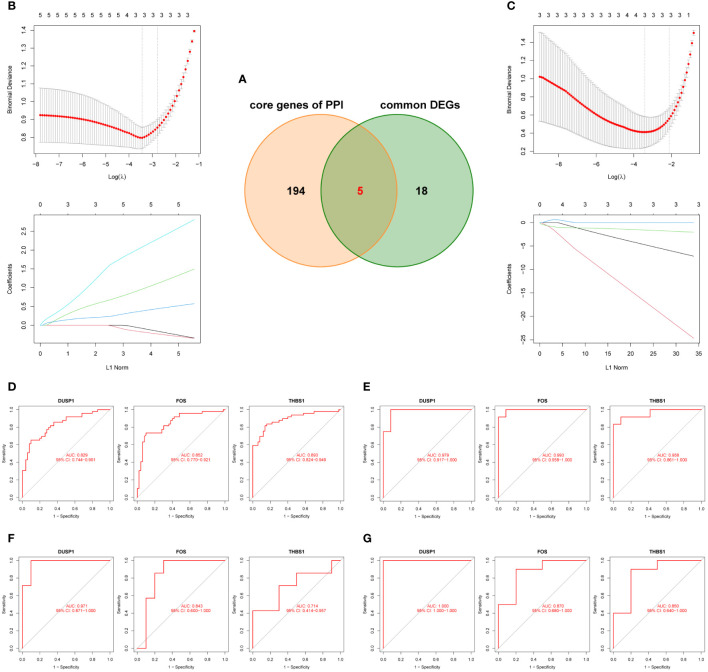
Identification and validation of the hub genes. **(A)** Five candidate genes were obtianed by intersecting common DEGs to the core genes of PPI in the Venn diagram. **(B, C)** LASSO regression analysis for screening the hub genes in GSE66360 and GSE75181, cross-validation was performed to select the best λ, and the lines in the regression coefficient path map represented the variables included and their running trajectories. **(D, E)** ROC curves of DUSP1, FOS, and THBS1 to assesse for diagnostic sensitivity and specificity value in GSE66360 and GSE75181. **(F, G)** Validation the clinical utility of three hub genes in GSE61144 and GSE55235.

After taking the intersection of the PPI core genes and common DEGs, 5 candidate hub genes (DUSP1, FOS, THBS1, EGR1, and PTGS2) were obtained (**Figure 6A**). Meanwhile, all candidate hub genes were subjected to LASSO regression analysis, which is characterized by penalizing the absolute value of a regression coefficient, DUSP1, FOS, and THBS1 were then chosen as the final hub genes (**Figures 6B, C**). Next, the diagnostic sensitivity and specificity of 3 hub genes were evaluated using ROC curves, the AUC of all hub genes was greater than 0.7 (**Figures 6D, E**), indicating a high diagnostic value for both MI and OA. Furthermore, GSE61144 and GSE55235 were used to validate the clinical utility of 3 hub genes, and all the AUC were also higher than 0.7 (**Figures 6F, G**). Among them, DUSP1 showed the best diagnostic efficiency for MI (AUC = 0.971) and OA (AUC = 1.000). These results suggested that 3 hub genes could be promising diagnostic biomarkers for both MI and OA.

### 3.8 Differential expression of hub genes among groups in single-cell dataset

**Figure 7:**
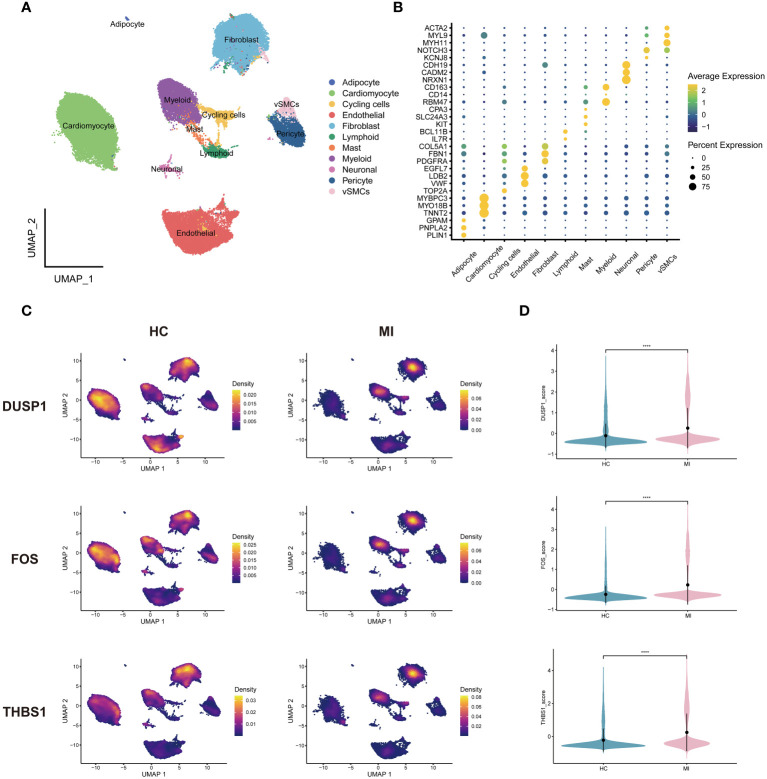
Single-cell analysis of MI dataset. **(A)** Cells were categorized and annotated into 11 cell types. **(B)** Dot plot of markers for cell clusters. **(C)** Density plots of the 3 genes expressed in MI and HC samples. **(D)** Violin plots of the 3 genes between groups. vSMCs, vascular smooth muscle cells; HC, healthy controls.

The cells in MI snRNA-seq dataset were divided into adipocyte, cycling cells, endothelial cells, fibroblasts, mast cells, myeloid, neuronal, pericyte, vascular smooth muscle cells (vSMCs), cardiomyocytes and lymphoid (**Figure 7A**). Referring to CellMarker, ACTA2, MYL9 and MYH11 were used as markers for vSMCs; NOTCH3 and KCNJ8 as markers for pericyte; CDN19, CADM2, and NRXN1 as markers for neuronal; CD163, CD14, and RBM47 as markers for myeloid; CPA3, SLC24A3, and KIT as markers for mast cells; BCL11B, and IL7R as markers for lymphoid; COL5A1, FBN1, and PDGFRA as markers for fibroblasts; EGFL7, LDB2, and VWF as markers for endothelial cells; TOP2A as marker for cycling cells; MYBPC3, MYO18B, and TNNT2 as markers for cardiomyocytes; GPAM, PNPLA2, and PLIN1 as markers for adipocyte. Dot plot in **Figure 7B** shows differences in the expression of markers between different cell clusters. Density plots show the different expression trends of 3 genes between the disease and control in MI (**Figure 7C**). In the MI snRNA-seq dataset, the expression of 3 hub genes was relatively dispersed in the healthy controls. While in the MI samples, it was concentrated in fibroblasts and myeloid. Violin plots show that the expression levels of all 3 genes were elevated in MI samples compared to healthy controls (**Figure 7D**).

**Figure 8:**
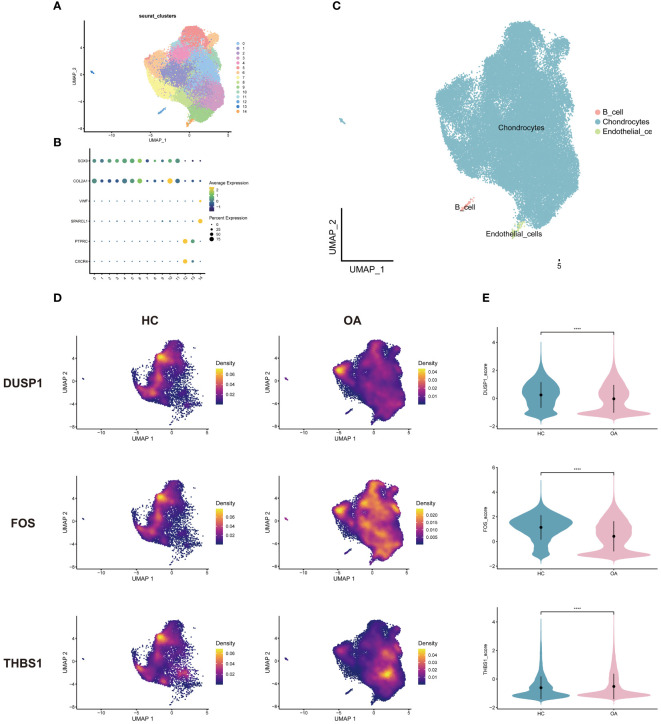
Single-cell analysis of OA dataset. **(A)** Cells were categorized into 15 clusters. **(B)** Dot plot of markers for cell clusters. **(C)** Cell clusters were annotated into 3 cell types. **(D)** Density plots of the 3 genes expressed in OA and HC samples. **(E)** Violin plots of the 3 genes between groups. HC, healthy controls.

After quality control and downscaling, the cells in OA scRNA-seq dataset were categorized into 15 clusters (**Figure 8A**). Referring to CellMarker, SOX9 and COL2A1 were used as markers for chondrocytes; VWF and SPARCL1 as markers for endothelial cells; CXCR4 and PTPRC as markers for B cells. Dot plot in **Figure 8B** shows differences in the expression of markers between different cell clusters. 3 annotated cell clusters were visualized with the UMAP plot (**Figure 8C**). In OA dataset, 3 hub genes were mainly expressed in chondrocytes (**Figure 8D**). And 3 hub genes were concentrated in different chondrocyte subpopulations in OA samples and healthy controls, suggesting that certain subpopulations are more involved in the disease process. Among the 3 genes, DUSP1 and FOS were highly expressed in healthy samples and THBS1 was highly expressed in OA samples (**Figure 8E**).

**Figure 9:**
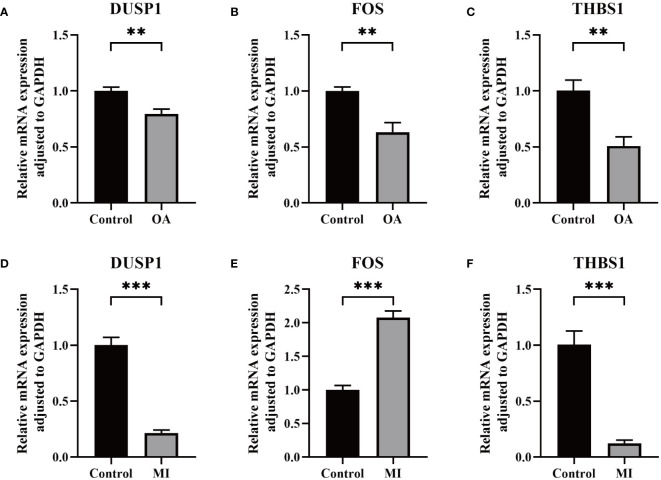
Validation of key biomarkers. **(A–C)** The RNA expression of FOS, DUSP1, and THBS1 in OA and control group. **(D-F)** The RNA expression of FOS, DUSP1, and THBS1 in MI and control group. P < 0.01: **, P < 0.001: ***.

Through the RT-qPCR validation, the mRNA expression levels of 3 genes were significantly different. Compared with the control group, DUSP1, FOS, and THBS1 were all significantly down-regulated in OA samples (**Figures 9A-C**). On the other hand, significantly lower expression of DUSP1 and THBS1 and higher expression of FOS were observed in MI samples compared to controls (**Figures 9D-F**).

### 3.9 Analysis of TFs–genes network

**Figure 10:**
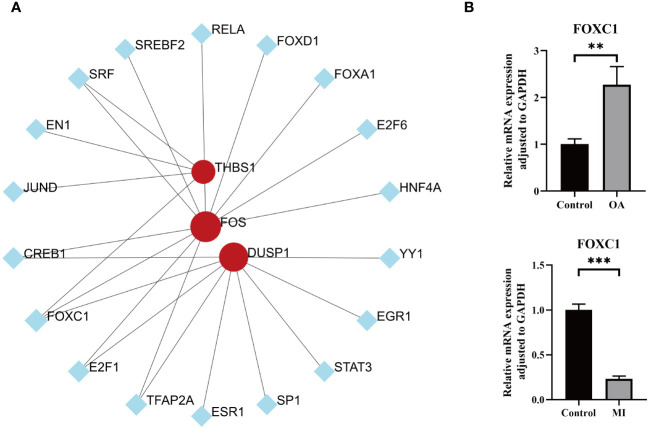
TFs prediction and validation. **(A)** TFs-hub genes interaction network analysis, round dots indicating the hub genes, square dots indicating TFs, bigger size of round dots indicating association with a greater number of TFs. **(B)** Expression levels of key transcription factor between OA/MI and control. P < 0.01: **, P < 0.001: ***.

To better understand the key TFs that regulate hub genes at the transcriptional level, we examined the link between TFs and the hub genes and built a TFs-genes network for visualization. As shown in **Figure 10A**, a total of 18 TFs interacted with 3 hub genes. TFAP2A, E2F1, CREB1, and SRF were associated with 2 hub genes, and FOXC1 were regulating all the hub genes simultaneously, which may play a key role in regulating the progression of MI and OA. This was confirmed by our empirical experiments, in which FOCX1 was significantly differentially expressed in both disease groups, suggesting its possible involvement in regulating the progression of MI and OA (**Figure 10B**). As shown in **Supplementary Figure S1**, MA0032.1.FOXC1 and MA0032.2.FOXC1 were recognized as binding regions, and a total of 175 binding sites were identified for 3 hub genes with transcription factors (**Supplementary Table S9**). In addition, the FOS gene was regulated by multiple TFs in the meantime, this may be due to easier identification.

### 3.10 Association between the hub genes and immune infiltration

**Figure 11:**
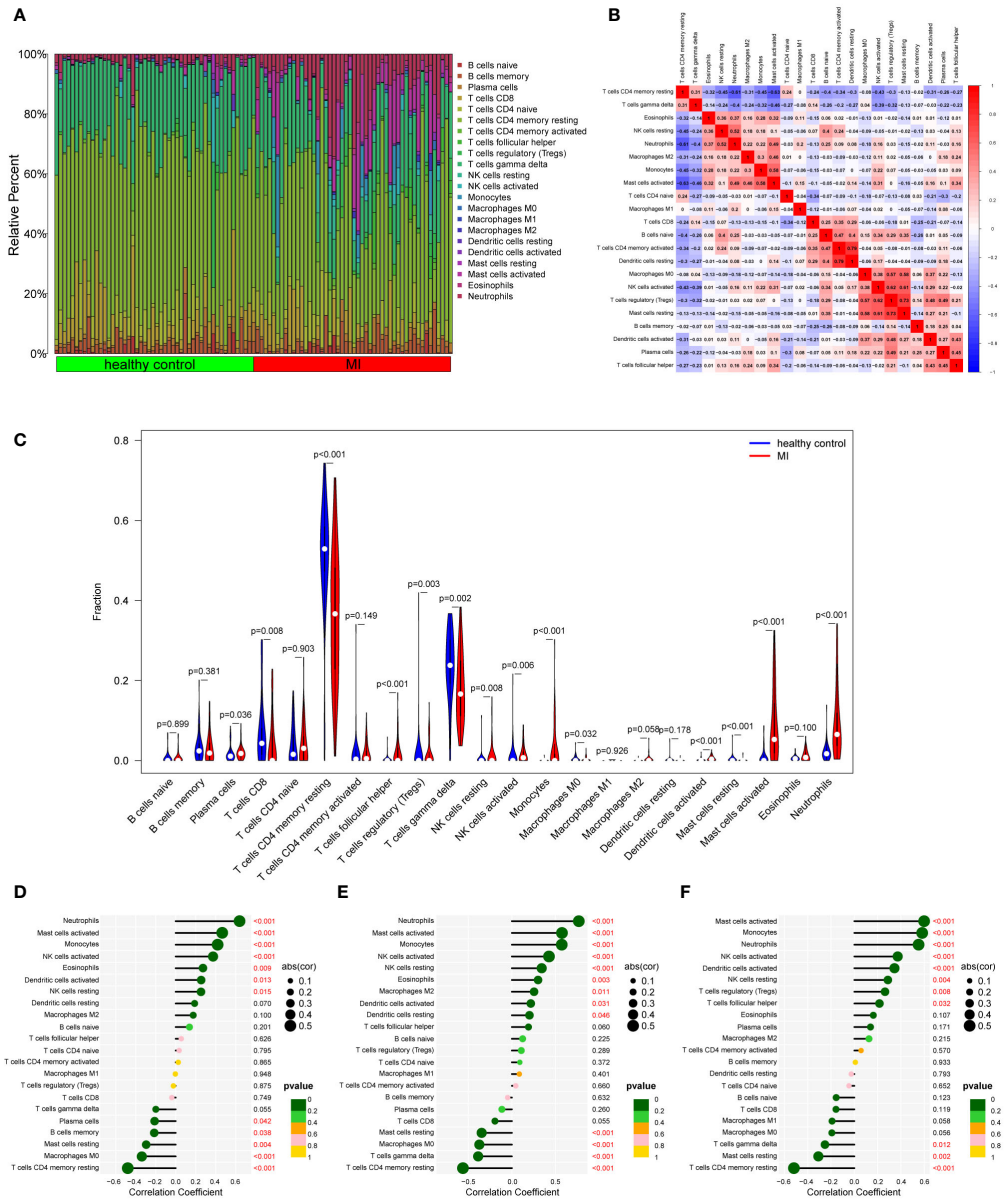
Immune cell infiltration analysis between MI and control. **(A)** The proportion of 22 kinds of immune cells between the two groups. **(B)** Comparison of differential infiltration among 22 immune cells. **(C)** Correlation of 22 immune cell type compositions. **(D-F)** The correlations between the expression of three hub genes (DUSP1, FOS, and THBS1) and immune cell enrichment.

**Figure 12:**
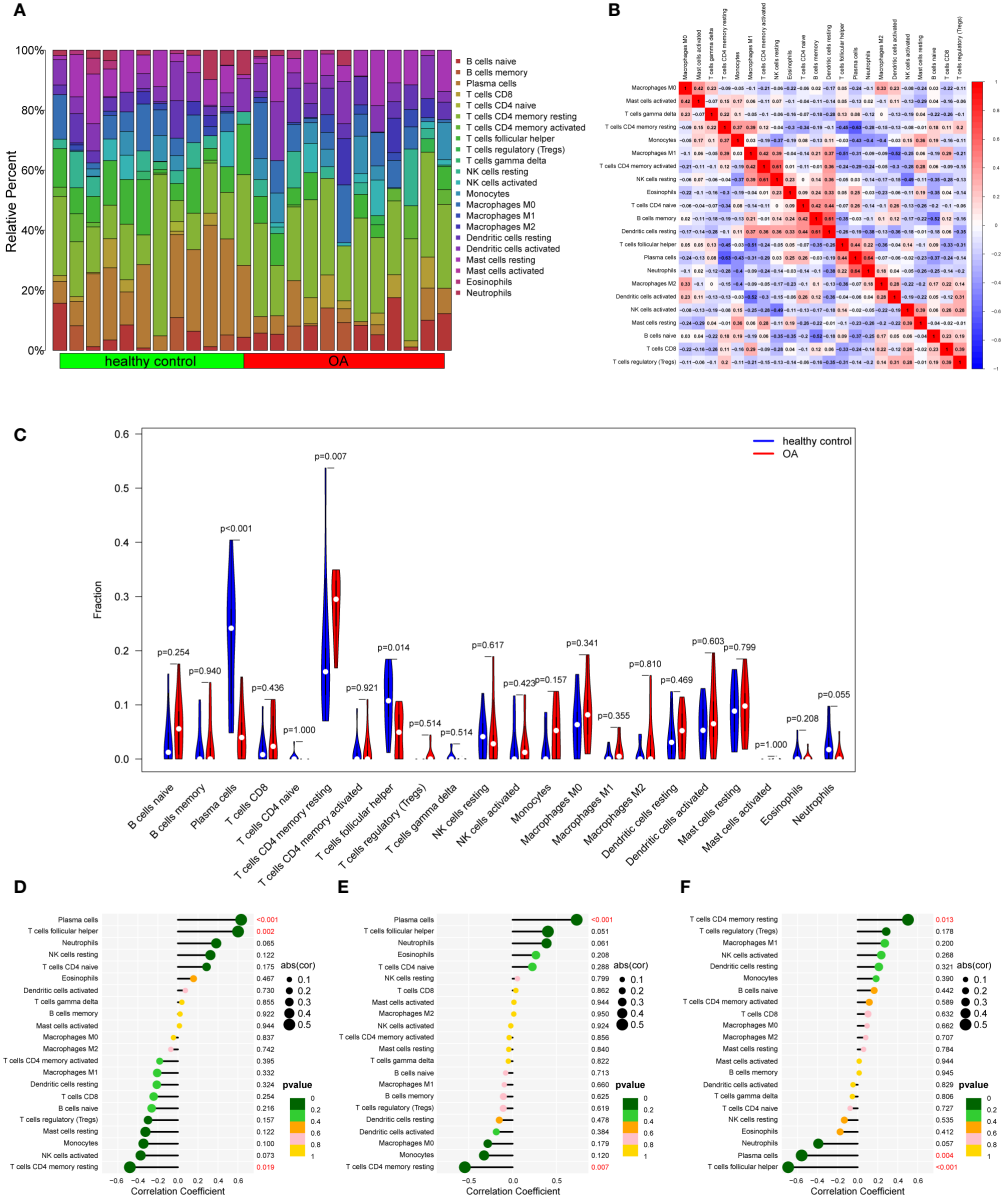
Immune cell infiltration analysis between OA and control. **(A)** The proportion of 22 kinds of immune cells between the two groups. **(B)** Comparison of differential infiltration among 22 immune cells. **(C)** Correlation of 22 immune cell type compositions. **(D-F)** The correlations between the expression of three hub genes (DUSP1, FOS, and THBS1) and immune cell enrichment.

**Figure 13:**
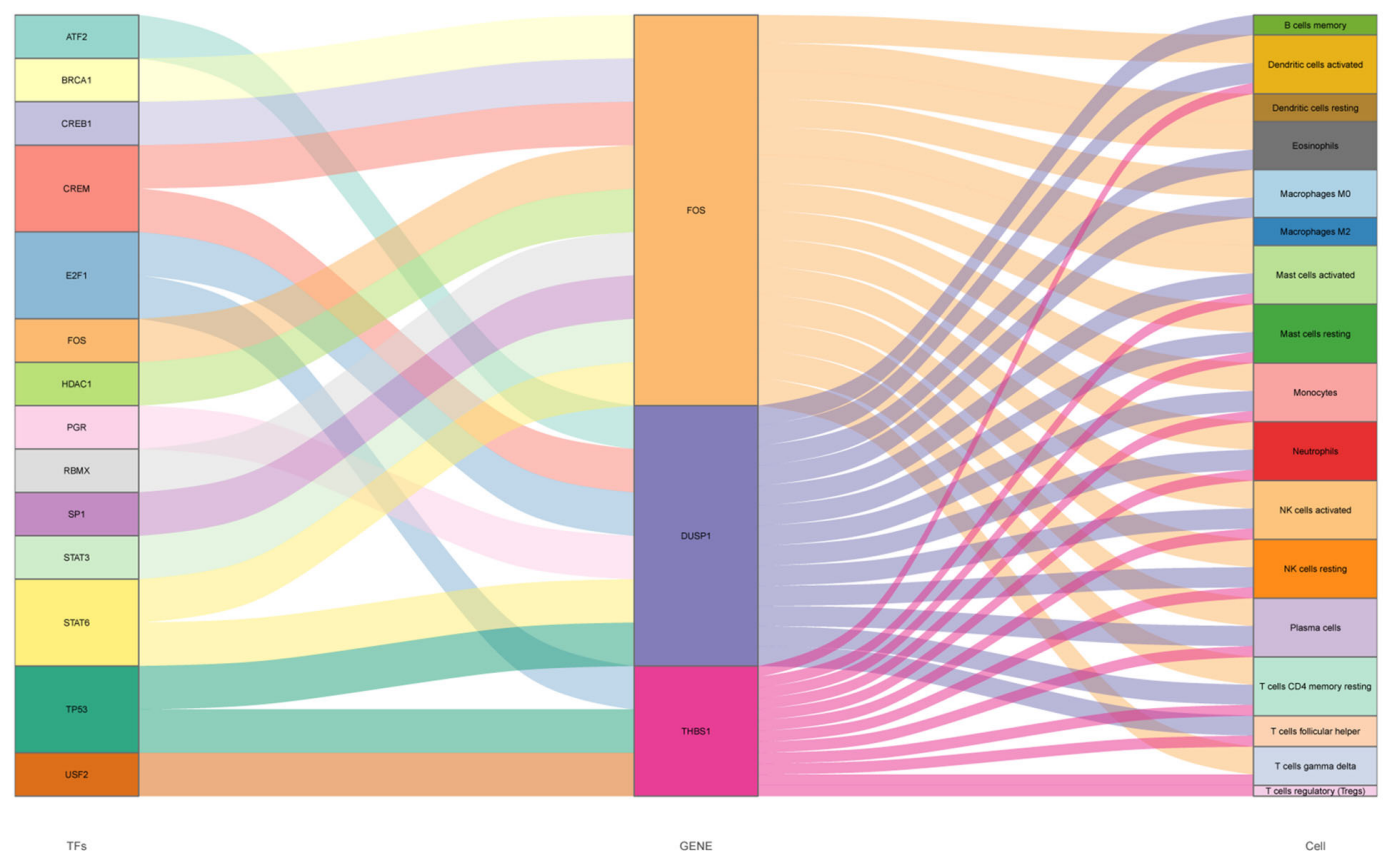
Sankey diagram of the TFs-hub genes-immune cells network, three columns from left to right indicating TFs, hub genes and immune cells respectively, the size of each rectangle representing the degree of connectivity.

To our knowledge, immune response and inflammation are closely related to the pathogenesis of MI and OA comorbidity. Hence, immune infiltration analysis was carried out to further reveal the immune microenvironment differences by adopting the CIBERSORT algorithm. The proportions of 22 types of immune cells between healthy people and MI are shown in **Figure 11A**. Compared to healthy people, the fractions of plasma cells, T cells follicular helper, T cells gamma delta, NK cells resting, monocytes, dendritic cells activated, mast cells activated and neutrophils were relatively greater in MI, while T cells CD8, T cells CD4 memory resting, T cells regulatory (Tregs), NK cells activated, Macrophages M0 and mast cells resting were relatively less (p < 0.05) (**Figure 11C**). The correlation matrix was constructed to a close negative or positive connection between each type of immune cell (**Figure 11B**). The Spearman correlations between the expression of three hub genes and immune cell enrichment were presented in the lollipop graph (**Figures 11D-F**). Between OA and healthy control, the differential proportions and correlation matrix of each type of immune cells were shown in **Figures 12A, B**. Plasma cells and T cells follicular helper were relatively less in OA, but T cells CD4 memory resting was significantly greater (p < 0.05) (**Figure 12C**). Spearman correlation analysis was also performed to detect the correlation between the expression of three hub genes and immune cell enrichment in OA (**Figures 12D-F**). Finally, as shown in **Figure 13**, the relationship of TFs-hub genes-immune cells was visualized by the Sankey diagram, which may be components to discovering the pathogenesis between MI and OA.

### 3.11 Analysis of subtypes in GEO datasets

**Figure 14:**
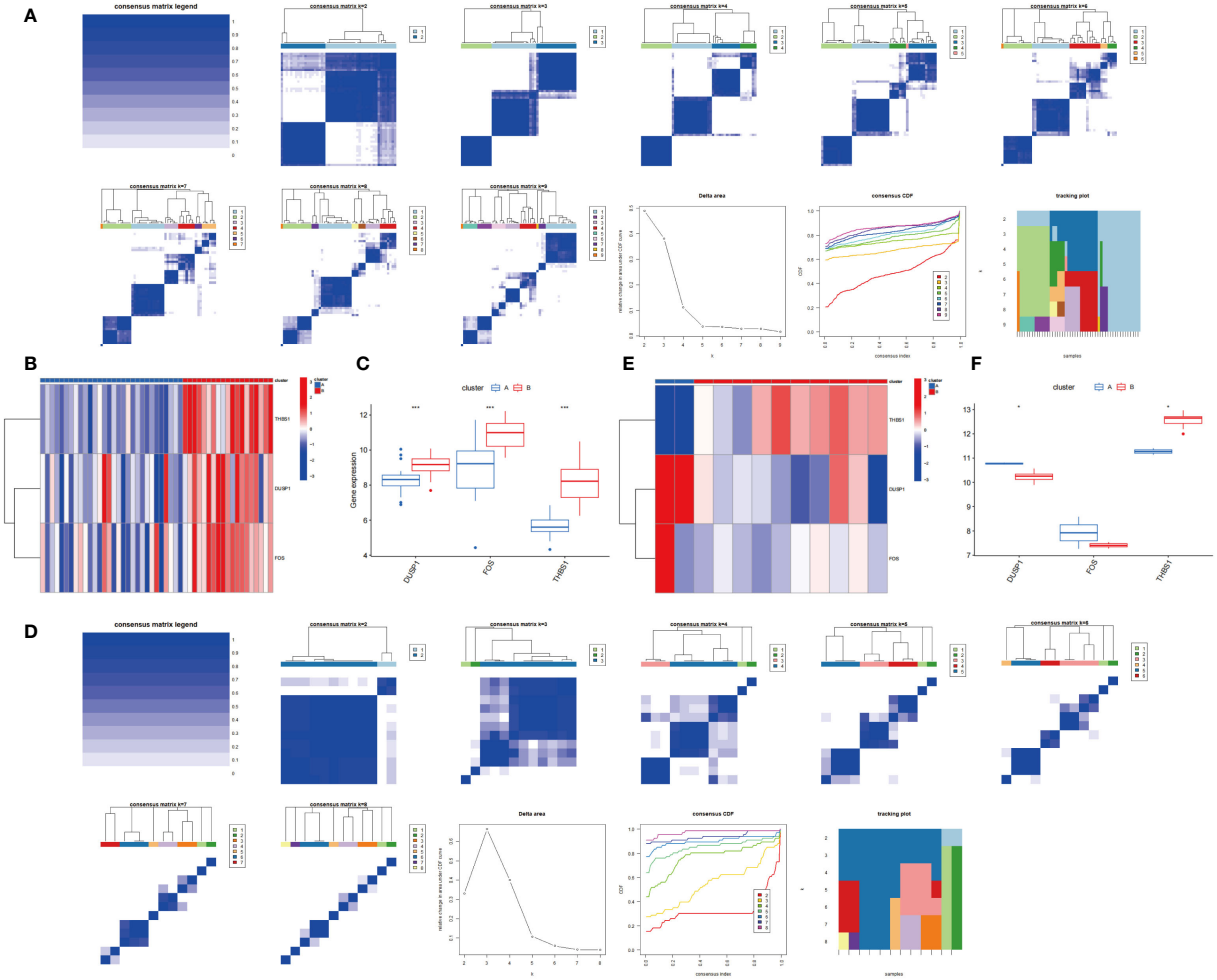
Consensus clustering based on 3 hub genes. **(A)** Consensus matrix and Consensus CDF when k=2-9 in GSE66360. Relative alterations in the area under the CDF curve. Tracking Plot displays sample changes under different k conditions, smaller sample changes indicate better subtype stability. **(B, C)** Expression of 3 hub genes in cluster A and cluster B via heatmap and boxplot in GSE66360. **(D)** Consensus matrix and Consensus CDF when k=2-8 in GSE75181. **(E, F)** Expression of 3 hub genes in cluster A and cluster B via heatmap and boxplot in GSE75181.

As shown in **Figure 14A**, the optimal number of subtypes was 2 in GSE66360, including cluster A and cluster B, which was determined using a consensus matrix plot and a CDF plot. Then, we found that the expression of DUSP1, FOS, and THBS1 in cluster B was higher than that in cluster A (**Figures 14B, C**). For GSE75181, it can also be divided into two stable potential subtypes based on 3 key genes (**Figure 14D**). The expression level of DUSP1 was low in cluster B, while THBS1 was relatively high in cluster B, and there was no significant difference in FOS between the two subtypes (**Figures 14E, F**).

## 4 Discussion

It is now clear that MI and OA are the major causes of death and disability worldwide, which has aroused great concern (2, 43). Previous studies have recognized the multi-aspect association between MI and OA, including shared risk factors, medication use, and perioperative adverse events, though the majority of them are observational in nature (6, 15, 44, 45). On the contrary hand, a recent Mendelian randomization study indicated that the increased odds of MI are likely to have a protective effect against OA (46). Another research also demonstrates that higher levels of serum calcium are associated with decreased risk for OA but increased risk for MI (47). These findings make the relationship between MI and OA appear to remain up for debate, but objective restraints like analytical techniques and sample representativeness should be taken into account. In fact, there are valid reasons to investigate the comorbidity since some relatively high-quality evidence continue to have strong opinions of the tight association between these two diseases (48, 49). Almost all kinds of arthritis are associated with an elevated risk of MI. Risk of MI in people with OA is up to 30% compared with the normal population, which may be related to systemic inflammation and the promotion of cardiovascular risk factors (5). Although the use of nonsteroidal anti-inflammatory medications can substantially relieve discomfort, the risk of MI often increases with the increase in dosage. A combination of low-dose pharmaceuticals or multimodal pain management may delay the onset of MI (50). In addition, MI is the leading cause of perioperative death in patients with OA, especially in patients with poor surgical tolerance, and prompt intervention is needed to avoid fatal cardiovascular events (51). Thus, it is necessary to explore the complex mechanism of this comorbidity. A holistic approach to research in systems biology allows for the explanation of the relationships and underlying principles of complex disorders, which provides us with a strategy to investigate the potential genetic changes and common pathogenesis between MI and OA. In this study, 3 promising diagnostic biomarkers and their regulatory relationships with TFs and immune cells are identified from the perspectives of genomics and immunology, which may provide novel targets for diagnosis and treatment.

In the MI and OA datasets, DUSP1, FOS, and THBS1 were all shown to have significantly different levels of expression, and they were also identified in three public disease databases as associated genes with MI and OA. By integrating multiple machine learning approaches to screen the best candidate genes (**Figures 2**-**5**), DUSP1, FOS, and THBS1 were identified as shared diagnostic markers for this comorbidity. Furthermore, all hub genes demonstrated a strong diagnostic value and superior diagnostic effectiveness (AUC > 0.7) (**Figure 6**). Notably, the single-cell sequencing results are entirely consistent with our bioinformatics analysis (**Figures 7**, **8**). As RT-qPCR results showed, all three genes were significantly differentially expressed between the disease group and the control group, which was almost consistent with the results of our analysis (**Figure 9**). As a member of the bispecific protein phosphatase family, DUSP1 is a phosphatase expressed in various tissues and organs, which is confirmed to be low expressed in the myocardium of the infarcted region. The loss of DUSP1 can affect myocardial energy metabolism by promoting activated mitophagy and apoptosis (52). Interestingly, DUSP1 is downregulated in vascular endothelium under shear stress and acts as a protective factor during the early inflammatory phase of atherosclerosis (53). Another study demonstrates that DUSP1 protects the ischemic myocardium, potentially by increasing its expression to reduce inflammation and apoptosis (54). Study *in vitro* also confirmed that the DUSP1 expression level was significantly lower in cardiomyocytes of neonatal rats under hypoxia and nutrient deprivation. Hence, although this result is not consistent with the dataset, we believe that DUSP1 is a potential diagnostic marker based on previous studies. In addition, OA synovial tissues exhibit reduced DUSP1 expression relative to healthy controls, and DUSP1 is possible to slow the progression of OA through inhibiting the MAPK pathway (55). Zeel T is an effective drug for OA, the positive therapeutic impact has been demonstrated that may be due in large part to the considerable up-regulation of DUSP1 (56). Both *in vivo* and *in vitro* experiments showed that knockdown of DUSP1 aggravated OA cartilage erosion by promoting osteoclast differentiation (57). FOS is a crucial cell proliferation and differentiation regulator that contributes to myocardial fibrosis in post-MI rats (58), which is the main cause of later deterioration of cardiac function. In the male SD rat model of MI with left coronary artery ligation, immunohistochemical results showed that FOS was significantly overexpressed (59). However, targeted FOS therapy has been shown to have the ability to improve cardiac function by ameliorating cardiac interstitial fibrosis after MI (58). According to an intriguing study, the transcript levels of FOS are the best indicators of the relationship between smoking and MI, offering a possible marker for gauging the severity and progression of atherosclerosis (60). On the other hand, cartilage tissues from clinical OA patients and healthy controls were collected and cultured *in vitro*, and the FOS expression level was found to be lower in the disease group (61), which is consistent with our experimental results and dataset. However, another experimental study suggests that FOS is activated and upregulated in promoting OA pathogenesis (62). The function of FOS promoter SNPs is a viable marker in OA progression and joint destruction, although it may need to be tested with larger samples (63). Thus, further studies are needed to investigate the effect of FOS on OA. Besides, the expression of THBS1 is relatively low in the acute phase of MI, but it is secreted by fibroblasts in large amounts one week after infarction, which turns over violently and inhibits neovascularization (64). After MI, tissue launches a repair reaction to rescue heart function. THBS1 produces a protective barrier by highly selective up-regulation in the infarct border zone, hence decreasing inflammatory response and excessive remodeling (65). However, the early stage of MI is dominated by the mass death of cardiomyocytes and THBS1 deposition has been observed around the infarced myocardium over time (66). The ischemic and hypoxic cardiomyocytes may produce THBS1 and efflux, which may increase the concentration of THBS1 in the circulation (67). This may explain the reduced expression of THBS1 in our investigations in vitro. In OA, THBS1 can slow the progression of OA by decreasing angiogenesis and inflammation (68). The expression of THBS1 was much lower in the synovial tissues of OA patients than in the normal synovial tissues, and the down-regulation of THBS1 was more noticeable in the presence of IL-1β (69). Interestingly, the expression of THBS1 is varied in different portions of OA cartilage. The expression of THBS1 is generally lower in the more seriously injured areas, it may be associated with vascular invasion throughout the course of OA (70). A recent study indicated that THBS1 released by chondrocytes may have anti-inflammatory effects on T cells, which may explain THBS1 as a potential protective factor for OA (71). Taken together, 3 shared biomarkers may provide new insights into the diagnosis and management of MI combined with OA.

The increase of lipid oxidation and arterial stiffness by higher levels of inflammatory mediators in peripheral blood, which is mostly reflected in the link between MI and OA, suggests that systemic inflammation may be involved (72). Our study went a step further. The results of GO analysis showed that inflammatory response, oxidative stress response regulation, Immune cell chemotaxis, MAPK pathway-related kinase activity, and cytokine activity were mainly enriched. This indicated that not only inflammation but multiple molecular pathways may be involved in the mechanisms of MI and OA. It was striking that MAPK signaling pathway was almost at the top of KEGG pathway enrichment analysis. IL-17 signaling pathway and TNF signaling pathway were also quite strongly enriched (**Figures 4**, **5**). Therefore, MAPK signaling pathway may play a key role in both disorders. As we all know, MAPK signaling pathway consists of three levels of kinases including MAPK, MAPK kinase and MAPK kinase of kinase, which can activate or interact with each other to complete the signal transduction process. The functions mediated by the different branches are jointly regulating a variety of physiological and pathological effects such as inflammation, immune response, and apoptosis (73). The activation of MAPK signaling pathway is associated with systemic inflammation and has a considerable degree of plasticity (74). Systemic inflammatory stimulation can increase OA progression through MAPK signaling pathway to enhance inflammatory response and decrease cartilage growth (75). Up-regulation of MAPK signaling has also been reported to contribute to the growth of the necrotic core of plaques, which is the most prevalent cause of MI (76). Therefore, MAPK signaling pathway may help elucidate the pathological link of this comorbidity in terms of systemic inflammation. It has been shown that MAPK signaling pathway can be induced by pro-inflammatory factors to trigger increased inflammation, but adverse remodeling usually occurs as a consequence of an overactive inflammatory response after MI (77). In addition, MAPK signaling pathway is implicated in platelet activation and aggregation, and selective inhibition of this pathway can promote microcirculation (78). Myocardial fibrosis can reduce cardiac compliance and adversely affect systolic and diastolic function, which raises the risk of eventual cardiac insufficiency. Fibrosis will be significantly reduced through MAPK signaling pathway inhibition (79). Studies have also clearly demonstrated that MAPK signaling pathway has a role in the pathology of OA, especially in inflammation and apoptosis (80). As previously indicated, the significance of inflammation in the development and progression of OA is gradually becoming recognized and accepted. Inflammatory mediators disrupt the balance between consolidation and fracture in the joint. Inhibiting the activation of MAPK signaling pathway can reduce the further bone destruction induced by inflammatory mediators, and directly improve the downstream inflammatory effect and chondrocyte apoptosis (81). Another study confirms that MAPK signaling pathway is associated with bone loss while blocking the process can have good therapeutic effects (82). Furthermore, mechanical stress can cause cartilage disintegration as a physical stimulus by activating the MAPK signaling pathway (83). IL-17 signaling pathway and TNF signaling pathway are also related to the inflammatory response, which suggests that our research results are consistent with previous studies. These pathways above have the great potential to help reveal the shared mechanisms between the comorbidity.

To further explore the immune microenvironment of these two diseases, immune infiltration analysis in this study illustrated that many types of immune cells were closely associated, both innate immunity and adaptive immunity participated in MI and OA (**Figures 11**, **12**). Innate immunity can quickly recognize and respond to the sterile inflammatory state during MI, and then recruit circulating monocytes and lymphocytes to drive adaptive immunity. However, the radical immune response may be a risky trigger for exacerbating myocardial injury (84). As in other organs, macrophages are major participants in the innate immune response in the heart (85). Macrophages infiltrate and expand in the area of MI, showing a pro-inflammatory effect in the early stage, but in the repair stage representing an anti-inflammatory state (86). Neutrophils are important participants in the early inflammatory process of MI, with large numbers of neutrophils entering the heart, they will play a surveillance function as well as an antiinflammatory role (87). Besides, NK cells and monocytes may collaborate to accelerate the development of inflammation (88). T cells and B cells constitute a major part of adaptive immunity. The role of adaptive immunity in MI remains uncertain since different T cell subtypes can play different roles such as injury or repair (89). B cells are thought to protect myocardial cell survival and promote cardiac repair in the early stage of MI (90). The immune response is also a participant in OA. T cells have been clearly observed to dominate infiltration in the synovium of OA patients and may be involved in inflammation and cartilage destruction (91). Activated B cells are infiltrated in OA, which associated with inflammation (92). Furthermore, innate immunity has been reported to play a synergistic role in the sterile inflammatory process of OA. It can promote OA progression through a variety of innate immune response cells and inflammatory factors (93), which differs from our analysis. Additionally, it is worth noting that plasma cells, T cells follicular helper, and T cells CD4 memory resting are all significantly associated with MI and OA, but show completely opposite trends, which is well worth further investigation. Therefore, these analyses may provide a direction for further understanding the immunological link between the two diseases.

We also identified a total of 18 transcription factors that could regulate the hub genes. FOXC1, which was simultaneously regulating all of the hub genes, may be the crucial TF (**Figure 10**). There is evidence that FOXC1 can promote angiogenesis in the ischemic injury area, and prevent the subsequent decline in cardiac function by reducing myocardial fibrosis (94). Hence, it is expected to become a therapeutic target for assisting myocardial repair. Nevertheless, high expression of FOXC1 could promote inflammatory response and aggravate bone degradation in OA (95). This effect is confirmed to be reversed in another study by downregulation of FOXC1 (96). Our findings are consistent with the results of previous studies. The expression level of FOXC1 was suppressed in MI but significantly promoted in OA. It is intriguing that our results revealed that FOXC1 can regulate MI and OA concurrently at the transcriptional level, and there is currently no literature indicating the mode of action in this comorbidity. Notably, FOXC1 was predicted to have many binding sites with 3 hub genes, which provided directions for further elucidating the regulatory relationship. Considering the extensive expression of TFs in humans, further exploration of specific links is well worth pursuing.

Although the comorbidity of MI and OA has been thoroughly discussed, the lack of understanding of the molecular process has put us in a conundrum. Here, we aimed to investigate the underlying common molecular mechanisms by examining microarray data of MI/OA patients and healthy controls. DUSP1, FOS, and THBS1 were found by rigorous screening out by several systems biology approaches and machine learning. The diagnostic utility with high sensitivity and specificity was confirmed in external datasets. MAPK signaling pathway was identified by two independent enrichment analyses, which may assist to identify the pathogenic link between them in the future. Finally, the immune cell subtypes and upstream regulators governed by hub genes were also studied in depth, and the sequenced tissue samples were stably divided into two subtypes based on the hub gene expression (**Figures 13**, **14**). To sum up, our findings provide new insights into the molecular mechanisms underlying the comorbidity of MI and OA, which may provide a rationale for developing precision medicine.

## 5 Limitation

Our study exists some limitations. Firstly, despite the combination of public disease databases, the sample size of datasets used for bioinformatics analysis was still limited. Different types of samples may have caused differences. Secondly, our analysis was only performed at the expression level of the genes, further experiments *in vivo* or *in vitro* were needed to validate our results. Thirdly, the exact regulatory mechanism of immune cells and TFs corresponding to the hub genes were only predicted in the online database. Therefore, more efforts are necessary to explore the concrete molecular mechanisms between MI and OA in the future.

## 6 Conclusion

In conclusion, by integrated systems biology analysis this research firstly revealed the common underlying signal pathway for MI and OA, and 3 hub genes (DUSP1, FOS, and THBS1) were identified as novel shared biomarkers, which may be potential therapeutic targets. Additionally, multiple immune cells and TFs corresponding to the three hub genes were analyzed to illustrate the regulatory mechanism. Our study provides new sights into shared molecular mechanisms between MI and OA.

## Data availability statement

The datasets presented in this study can be found in online repositories. The names of the repository/repositories and accession number(s) can be found in the article/**Supplementary Material**.

## Ethics statement

All animal experimental procedures were in accordance with the institutional and international guidelines and approved by the Ethics Committee of the First Affiliated Hospital of Guangzhou University of Chinese Medicine (GZTCMF1-20240009).

## Author contributions

YLu: Data curation, Formal analysis, Software, Validation, Writing – original draft. YLi: Data curation, Resources, Validation, Visualization, Writing – original draft. WX: Formal analysis, Investigation, Software, Writing – original draft. WH: Data curation, Formal analysis, Visualization, Writing – original draft. DL: Conceptualization, Investigation, Methodology, Supervision, Writing – review & editing. HZ: Conceptualization, Investigation, Methodology, Project administration, Writing – review & editing.
