# Supplementary material for: Systems biology-based analysis exploring shared biomarkers and pathogenesis of myocardial infarction combined with osteoarthritis
Source: Front Immunol. 2024 Jul 17;15:1398990. doi: 10.3389/fimmu.2024.1398990 (PMC11288954; doi:10.3389/fimmu.2024.1398990)
Supplement: Supplementary file 1 [file DataSheet_1.docx]

Supplementary Material

**Systems biology-based analysis exploring** **shared** **biomarkers and pathogenesis of myocardial infarction combined with osteoarthritis**

**Yuan Luo^1^, Huanyi Zhao^2^, Weiqi Xue^1^, Weifeng He^1^, Di Lv^3*^, Yongrui Liu^2*^**

^1^Guangzhou University of Chinese Medicine, Guangzhou, Guangdong, 510405, China

^2^The First Affiliated Hospital of Guangzhou University of Chinese Medicine, Guangzhou, Guangdong, 510405, China

^3^Taizhou Hospital of Traditional Chinese Medicine, Taizhou, Jiangsu, 225300, China

*** Correspondence:**Di Lv
lvdigood@163.com

Yongrui Liu
liuyongrui0531@163.com

# Supplementary Figures


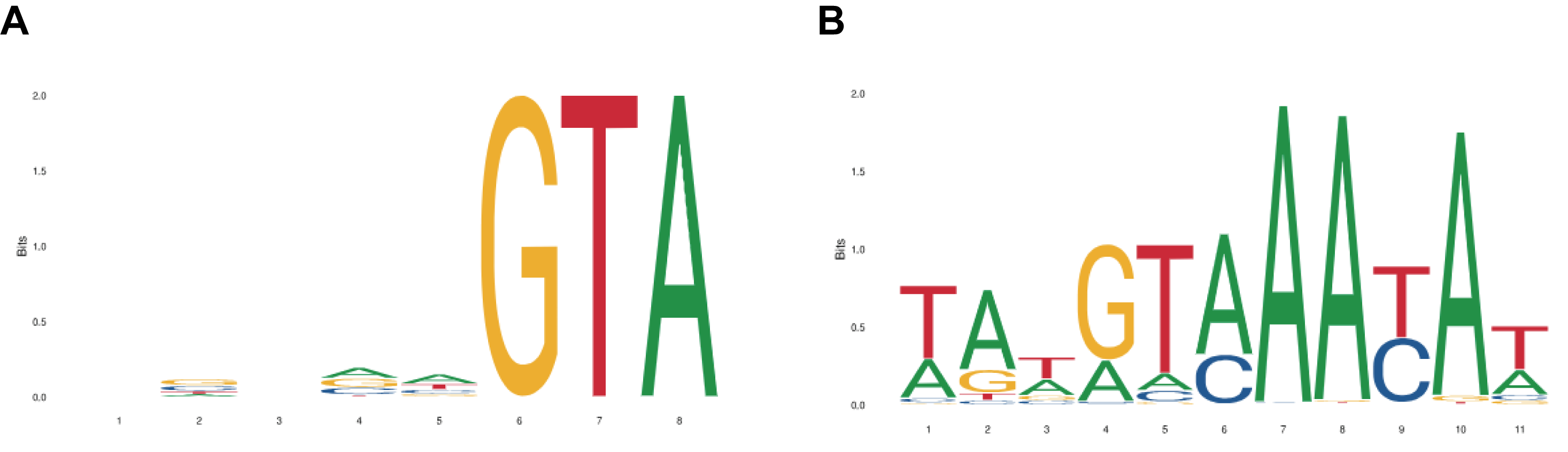


**FIGURE S1** Transcription factor FOXC1’s binding regions. **(A)** MA0032.1.FOXC1. **(B)** MA0032.2.FOXC1.


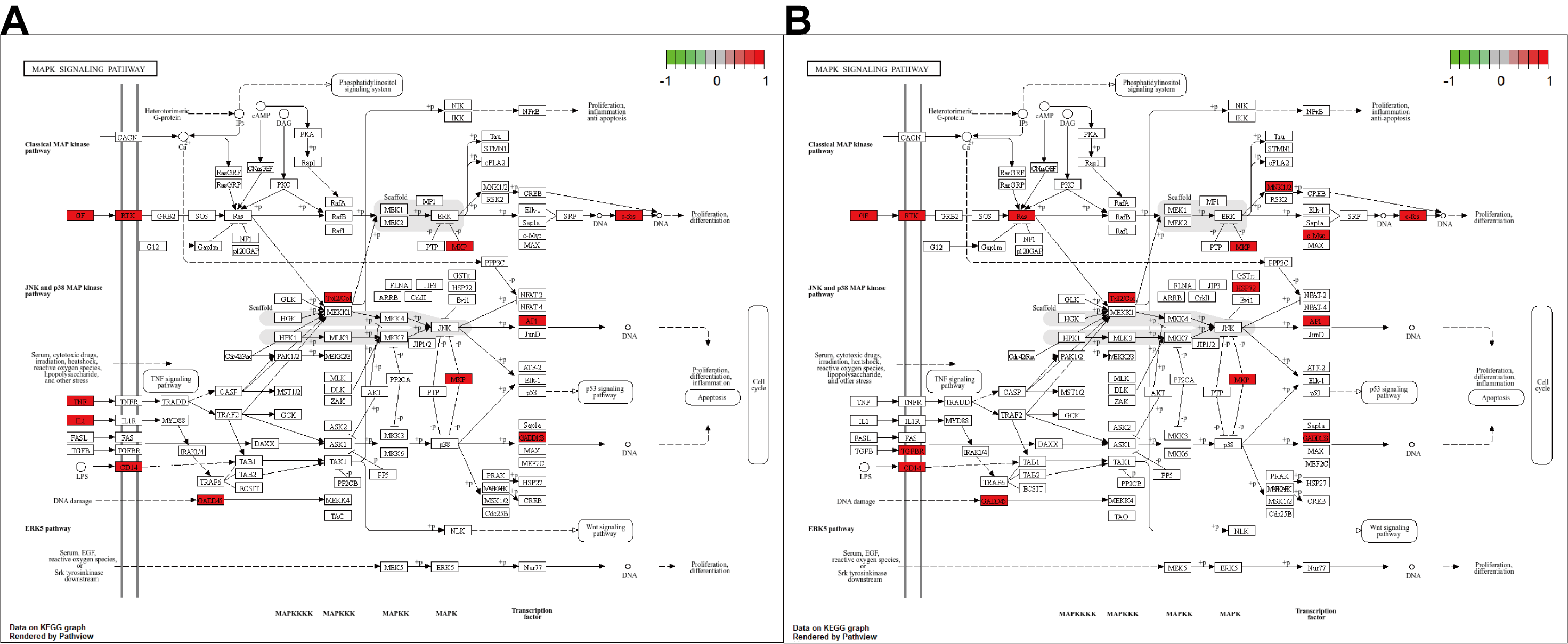


**FIGURE S2** Annotation of DEGs in the MAPK signaling pathway with red rectangle representing positive enrichment factor. **(A)** DEGs of MI (GSE66360) in the MAPK signaling pathway. **(B)** DEGs of OA (GSE75181) in the MAPK signaling pathway.

# Supplementary Tables

TABLE S1 GO enrichment analysis of DEGs (top 5).

| **Ontology** | **ID** | **Description** | **P-value** | **Count** | **Gene ID** |
| --- | --- | --- | --- | --- | --- |
| BP | GO:0009612 | response to mechanical stimulus | 1.88E-07 | 6 | FOS/GADD45A/JUN/FOSB/THBS1/PTGS2 |
| BP | GO:0046683 | response to organophosphorus | 4.72E-07 | 5 | FOS/JUN/FOSB/DUSP1/PTGS2 |
| BP | GO:0045936 | negative regulation of phosphate metabolic process | 6.78E-07 | 7 | PPP1R15A/TRIB1/GADD45A/GADD45B/DUSP1/DUSP6/RGS2 |
| BP | GO:0010563 | negative regulation of phosphorus metabolic process | 6.89E-07 | 7 | PPP1R15A/TRIB1/GADD45A/GADD45B/DUSP1/DUSP6/RGS2 |
| BP | GO:0014074 | response to purine-containing compound | 8.65E-07 | 5 | FOS/JUN/FOSB/DUSP1/PTGS2 |
| CC | GO:0090575 | RNA polymerase II transcription regulator complex | 0.000972789 | 3 | DDIT3/FOS/JUN |
| CC | GO:0005667 | transcription regulator complex | 0.001264066 | 4 | DDIT3/NR4A2/FOS/JUN |
| CC | GO:0000164 | protein phosphatase type 1 complex | 0.017508687 | 1 | PPP1R15A |
| CC | GO:0032993 | protein-DNA complex | 0.022666771 | 2 | DDIT3/FOS |
| CC | GO:0043073 | germ cell nucleus | 0.023279471 | 1 | MARCKS |
| MF | GO:0001228 | DNA-binding transcription activator activity, RNA polymerase II-specific | 4.33E-08 | 8 | DDIT3/NR4A2/FOS/JUN/FOSB/RLF/CEBPD/EGR1 |
| MF | GO:0001216 | DNA-binding transcription activator activity | 4.80E-08 | 8 | DDIT3/NR4A2/FOS/JUN/FOSB/RLF/CEBPD/EGR1 |
| MF | GO:0008330 | protein tyrosine/threonine phosphatase activity | 6.71E-05 | 2 | DUSP1/DUSP6 |
| MF | GO:0017017 | MAP kinase tyrosine/serine/threonine phosphatase activity | 0.000116012 | 2 | DUSP1/DUSP6 |
| MF | GO:0033549 | MAP kinase phosphatase activity | 0.000226697 | 2 | DUSP1/DUSP6 |

TABLE S2 KEGG pathways analysis of DEGs (dual-positive).

| **ID** | **Description** | **pvalue** | **p.adjust** | **qvalue** | **geneID** |
| --- | --- | --- | --- | --- | --- |
| hsa04010 | MAPK signaling pathway | 7.86E-08 | 8.73E-06 | 4.30E-06 | DDIT3/FOS/GADD45A/JUN/GADD45B/DUSP1/DUSP6/MAP3K8 |
| hsa04657 | IL-17 signaling pathway | 4.36E-05 | 0.001209125 | 0.000596249 | FOS/JUN/FOSB/PTGS2 |
| hsa04668 | TNF signaling pathway | 8.66E-05 | 0.001601697 | 0.000789836 | FOS/JUN/PTGS2/MAP3K8 |
| hsa04115 | p53 signaling pathway | 0.00049971 | 0.005546779 | 0.002735254 | GADD45A/GADD45B/THBS1 |
| hsa04912 | GnRH signaling pathway | 0.001014381 | 0.008042591 | 0.003966 | HBEGF/JUN/EGR1 |
| hsa04064 | NF-kappa B signaling pathway | 0.001402187 | 0.008646822 | 0.004263962 | GADD45A/GADD45B/PTGS2 |
| hsa04620 | Toll-like receptor signaling pathway | 0.001402187 | 0.008646822 | 0.004263962 | FOS/JUN/MAP3K8 |
| hsa04660 | T cell receptor signaling pathway | 0.001402187 | 0.008646822 | 0.004263962 | FOS/JUN/MAP3K8 |
| hsa04915 | Estrogen signaling pathway | 0.003149325 | 0.01621802 | 0.007997506 | HBEGF/FOS/JUN |
| hsa04662 | B cell receptor signaling pathway | 0.01358651 | 0.037702566 | 0.018592067 | FOS/JUN |
| hsa04012 | ErbB signaling pathway | 0.014548685 | 0.039387903 | 0.019423148 | HBEGF/JUN |
| hsa04933 | AGE-RAGE signaling pathway in diabetic complications | 0.019785598 | 0.047743509 | 0.023543504 | JUN/EGR1 |
| hsa04625 | C-type lectin receptor signaling pathway | 0.021298215 | 0.049252122 | 0.024287438 | JUN/PTGS2 |
| hsa04926 | Relaxin signaling pathway | 0.031789275 | 0.066577539 | 0.032831029 | FOS/JUN |
| hsa04068 | FoxO signaling pathway | 0.032702242 | 0.067221275 | 0.033148471 | GADD45A/GADD45B |

TABLE S3 Degree valves of the core genes in PPI.

| **Symbol** | **Degree** | **Symbol** | **Degree** | **Symbol** | **Degree** | **Symbol** | **Degree** | **Symbol** | **Degree** |
| --- | --- | --- | --- | --- | --- | --- | --- | --- | --- |
| STAT3 | 77 | FOXO3 | 27 | ITGAM | 19 | IFNG | 14 | AGTR2 | 9 |
| MAPK3 | 67 | SP1 | 27 | CCND1 | 19 | NOS2 | 13 | ADAM17 | 9 |
| MAPK1 | 64 | NR3C1 | 26 | CCL22 | 19 | E2F1 | 13 | CALR | 9 |
| TP53 | 61 | TLR4 | 26 | NFKBIA | 19 | CD40 | 13 | ADRB2 | 9 |
| PIK3CA | 60 | FOXO1 | 26 | TIMP1 | 19 | BCL2 | 13 | BSG | 9 |
| AKT1 | 51 | IL18 | 26 | HMGB1 | 18 | MAPK10 | 13 | AGER | 9 |
| IL6 | 50 | CD44 | 26 | MDM2 | 18 | SMAD1 | 13 | CAT | 9 |
| CTNNB1 | 48 | CASP8 | 26 | HSPA4 | 18 | TGFBR2 | 13 | PIK3CG | 9 |
| HSP90AA1 | 48 | CCL2 | 26 | TLR2 | 18 | FGFR3 | 13 | VIM | 9 |
| EGFR | 45 | AR | 25 | CSF3 | 18 | IL6ST | 13 | LGALS3 | 9 |
| PTPN11 | 44 | CREB1 | 25 | CXCL2 | 18 | PRKAR1A | 12 | CASP1 | 9 |
| VEGFA | 44 | IL4 | 25 | IL17F | 18 | SOD1 | 12 | NLRP3 | 9 |
| IL10 | 43 | MMP1 | 25 | SAA1 | 18 | MAPT | 12 | CASP7 | 9 |
| TNF | 43 | CXCL8 | 25 | THBS1 | 17 | HSPA1A | 12 | CCR2 | 9 |
| ITGB1 | 43 | EDN1 | 24 | PTPN1 | 17 | YAP1 | 12 | CXCR3 | 9 |
| NFKB1 | 42 | CASP3 | 24 | CCL20 | 17 | CDKN2A | 12 | ICAM1 | 9 |
| FN1 | 41 | SDC4 | 24 | PTK2B | 17 | CDK1 | 12 | SERPINC1 | 9 |
| ESR1 | 39 | IL1A | 24 | LCN2 | 17 | TGFBR1 | 12 | SPP1 | 9 |
| FOS | 39 | CXCL10 | 24 | APOA1 | 16 | IL6R | 12 | CTGF | 9 |
| MAPK14 | 38 | CSF2 | 24 | LEP | 16 | SERPINE1 | 11 | IGFBP3 | 9 |
| JAK2 | 38 | CDKN1A | 23 | CCR5 | 16 | MAP3K5 | 11 | FLT1 | 9 |
| ITGB3 | 38 | PPARA | 22 | NGF | 16 | ANGPT1 | 11 | FGF23 | 9 |
| IL1B | 37 | PPARGC1A | 22 | BDNF | 15 | TEK | 11 | KNG1 | 8 |
| RAC1 | 36 | KDR | 22 | CCL11 | 15 | TGFB2 | 11 | S100B | 8 |
| MMP2 | 35 | SIRT1 | 22 | IL2RB | 15 | MMP7 | 11 | SOD2 | 8 |
| MAPK8 | 35 | MMP3 | 22 | BCL2L1 | 15 | COL1A1 | 11 | ALOX5 | 8 |
| SMAD3 | 34 | IL17A | 22 | SOCS3 | 15 | FGFR1 | 11 | PRKAB1 | 8 |
| TGFB1 | 33 | PPARG | 21 | MTOR | 15 | ITGA2 | 11 | PRKAA1 | 8 |
| FGF2 | 33 | MYD88 | 21 | EZH2 | 15 | PLAU | 11 | BGN | 8 |
| PTK2 | 33 | MAP2K1 | 21 | PLAUR | 15 | APOE | 10 | CD14 | 8 |
| LIF | 33 | CXCL1 | 21 | HSPA5 | 14 | CDH2 | 10 | VWF | 8 |
| HIF1A | 32 | SMAD2 | 21 | NCOR2 | 14 | TNFRSF1B | 10 | TNFSF10 | 8 |
| CXCR4 | 31 | DCN | 21 | PIK3CB | 14 | CCR3 | 10 | LPAR3 | 8 |
| CXCL12 | 30 | LRP1 | 20 | BECN1 | 14 | ITGA6 | 10 | PARP1 | 8 |
| HGF | 30 | APP | 20 | PTEN | 14 | PTGS2 | 10 | TXN | 8 |
| SDC1 | 29 | CHUK | 20 | TNFRSF1A | 14 | FAS | 10 | PCNA | 8 |
| MMP9 | 29 | IGF1 | 20 | COL1A2 | 14 | COL3A1 | 10 | EGR1 | 8 |
| SMAD4 | 28 | MET | 20 | ENG | 14 | CXCR2 | 10 | SOCS1 | 8 |
| HDAC1 | 28 | OSM | 20 | VCAM1 | 14 | DNMT1 | 10 | LEPR | 8 |
| CCL5 | 28 | ITGB2 | 19 | TGFB3 | 14 | DUSP1 | 10 |  |  |

TABLE S4 GO enrichment analysis of the core genes in PPI (top 10).

| **ONTOLOGY** | **ID** | **Description** | **P-value** | **Count** | **Gene ID** |
| --- | --- | --- | --- | --- | --- |
| BP | GO:0019221 | cytokine-mediated signaling pathway | 1.47E-49 | 62 | STAT3/MAPK3/TP53/AKT1/IL6/PTPN11/TNF/JAK2/IL1B/HIF1A/CXCR4/CXCL12/SMAD4/CCL5/FOXO3/IL18/CD44/CASP8/CCL2/CXCL8/EDN1/IL1A/CXCL10/CSF2/SIRT1/IL17A/PPARG/MYD88/CXCL1/CHUK/OSM/CCL22/NFKBIA/CSF3/CXCL2/IL17F/PTPN1/CCL20/PTK2B/APOA1/LEP/CCR5/CCL11/IL2RB/TNFRSF1A/IFNG/IL6ST/HSPA1A/YAP1/IL6R/ANGPT1/TNFRSF1B/CCR3/FAS/CXCR2/ADAM17/CASP1/CCR2/CXCR3/EGR1/SOCS1/LEPR |
| BP | GO:0050900 | leukocyte migration | 8.83E-49 | 57 | MAPK3/MAPK1/AKT1/IL6/VEGFA/IL10/TNF/ITGB1/ITGB3/IL1B/RAC1/MMP2/PTK2/CXCR4/CXCL12/MMP9/CCL5/CCL2/IL4/CXCL8/EDN1/IL1A/CXCL10/IL17A/MYD88/CXCL1/APP/ITGB2/CCL22/HMGB1/CXCL2/SAA1/THBS1/CCL20/PTK2B/LEP/CCR5/CCL11/VCAM1/IL6R/SERPINE1/TGFB2/ITGA2/ITGA6/CXCR2/DUSP1/ADAM17/CALR/BSG/AGER/PIK3CG/LGALS3/CCR2/CXCR3/ICAM1/FLT1/ALOX5 |
| BP | GO:0042060 | wound healing | 7.40E-45 | 56 | PIK3CA/IL6/VEGFA/TNF/ITGB1/FN1/MAPK14/JAK2/ITGB3/SMAD3/TGFB1/FGF2/PTK2/HIF1A/CXCR4/SDC1/SMAD4/TLR4/CD44/EDN1/CASP3/SDC4/IL1A/CDKN1A/PPARA/KDR/PPARG/SMAD2/IGF1/TIMP1/SAA1/THBS1/MTOR/PLAUR/PIK3CB/PTEN/TGFB3/CD40/TGFBR2/IL6ST/YAP1/TGFBR1/SERPINE1/MAP3K5/TGFB2/COL1A1/ITGA2/PLAU/APOE/COL3A1/ADAM17/PIK3CG/SERPINC1/KNG1/ALOX5/VWF |
| BP | GO:0043410 | positive regulation of MAPK cascade | 2.83E-43 | 57 | MAPK3/IL6/CTNNB1/EGFR/PTPN11/VEGFA/TNF/JAK2/ITGB3/IL1B/TGFB1/FGF2/LIF/HGF/CCL5/TLR4/CD44/CCL2/AR/EDN1/IL1A/KDR/MYD88/MAP2K1/APP/IGF1/OSM/CCL22/HMGB1/THBS1/PTPN1/CCL20/PTK2B/LEP/CCL11/EZH2/PTEN/TGFB3/CD40/FGFR3/SOD1/TGFBR1/MAP3K5/ANGPT1/TEK/TGFB2/FGFR1/APOE/CDH2/ADRB2/AGER/PIK3CG/ICAM1/IGFBP3/FLT1/FGF23/LPAR3 |
| BP | GO:0060326 | cell chemotaxis | 3.08E-43 | 49 | MAPK3/MAPK1/IL6/VEGFA/IL10/IL1B/RAC1/MMP2/FGF2/PTK2/CXCR4/CXCL12/HGF/CCL5/CCL2/IL4/CXCL8/EDN1/CXCL10/KDR/CXCL1/MET/ITGB2/CCL22/HMGB1/CXCL2/SAA1/THBS1/CCL20/PTK2B/CCR5/CCL11/VCAM1/IL6R/SERPINE1/TGFB2/FGFR1/CCR3/CXCR2/DUSP1/ADAM17/CALR/BSG/PIK3CG/LGALS3/CCR2/CXCR3/FLT1/ALOX5 |
| BP | GO:0033002 | muscle cell proliferation | 4.06E-43 | 45 | PIK3CA/AKT1/IL6/CTNNB1/IL10/TNF/FOS/MAPK14/JAK2/ITGB3/MMP2/TGFB1/FGF2/HGF/MMP9/HDAC1/CCL5/TLR4/IL18/EDN1/CDKN1A/PPARGC1A/PPARG/MYD88/IGF1/THBS1/PTEN/TGFB3/IFNG/SMAD1/TGFBR2/PRKAR1A/YAP1/CDK1/TGFBR1/IL6R/MAP3K5/ANGPT1/TGFB2/ITGA2/APOE/PTGS2/DNMT1/IGFBP3/SOD2 |
| BP | GO:0062197 | cellular response to chemical stress | 6.33E-39 | 47 | MAPK3/MAPK1/TP53/PIK3CA/AKT1/IL6/CTNNB1/EGFR/IL10/TNF/FOS/JAK2/MMP2/MAPK8/HIF1A/HGF/MMP9/FOXO3/TLR4/FOXO1/EDN1/CASP3/PPARGC1A/SIRT1/MMP3/CHUK/MET/MTOR/EZH2/BECN1/BCL2/SOD1/MAPT/HSPA1A/CDK1/MAP3K5/PTGS2/FAS/CAT/CASP1/NLRP3/SOD2/ALOX5/PRKAA1/PARP1/TXN/PCNA |
| BP | GO:0070482 | response to oxygen levels | 4.04E-38 | 46 | TP53/AKT1/VEGFA/TNF/FOS/MMP2/SMAD3/HIF1A/CXCR4/CXCL12/SMAD4/FOXO3/FOXO1/EDN1/CASP3/IL1A/PPARA/SIRT1/PPARG/MDM2/TLR2/THBS1/PTK2B/LEP/MTOR/PIK3CB/BECN1/PTEN/VCAM1/TGFB3/NOS2/E2F1/BCL2/TGFBR2/TGFB2/COL1A1/ITGA2/PLAU/PTGS2/FAS/ADAM17/AGER/CAT/SOD2/PRKAA1/EGR1 |
| BP | GO:0030595 | leukocyte chemotaxis | 4.89E-38 | 41 | MAPK3/MAPK1/IL6/VEGFA/IL10/IL1B/RAC1/MMP2/PTK2/CXCR4/CXCL12/CCL5/CCL2/IL4/CXCL8/EDN1/CXCL10/CXCL1/ITGB2/CCL22/HMGB1/CXCL2/SAA1/THBS1/CCL20/PTK2B/CCR5/CCL11/IL6R/SERPINE1/TGFB2/CXCR2/DUSP1/ADAM17/CALR/BSG/PIK3CG/LGALS3/CCR2/FLT1/ALOX5 |
| BP | GO:0070997 | neuron death | 1.36E-37 | 47 | TP53/PIK3CA/AKT1/CTNNB1/IL10/TNF/FOS/JAK2/HIF1A/CCL5/FOXO3/NR3C1/TLR4/CASP8/CCL2/CASP3/PPARA/PPARGC1A/KDR/SIRT1/APP/ITGB2/ITGAM/CCND1/CSF3/PTK2B/NGF/BDNF/BCL2L1/HSPA5/TGFB3/IFNG/BCL2/IL6ST/SOD1/MAPT/MAP3K5/ANGPT1/TGFB2/APOE/TNFRSF1B/FAS/AGTR2/CASP7/SOD2/PARP1/EGR1 |
| CC | GO:0045121 | membrane raft | 1.22E-22 | 32 | MAPK3/MAPK1/CTNNB1/EGFR/TNF/ITGB1/JAK2/CASP8/CASP3/SDC4/KDR/APP/ITGB2/ITGAM/TLR2/PTK2B/TNFRSF1A/TGFBR2/IL6ST/PRKAR1A/MAPT/TGFBR1/ANGPT1/TEK/CDH2/TNFRSF1B/PTGS2/FAS/ADAM17/BSG/ICAM1/CD14 |
| CC | GO:0098857 | membrane microdomain | 1.34E-22 | 32 | MAPK3/MAPK1/CTNNB1/EGFR/TNF/ITGB1/JAK2/CASP8/CASP3/SDC4/KDR/APP/ITGB2/ITGAM/TLR2/PTK2B/TNFRSF1A/TGFBR2/IL6ST/PRKAR1A/MAPT/TGFBR1/ANGPT1/TEK/CDH2/TNFRSF1B/PTGS2/FAS/ADAM17/BSG/ICAM1/CD14 |
| CC | GO:0005925 | focal adhesion | 3.07E-19 | 32 | MAPK3/MAPK1/CTNNB1/EGFR/ITGB1/JAK2/ITGB3/RAC1/PTK2/CD44/SDC4/MAP2K1/LRP1/ITGB2/PTK2B/PLAUR/HSPA5/ENG/FGFR3/HSPA1A/TEK/ITGA2/PLAU/CDH2/ITGA6/ADAM17/CALR/BSG/CAT/VIM/ICAM1/FLT1 |
| CC | GO:0009897 | external side of plasma membrane | 4.97E-19 | 33 | TNF/ITGB1/ITGB3/CXCR4/CXCL12/SDC1/TLR4/CXCL10/KDR/IL17A/ITGB2/ITGAM/THBS1/CCR5/IL2RB/ENG/VCAM1/CD40/TGFBR2/IL6ST/IL6R/MAP3K5/ITGA2/CCR3/ITGA6/FAS/CXCR2/CALR/CCR2/CXCR3/ICAM1/CD14/LEPR |
| CC | GO:0030055 | cell-substrate junction | 6.15E-19 | 32 | MAPK3/MAPK1/CTNNB1/EGFR/ITGB1/JAK2/ITGB3/RAC1/PTK2/CD44/SDC4/MAP2K1/LRP1/ITGB2/PTK2B/PLAUR/HSPA5/ENG/FGFR3/HSPA1A/TEK/ITGA2/PLAU/CDH2/ITGA6/ADAM17/CALR/BSG/CAT/VIM/ICAM1/FLT1 |
| CC | GO:0005788 | endoplasmic reticulum lumen | 8.90E-15 | 24 | MAPK3/MAPK1/IL6/FN1/EDN1/APP/TIMP1/THBS1/APOA1/PLAUR/HSPA5/COL1A2/COL1A1/APOE/CDH2/PTGS2/COL3A1/ADAM17/CALR/SERPINC1/SPP1/IGFBP3/FGF23/KNG1 |
| CC | GO:0031983 | vesicle lumen | 2.52E-14 | 24 | MAPK1/HSP90AA1/EGFR/VEGFA/NFKB1/FN1/MAPK14/TGFB1/HGF/CXCL1/APP/IGF1/TIMP1/HMGB1/THBS1/LCN2/APOA1/TGFB3/SERPINE1/TGFB2/CAT/KNG1/ALOX5/VWF |
| CC | GO:0031093 | platelet alpha granule lumen | 1.02E-13 | 13 | VEGFA/FN1/TGFB1/HGF/APP/IGF1/TIMP1/THBS1/TGFB3/SERPINE1/TGFB2/KNG1/VWF |
| CC | GO:0034774 | secretory granule lumen | 1.60E-13 | 23 | MAPK1/HSP90AA1/VEGFA/NFKB1/FN1/MAPK14/TGFB1/HGF/CXCL1/APP/IGF1/TIMP1/HMGB1/THBS1/LCN2/APOA1/TGFB3/SERPINE1/TGFB2/CAT/KNG1/ALOX5/VWF |
| CC | GO:0060205 | cytoplasmic vesicle lumen | 1.95E-13 | 23 | MAPK1/HSP90AA1/VEGFA/NFKB1/FN1/MAPK14/TGFB1/HGF/CXCL1/APP/IGF1/TIMP1/HMGB1/THBS1/LCN2/APOA1/TGFB3/SERPINE1/TGFB2/CAT/KNG1/ALOX5/VWF |
| MF | GO:0005126 | cytokine receptor binding | 3.14E-39 | 44 | IL6/VEGFA/IL10/TNF/JAK2/ITGB3/IL1B/SMAD3/TGFB1/LIF/CXCL12/CCL5/IL18/CASP8/CCL2/IL4/CXCL8/CASP3/IL1A/CXCL10/CSF2/MYD88/CXCL1/SMAD2/OSM/CCL22/CSF3/CXCL2/IL17F/CCL20/NGF/BDNF/CCL11/ENG/TGFB3/IFNG/TGFBR2/IL6ST/TGFBR1/IL6R/TGFB2/ADAM17/CCR2/TNFSF10 |
| MF | GO:0005125 | cytokine activity | 1.53E-28 | 34 | IL6/VEGFA/IL10/TNF/IL1B/TGFB1/FGF2/LIF/CXCL12/CCL5/IL18/CCL2/IL4/CXCL8/EDN1/IL1A/CXCL10/CSF2/IL17A/CXCL1/OSM/CCL22/TIMP1/HMGB1/CSF3/CXCL2/IL17F/CCL20/CCL11/TGFB3/IFNG/TGFB2/SPP1/TNFSF10 |
| MF | GO:0030546 | signaling receptor activator activity | 8.09E-28 | 44 | IL6/EGFR/VEGFA/IL10/TNF/IL1B/TGFB1/FGF2/LIF/CXCL12/HGF/CCL5/IL18/CCL2/IL4/CXCL8/EDN1/IL1A/CXCL10/CSF2/IL17A/CXCL1/APP/IGF1/OSM/CCL22/TIMP1/HMGB1/CSF3/CXCL2/IL17F/CCL20/APOA1/LEP/NGF/BDNF/CCL11/TGFB3/IFNG/TGFB2/LGALS3/SPP1/FGF23/TNFSF10 |
| MF | GO:0048018 | receptor ligand activity | 5.54E-26 | 42 | IL6/VEGFA/IL10/TNF/IL1B/TGFB1/FGF2/LIF/CXCL12/HGF/CCL5/IL18/CCL2/IL4/CXCL8/EDN1/IL1A/CXCL10/CSF2/IL17A/CXCL1/IGF1/OSM/CCL22/TIMP1/HMGB1/CSF3/CXCL2/IL17F/CCL20/APOA1/LEP/NGF/BDNF/CCL11/TGFB3/IFNG/TGFB2/LGALS3/SPP1/FGF23/TNFSF10 |
| MF | GO:0019955 | cytokine binding | 2.37E-19 | 22 | ITGB1/ITGB3/FGF2/CXCR4/HMGB1/IL17F/THBS1/CCR5/IL2RB/TNFRSF1A/ENG/TGFB3/TGFBR2/IL6ST/TGFBR1/IL6R/TNFRSF1B/CCR3/CXCR2/CCR2/CXCR3/LEPR |
| MF | GO:0005178 | integrin binding | 1.41E-18 | 22 | ITGB1/FN1/ITGB3/IL1B/FGF2/PTK2/CXCL12/KDR/IGF1/ITGB2/ITGAM/HMGB1/THBS1/VCAM1/ITGA2/ITGA6/COL3A1/ADAM17/CALR/ICAM1/SPP1/VWF |
| MF | GO:0019902 | phosphatase binding | 1.85E-16 | 22 | STAT3/MAPK3/MAPK1/TP53/CTNNB1/HSP90AA1/EGFR/MAPK14/MAPK8/SMAD3/PTK2/FOXO1/PPARA/SMAD2/MET/PTPN1/BCL2/SOD1/MAPT/MAP3K5/CDH2/LGALS3 |
| MF | GO:0019838 | growth factor binding | 2.32E-16 | 19 | EGFR/ITGB3/KDR/THBS1/IL2RB/COL1A2/ENG/TGFB3/TGFBR2/FGFR3/IL6ST/TGFBR1/IL6R/COL1A1/FGFR1/ITGA6/COL3A1/IGFBP3/FLT1 |
| MF | GO:0008083 | growth factor activity | 7.88E-16 | 20 | IL6/VEGFA/IL10/TGFB1/FGF2/LIF/CXCL12/HGF/IL4/CSF2/CXCL1/IGF1/OSM/TIMP1/CSF3/NGF/BDNF/TGFB3/TGFB2/FGF23 |
| MF | GO:0140297 | DNA-binding transcription factor binding | 3.58E-14 | 29 | STAT3/TP53/CTNNB1/ESR1/FOS/MAPK14/SMAD3/HIF1A/SMAD4/HDAC1/SP1/AR/CREB1/PPARA/PPARGC1A/SIRT1/PPARG/SMAD2/NFKBIA/HMGB1/NCOR2/E2F1/BCL2/YAP1/CDKN2A/CALR/NLRP3/PARP1/PCNA |

TABLE S5 KEGG pathways analysis of the core genes in PPI (dual-positive).

| **ID** | **Description** | **pvalue** | **p.adjust** | **qvalue** | **geneID** |
| --- | --- | --- | --- | --- | --- |
| hsa04933 | AGE-RAGE signaling pathway in diabetic complications | 2.67E-43 | 3.11E-41 | 8.42E-42 | STAT3/MAPK3/MAPK1/PIK3CA/AKT1/IL6/VEGFA/TNF/NFKB1/FN1/MAPK14/JAK2/IL1B/RAC1/MMP2/MAPK8/SMAD3/TGFB1/SMAD4/FOXO1/CCL2/CXCL8/EDN1/CASP3/IL1A/SMAD2/CCND1/PIK3CB/COL1A2/VCAM1/TGFB3/BCL2/MAPK10/TGFBR2/TGFBR1/SERPINE1/TGFB2/COL1A1/COL3A1/AGER/ICAM1/EGR1 |
| hsa04668 | TNF signaling pathway | 2.36E-36 | 1.37E-34 | 3.73E-35 | MAPK3/MAPK1/PIK3CA/AKT1/IL6/TNF/NFKB1/FOS/MAPK14/IL1B/MAPK8/LIF/MMP9/CCL5/CASP8/CCL2/CREB1/EDN1/CASP3/CXCL10/CSF2/MMP3/MAP2K1/CXCL1/CHUK/NFKBIA/CXCL2/CCL20/SOCS3/PIK3CB/TNFRSF1A/VCAM1/MAPK10/MAP3K5/TNFRSF1B/PTGS2/FAS/CASP7/ICAM1 |
| hsa04657 | IL-17 signaling pathway | 6.58E-31 | 2.19E-29 | 5.93E-30 | MAPK3/MAPK1/IL6/HSP90AA1/TNF/NFKB1/FOS/MAPK14/IL1B/MAPK8/MMP9/CASP8/CCL2/IL4/MMP1/CXCL8/CASP3/CXCL10/CSF2/MMP3/IL17A/CXCL1/CHUK/NFKBIA/CSF3/CXCL2/IL17F/CCL20/LCN2/CCL11/IFNG/MAPK10/PTGS2 |
| hsa04068 | FoxO signaling pathway | 7.82E-27 | 1.30E-25 | 3.53E-26 | STAT3/MAPK3/MAPK1/PIK3CA/AKT1/IL6/EGFR/IL10/MAPK14/MAPK8/SMAD3/TGFB1/SMAD4/FOXO3/FOXO1/CDKN1A/SIRT1/MAP2K1/CHUK/IGF1/CCND1/MDM2/PIK3CB/PTEN/TGFB3/MAPK10/TGFBR2/TGFBR1/TGFB2/CAT/SOD2/PRKAB1/PRKAA1/TNFSF10 |
| hsa04010 | MAPK signaling pathway | 5.01E-24 | 6.48E-23 | 1.76E-23 | MAPK3/MAPK1/TP53/AKT1/EGFR/VEGFA/TNF/NFKB1/FOS/MAPK14/IL1B/RAC1/MAPK8/TGFB1/FGF2/HGF/CASP3/IL1A/KDR/MYD88/MAP2K1/CHUK/IGF1/MET/NGF/BDNF/TNFRSF1A/TGFB3/MAPK10/TGFBR2/FGFR3/MAPT/HSPA1A/TGFBR1/MAP3K5/ANGPT1/TEK/TGFB2/FGFR1/FAS/DUSP1/FLT1/FGF23/CD14 |
| hsa04620 | Toll-like receptor signaling pathway | 1.99E-21 | 1.72E-20 | 4.66E-21 | MAPK3/MAPK1/PIK3CA/AKT1/IL6/TNF/NFKB1/FOS/MAPK14/IL1B/RAC1/MAPK8/CCL5/TLR4/CASP8/CXCL8/CXCL10/MYD88/MAP2K1/CHUK/NFKBIA/TLR2/PIK3CB/CD40/MAPK10/SPP1/CD14 |
| hsa04926 | Relaxin signaling pathway | 8.86E-19 | 5.16E-18 | 1.40E-18 | MAPK3/MAPK1/PIK3CA/AKT1/EGFR/VEGFA/NFKB1/FOS/MAPK14/MMP2/MAPK8/TGFB1/MMP9/CREB1/MMP1/EDN1/MAP2K1/SMAD2/NFKBIA/PIK3CB/COL1A2/NOS2/MAPK10/TGFBR2/TGFBR1/COL1A1/COL3A1 |
| hsa04625 | C-type lectin receptor signaling pathway | 1.42E-15 | 5.61E-15 | 1.52E-15 | MAPK3/MAPK1/PIK3CA/AKT1/IL6/PTPN11/IL10/TNF/NFKB1/MAPK14/IL1B/MAPK8/CASP8/CHUK/CCL22/NFKBIA/MDM2/PIK3CB/MAPK10/PTGS2/CASP1/NLRP3 |
| hsa04064 | NF-kappa B signaling pathway | 1.76E-14 | 6.30E-14 | 1.71E-14 | TNF/NFKB1/IL1B/CXCL12/TLR4/CXCL8/MYD88/CXCL1/CHUK/NFKBIA/CXCL2/BCL2L1/TNFRSF1A/VCAM1/CD40/BCL2/PLAU/PTGS2/ICAM1/CD14/PARP1 |
| hsa04115 | p53 signaling pathway | 7.00E-12 | 2.12E-11 | 5.75E-12 | TP53/CASP8/CASP3/CDKN1A/IGF1/CCND1/MDM2/THBS1/BCL2L1/PTEN/BCL2/CDKN2A/CDK1/SERPINE1/FAS/IGFBP3 |
| hsa04660 | T cell receptor signaling pathway | 2.25E-11 | 6.39E-11 | 1.73E-11 | MAPK3/MAPK1/PIK3CA/AKT1/IL10/TNF/NFKB1/FOS/MAPK14/MAPK8/IL4/CSF2/MAP2K1/CHUK/NFKBIA/PIK3CB/IFNG/MAPK10 |
| hsa04915 | Estrogen signaling pathway | 1.17E-07 | 2.42E-07 | 6.56E-08 | MAPK3/MAPK1/PIK3CA/AKT1/HSP90AA1/EGFR/ESR1/FOS/MMP2/MMP9/SP1/CREB1/MAP2K1/PIK3CB/BCL2/HSPA1A |
| hsa04012 | ErbB signaling pathway | 5.60E-07 | 1.11E-06 | 3.00E-07 | MAPK3/MAPK1/PIK3CA/AKT1/EGFR/MAPK8/PTK2/CDKN1A/MAP2K1/MTOR/PIK3CB/MAPK10 |
| hsa04662 | B cell receptor signaling pathway | 2.82E-06 | 5.43E-06 | 1.47E-06 | MAPK3/MAPK1/PIK3CA/AKT1/NFKB1/FOS/RAC1/MAP2K1/CHUK/NFKBIA/PIK3CB |
| hsa04912 | GnRH signaling pathway | 5.79E-05 | 0.000102947 | 2.79E-05 | MAPK3/MAPK1/EGFR/MAPK14/MMP2/MAPK8/MAP2K1/PTK2B/MAPK10/EGR1 |

TABLE S6 Differential expressed genes in GSE66360.

| **Gene ID** | | **logFC** | **AveExpr** | **t** | **P.Value** | **adj.P.Val** |
| --- | --- | --- | --- | --- | --- | --- |
| NR4A2 | 2.954843192 | | 7.935629559 | 12.23633839 | 1.06E-21 | 2.48E-17 |
| GABARAPL1 | 1.561858031 | | 8.445247303 | 11.13125412 | 2.72E-19 | 3.18E-15 |
| NFKBIZ | 1.38782861 | | 10.62270325 | 10.24895852 | 2.39E-17 | 1.86E-13 |
| PDE4B | 1.312278088 | | 8.513346103 | 9.883722682 | 1.53E-16 | 8.11E-13 |
| THBD | 1.947928456 | | 5.594770495 | 9.858980822 | 1.74E-16 | 8.11E-13 |
| IRAK3 | 2.37504932 | | 6.532625542 | 9.794187398 | 2.42E-16 | 9.40E-13 |
| IL1R2 | 2.544134565 | | 7.502802219 | 9.704280766 | 3.82E-16 | 1.24E-12 |
| ACSL1 | 2.206741874 | | 8.189232828 | 9.683471099 | 4.24E-16 | 1.24E-12 |
| NFIL3 | 2.577387599 | | 8.537520145 | 9.53070518 | 9.23E-16 | 2.39E-12 |
| MAP3K8 | 1.636104505 | | 7.667520027 | 9.488799369 | 1.14E-15 | 2.67E-12 |
| ZFP36 | 1.961919852 | | 10.14264381 | 9.409949306 | 1.71E-15 | 3.53E-12 |
| PELI1 | 1.929588175 | | 8.919187071 | 9.397471469 | 1.82E-15 | 3.53E-12 |
| CLEC4E | 1.808458942 | | 7.2875288 | 9.308645182 | 2.85E-15 | 5.12E-12 |
| BCL6 | 1.759041347 | | 7.465515502 | 9.275062539 | 3.38E-15 | 5.64E-12 |
| S100A12 | 2.931322541 | | 7.770745765 | 9.244388483 | 3.95E-15 | 6.15E-12 |
| NAMPT | 2.028228706 | | 9.636309965 | 9.116765893 | 7.56E-15 | 1.10E-11 |
| SLC11A1 | 1.288396566 | | 6.659260862 | 9.095156562 | 8.43E-15 | 1.16E-11 |
| IL1B | 2.73288776 | | 9.225926767 | 8.995140033 | 1.40E-14 | 1.81E-11 |
| PPP1R15A | 2.035696999 | | 8.187084149 | 8.600831072 | 1.03E-13 | 1.26E-10 |
| CCL20 | 2.759120086 | | 8.450554588 | 8.574855068 | 1.17E-13 | 1.37E-10 |
| BCL3 | 1.166362641 | | 6.758085864 | 8.475438127 | 1.93E-13 | 2.15E-10 |
| S100P | 2.345724788 | | 8.36782202 | 8.463904745 | 2.05E-13 | 2.17E-10 |
| LOC100129518 | 1.81337901 | | 9.654950061 | 8.381361329 | 3.10E-13 | 3.05E-10 |
| ITPRIP | 1.207673504 | | 6.922634594 | 8.379168953 | 3.13E-13 | 3.05E-10 |
| HBEGF | 1.095234167 | | 5.737830569 | 8.354430576 | 3.55E-13 | 3.31E-10 |
| JUN | 1.712802601 | | 9.518514964 | 8.328104771 | 4.05E-13 | 3.36E-10 |
| FCN1 | 2.187271598 | | 7.102034803 | 8.325249264 | 4.11E-13 | 3.36E-10 |
| GADD45A | 1.45922695 | | 9.193344376 | 8.322215371 | 4.17E-13 | 3.36E-10 |
| NLRP3 | 2.153937211 | | 6.263294903 | 8.307384147 | 4.49E-13 | 3.46E-10 |
| VCAN | 2.478226355 | | 7.085447654 | 8.302977755 | 4.59E-13 | 3.46E-10 |
| CLEC4D | 2.417168837 | | 6.095454891 | 8.290390721 | 4.89E-13 | 3.57E-10 |
| FOSL2 | 1.024302295 | | 7.122335852 | 8.257117139 | 5.78E-13 | 3.97E-10 |
| FCER1G | 2.73342441 | | 8.491942253 | 8.257113059 | 5.78E-13 | 3.97E-10 |
| MAFB | 2.023694397 | | 7.536192995 | 8.19242239 | 7.99E-13 | 5.31E-10 |
| ADIPOR1 | 1.084485846 | | 9.196528555 | 8.184649906 | 8.31E-13 | 5.31E-10 |
| NR4A3 | 1.652690622 | | 5.646902316 | 8.182073051 | 8.42E-13 | 5.31E-10 |
| LILRB2 | 2.107115876 | | 7.428944052 | 8.159724132 | 9.41E-13 | 5.78E-10 |
| GLUL | 1.250911576 | | 7.7949019 | 8.153801307 | 9.69E-13 | 5.80E-10 |
| ITLN1 | 2.815885654 | | 6.765521987 | 8.137769891 | 1.05E-12 | 6.13E-10 |
| JDP2 | 1.237355639 | | 6.000745627 | 8.074550427 | 1.44E-12 | 8.20E-10 |
| PLAUR | 1.848464173 | | 7.269474198 | 7.993085868 | 2.16E-12 | 1.19E-09 |
| PMAIP1 | 1.627055089 | | 9.149118406 | 7.986691218 | 2.23E-12 | 1.19E-09 |
| NFKBIA | 1.787306639 | | 9.451766858 | 7.985778729 | 2.24E-12 | 1.19E-09 |
| IL1RN | 1.381513463 | | 5.927714057 | 7.903496307 | 3.38E-12 | 1.75E-09 |
| TLR2 | 2.10530236 | | 7.152808207 | 7.899448775 | 3.44E-12 | 1.75E-09 |
| SERPINA1 | 1.544308096 | | 6.371337602 | 7.86406698 | 4.11E-12 | 2.04E-09 |
| TREM1 | 2.608776517 | | 7.408581792 | 7.855722813 | 4.28E-12 | 2.08E-09 |
| TP53INP2 | 2.242896115 | | 7.877895928 | 7.851826961 | 4.36E-12 | 2.08E-09 |
| LYZ | 2.272492438 | | 9.110349416 | 7.847582739 | 4.46E-12 | 2.08E-09 |
| CDA | 1.686422368 | | 6.423406268 | 7.832602444 | 4.80E-12 | 2.20E-09 |
| QPCT | 2.149533287 | | 6.899552321 | 7.811194885 | 5.34E-12 | 2.39E-09 |
| SLC7A7 | 2.047098914 | | 7.557811925 | 7.78848025 | 5.97E-12 | 2.63E-09 |
| HAL | 1.226606778 | | 5.561464373 | 7.752545596 | 7.13E-12 | 3.08E-09 |
| IER3 | 2.065399112 | | 10.92512027 | 7.718723446 | 8.43E-12 | 3.55E-09 |
| S100A9 | 2.103261101 | | 9.289691588 | 7.716580626 | 8.52E-12 | 3.55E-09 |
| CCL4 | 2.124382833 | | 9.066221067 | 7.632160645 | 1.29E-11 | 5.29E-09 |
| CXCL2 | 1.97416066 | | 6.190732771 | 7.620727143 | 1.37E-11 | 5.50E-09 |
| CD83 | 2.256019153 | | 9.214798278 | 7.605467847 | 1.47E-11 | 5.78E-09 |
| C5AR1 | 2.346791325 | | 9.111418562 | 7.603851333 | 1.49E-11 | 5.78E-09 |
| CSTA | 2.781419879 | | 8.381503484 | 7.587486413 | 1.61E-11 | 6.16E-09 |
| CLEC7A | 1.539437838 | | 7.18283445 | 7.584127876 | 1.64E-11 | 6.17E-09 |
| GAB2 | 1.420977764 | | 7.618930525 | 7.568829615 | 1.77E-11 | 6.54E-09 |
| CD55 | 1.051700886 | | 10.08433256 | 7.557216255 | 1.87E-11 | 6.82E-09 |
| SNORD89 | 1.613559086 | | 6.427785647 | 7.548987095 | 1.95E-11 | 6.99E-09 |
| APOBEC3A | 2.230155138 | | 7.776318375 | 7.518144692 | 2.27E-11 | 7.89E-09 |
| THBS1 | 1.555819635 | | 5.812787486 | 7.494648033 | 2.54E-11 | 8.73E-09 |
| MIR6883 | 1.409354543 | | 8.645407457 | 7.488718165 | 2.62E-11 | 8.85E-09 |
| CCL3 | 2.662837443 | | 9.139541769 | 7.475550093 | 2.79E-11 | 9.31E-09 |
| PHACTR1 | 1.888753114 | | 7.521714442 | 7.439821749 | 3.33E-11 | 1.09E-08 |
| SULF2 | 1.567668245 | | 5.70392826 | 7.438160732 | 3.35E-11 | 1.09E-08 |
| BST1 | 2.070984308 | | 6.594781462 | 7.415684937 | 3.74E-11 | 1.20E-08 |
| TRIB1 | 1.039076448 | | 6.629991242 | 7.398124279 | 4.08E-11 | 1.29E-08 |
| ETS2 | 1.444975367 | | 7.772027685 | 7.378376455 | 4.49E-11 | 1.40E-08 |
| AIF1 | 1.597948031 | | 7.560324634 | 7.208267271 | 1.03E-10 | 3.08E-08 |
| GADD45B | 1.368904775 | | 8.412424593 | 7.201820113 | 1.06E-10 | 3.14E-08 |
| ALDH2 | 2.090453551 | | 6.848563754 | 7.194549053 | 1.10E-10 | 3.21E-08 |
| ICAM1 | 1.388183795 | | 6.645202395 | 7.183093486 | 1.16E-10 | 3.35E-08 |
| CREB5 | 1.371611742 | | 5.846586452 | 7.150339583 | 1.36E-10 | 3.88E-08 |
| TULP2 | 1.773768331 | | 6.002300225 | 7.143575048 | 1.41E-10 | 3.96E-08 |
| AQP9 | 2.37796374 | | 7.560749464 | 7.131614652 | 1.49E-10 | 4.15E-08 |
| FCGR2A | 1.542627695 | | 7.69197632 | 7.101192091 | 1.73E-10 | 4.75E-08 |
| FOS | 2.077979356 | | 8.66445713 | 7.089183895 | 1.83E-10 | 4.97E-08 |
| MAFF | 1.556893804 | | 8.165850628 | 7.082382914 | 1.89E-10 | 5.02E-08 |
| CSF3R | 2.165835192 | | 7.254591009 | 7.074191064 | 1.97E-10 | 5.17E-08 |
| CMTM2 | 1.973429886 | | 7.898179764 | 7.064593263 | 2.06E-10 | 5.35E-08 |
| CPD | 1.108563614 | | 8.692845935 | 7.050323851 | 2.21E-10 | 5.67E-08 |
| DYSF | 1.859952684 | | 7.087212041 | 7.044846867 | 2.27E-10 | 5.76E-08 |
| FOSB | 1.712890777 | | 7.904915471 | 7.014836862 | 2.62E-10 | 6.58E-08 |
| IL13RA1 | 1.482073425 | | 5.589201482 | 6.906861397 | 4.41E-10 | 1.08E-07 |
| VNN1 | 1.858837059 | | 5.478737863 | 6.902510025 | 4.50E-10 | 1.09E-07 |
| SKIL | 1.406069091 | | 6.904053255 | 6.896438735 | 4.63E-10 | 1.10E-07 |
| PYGL | 1.896195546 | | 7.212241832 | 6.895216691 | 4.66E-10 | 1.10E-07 |
| CXCL16 | 1.881414262 | | 8.636829724 | 6.8951436 | 4.66E-10 | 1.10E-07 |
| TP53BP2 | 1.137534394 | | 8.508277877 | 6.812300638 | 6.93E-10 | 1.62E-07 |
| EFEMP1 | 1.482115421 | | 4.11882004 | 6.809645037 | 7.02E-10 | 1.62E-07 |
| PDZD8 | 1.073844629 | | 8.097695522 | 6.801306275 | 7.30E-10 | 1.67E-07 |
| DDIT3 | 1.755470269 | | 7.95954969 | 6.797623877 | 7.43E-10 | 1.67E-07 |
| CCRL2 | 1.741443847 | | 6.500953174 | 6.793956343 | 7.56E-10 | 1.68E-07 |
| GIMAP7 | -1.587547241 | | 10.22001089 | -6.772964699 | 8.36E-10 | 1.83E-07 |
| BCL2A1 | 1.852058825 | | 10.39357233 | 6.772184436 | 8.39E-10 | 1.83E-07 |
| CH25H | 1.432014561 | | 4.761767088 | 6.767467297 | 8.58E-10 | 1.85E-07 |
| MGP | 1.517511178 | | 5.949958706 | 6.751601726 | 9.25E-10 | 1.98E-07 |
| LRG1 | 1.442596843 | | 6.016469853 | 6.746534698 | 9.48E-10 | 2.01E-07 |
| MMP9 | 1.465394957 | | 6.687222359 | 6.730602298 | 1.02E-09 | 2.15E-07 |
| SIRPA | 1.078997498 | | 6.58883194 | 6.674598804 | 1.33E-09 | 2.72E-07 |
| GIMAP6 | -1.494459408 | | 9.336669188 | -6.674445717 | 1.33E-09 | 2.72E-07 |
| CDKN1A | 1.630957096 | | 7.493401791 | 6.648023808 | 1.51E-09 | 3.04E-07 |
| LILRA5 | 1.118834401 | | 5.289563664 | 6.63104197 | 1.64E-09 | 3.19E-07 |
| ZNF137P | -1.423771375 | | 7.080755232 | -6.630729213 | 1.64E-09 | 3.19E-07 |
| RGS1 | 1.268569432 | | 6.805903693 | 6.626155495 | 1.68E-09 | 3.23E-07 |
| DUSP1 | 1.0161507 | | 8.098711265 | 6.610414034 | 1.81E-09 | 3.45E-07 |
| PPIF | 1.233200599 | | 7.614194361 | 6.588690816 | 2.00E-09 | 3.78E-07 |
| MIR22 | 1.583295042 | | 7.942599141 | 6.588106322 | 2.01E-09 | 3.78E-07 |
| KLF4 | 1.372592929 | | 6.53474893 | 6.56949348 | 2.19E-09 | 4.09E-07 |
| FPR1 | 1.61165281 | | 6.647722906 | 6.559667856 | 2.29E-09 | 4.25E-07 |
| RBP7 | 1.730375458 | | 7.855478421 | 6.527265651 | 2.67E-09 | 4.87E-07 |
| PILRA | 1.577478675 | | 7.62090079 | 6.514025163 | 2.84E-09 | 5.14E-07 |
| MME | 1.501920685 | | 6.499575337 | 6.48576095 | 3.25E-09 | 5.83E-07 |
| PAQR8 | -1.132569589 | | 7.218807884 | -6.480691844 | 3.33E-09 | 5.92E-07 |
| ZEB2 | 1.195261586 | | 6.400704061 | 6.47647894 | 3.39E-09 | 6.00E-07 |
| DOCK4 | 1.117401314 | | 5.603342441 | 6.464757845 | 3.58E-09 | 6.29E-07 |
| CCR2 | -1.534842987 | | 7.406303937 | -6.431967742 | 4.18E-09 | 7.27E-07 |
| FAM198B | 1.407773184 | | 5.223990122 | 6.414204982 | 4.54E-09 | 7.84E-07 |
| WDFY3 | 1.232826992 | | 5.633529152 | 6.392284837 | 5.03E-09 | 8.44E-07 |
| DOCK5 | 1.045229609 | | 6.16009564 | 6.389183756 | 5.10E-09 | 8.50E-07 |
| CD33 | 1.534760175 | | 5.578679959 | 6.387467828 | 5.14E-09 | 8.51E-07 |
| PTX3 | 2.610458126 | | 6.8991597 | 6.373059515 | 5.50E-09 | 9.04E-07 |
| MGAM | 1.946870914 | | 7.634927774 | 6.355576037 | 5.97E-09 | 9.73E-07 |
| TNFAIP2 | 1.225714231 | | 6.577582355 | 6.328029604 | 6.78E-09 | 1.10E-06 |
| SLC2A14 | 1.307635429 | | 9.798611196 | 6.292915375 | 7.98E-09 | 1.28E-06 |
| CTD-2541M15.1 | -1.373411276 | | 8.450409075 | -6.278250926 | 8.54E-09 | 1.36E-06 |
| AC079305.10 | 1.948638114 | | 5.651369651 | 6.268045277 | 8.95E-09 | 1.41E-06 |
| SLC2A3 | 1.063201533 | | 10.06352296 | 6.259544406 | 9.31E-09 | 1.45E-06 |
| METRNL | 1.130878615 | | 6.520727377 | 6.243061645 | 1.01E-08 | 1.54E-06 |
| SYTL3 | 1.049998532 | | 7.581693862 | 6.230547592 | 1.06E-08 | 1.60E-06 |
| CSRNP1 | 1.155230531 | | 8.191423112 | 6.22386213 | 1.10E-08 | 1.64E-06 |
| LILRA2 | 1.294019446 | | 6.050259863 | 6.214592628 | 1.15E-08 | 1.70E-06 |
| GLT1D1 | 1.634326342 | | 6.205718744 | 6.208823665 | 1.18E-08 | 1.74E-06 |
| RLF | 1.107554858 | | 9.407284895 | 6.168337786 | 1.42E-08 | 2.04E-06 |
| MS4A6A | 1.463420883 | | 6.635268981 | 6.158201675 | 1.49E-08 | 2.13E-06 |
| LINC00528 | 1.41280089 | | 7.277668572 | 6.149335695 | 1.55E-08 | 2.20E-06 |
| RAB32 | 1.355962307 | | 5.812230093 | 6.137584888 | 1.63E-08 | 2.31E-06 |
| FPR2 | 1.602456861 | | 5.977829631 | 6.109536757 | 1.86E-08 | 2.60E-06 |
| SLC7A5 | 1.276659326 | | 9.113623005 | 6.106233495 | 1.89E-08 | 2.62E-06 |
| CCR5 | -1.489876733 | | 8.375681765 | -6.099902537 | 1.94E-08 | 2.68E-06 |
| TLR4 | 1.166475633 | | 5.907241865 | 6.098741793 | 1.95E-08 | 2.68E-06 |
| CTD-2528L19.6 | -1.730609397 | | 5.64022877 | -6.097220199 | 1.97E-08 | 2.68E-06 |
| SAMSN1 | 1.482038933 | | 7.684259947 | 6.090288883 | 2.03E-08 | 2.75E-06 |
| CD36 | 1.284180997 | | 6.01085995 | 6.061893795 | 2.31E-08 | 3.10E-06 |
| PLBD1 | 2.012991213 | | 8.308072141 | 6.061737027 | 2.31E-08 | 3.10E-06 |
| VNN3 | 1.112105894 | | 5.36381755 | 6.059302286 | 2.34E-08 | 3.12E-06 |
| LOC100631377 | -1.187883405 | | 8.073816776 | -6.041225466 | 2.54E-08 | 3.37E-06 |
| CD93 | 1.425556211 | | 6.831726226 | 6.008417373 | 2.95E-08 | 3.85E-06 |
| SECTM1 | 1.268445319 | | 6.214176059 | 6.007923695 | 2.95E-08 | 3.85E-06 |
| P2RY13 | 1.924815051 | | 5.165477605 | 6.000333726 | 3.06E-08 | 3.96E-06 |
| AK026714 | 1.283441565 | | 9.841740511 | 5.996132419 | 3.12E-08 | 4.02E-06 |
| MBOAT2 | 1.062836224 | | 6.501859341 | 5.947752655 | 3.88E-08 | 4.95E-06 |
| GNA15 | 1.052686509 | | 6.343548799 | 5.936668673 | 4.08E-08 | 5.15E-06 |
| TSIX | -3.036471578 | | 6.855013325 | -5.929408981 | 4.22E-08 | 5.26E-06 |
| MXD1 | 1.098128579 | | 8.90357943 | 5.925246085 | 4.30E-08 | 5.32E-06 |
| CPVL | 1.853482744 | | 6.015874371 | 5.921622945 | 4.37E-08 | 5.36E-06 |
| PTAFR | 1.155839478 | | 6.111038046 | 5.91577429 | 4.48E-08 | 5.42E-06 |
| EREG | 1.706590663 | | 6.575163744 | 5.912019249 | 4.56E-08 | 5.48E-06 |
| HCAR3 | 2.035985969 | | 7.665445179 | 5.909576477 | 4.61E-08 | 5.48E-06 |
| LPCAT2 | 1.227463561 | | 5.275271248 | 5.908824791 | 4.63E-08 | 5.48E-06 |
| ANXA3 | 1.849506178 | | 5.503919113 | 5.893508103 | 4.96E-08 | 5.81E-06 |
| CISH | -1.334959002 | | 7.858759022 | -5.881886387 | 5.23E-08 | 6.10E-06 |
| C9orf72 | 1.122284753 | | 6.920515329 | 5.868293199 | 5.56E-08 | 6.45E-06 |
| RNASE2 | 2.031099711 | | 6.525799931 | 5.859967837 | 5.77E-08 | 6.66E-06 |
| ZNF420 | -1.151023803 | | 7.17873439 | -5.833874446 | 6.49E-08 | 7.38E-06 |
| GPR84 | 1.086497605 | | 4.277693154 | 5.832221673 | 6.53E-08 | 7.40E-06 |
| CXCL1 | 1.972766934 | | 7.862475446 | 5.804864265 | 7.39E-08 | 8.30E-06 |
| SAT1 | 1.146428762 | | 11.10202551 | 5.796559107 | 7.67E-08 | 8.56E-06 |
| TMCC3 | 1.185393083 | | 6.53163916 | 5.792162931 | 7.82E-08 | 8.69E-06 |
| CD163 | 1.578475694 | | 5.442385978 | 5.769125566 | 8.67E-08 | 9.58E-06 |
| CD14 | 1.668958981 | | 6.732785199 | 5.749582473 | 9.46E-08 | 1.04E-05 |
| XIST | -2.888947771 | | 5.626617402 | -5.731116018 | 1.03E-07 | 1.10E-05 |
| MCEMP1 | 1.25064028 | | 7.026647833 | 5.718925706 | 1.08E-07 | 1.16E-05 |
| RNF175 | 1.473508588 | | 7.983655226 | 5.678547079 | 1.30E-07 | 1.36E-05 |
| EPAS1 | 1.005586662 | | 5.682856938 | 5.675211917 | 1.32E-07 | 1.37E-05 |
| CXCL8 | 1.40755542 | | 10.91560384 | 5.667100621 | 1.36E-07 | 1.42E-05 |
| CD300LF | 1.727951648 | | 5.666093188 | 5.655666006 | 1.44E-07 | 1.48E-05 |
| EMR2 | 1.116101794 | | 5.398415477 | 5.646912771 | 1.49E-07 | 1.53E-05 |
| DMXL2 | 1.548585856 | | 7.242812254 | 5.636071658 | 1.57E-07 | 1.60E-05 |
| IFNGR1 | 1.026003009 | | 9.846652547 | 5.623617847 | 1.65E-07 | 1.66E-05 |
| GZMA | -1.053624769 | | 11.09367491 | -5.615533825 | 1.71E-07 | 1.71E-05 |
| LY96 | 1.059257368 | | 9.919795287 | 5.611048319 | 1.75E-07 | 1.74E-05 |
| RP11-214K3.19 | -1.113234353 | | 8.054136526 | -5.602270792 | 1.82E-07 | 1.80E-05 |
| FCGR3B | 1.665676568 | | 9.299119194 | 5.575745436 | 2.04E-07 | 1.99E-05 |
| BCL10 | 1.020641575 | | 6.994686939 | 5.559265072 | 2.20E-07 | 2.13E-05 |
| GPR97 | 1.178868799 | | 6.069961112 | 5.526650884 | 2.53E-07 | 2.42E-05 |
| CRTAM | -1.530626005 | | 6.621331314 | -5.525845213 | 2.54E-07 | 2.42E-05 |
| ARHGEF40 | 1.080132879 | | 7.332664827 | 5.52222712 | 2.58E-07 | 2.45E-05 |
| CHI3L1 | 1.264642699 | | 5.276852519 | 5.48681656 | 3.01E-07 | 2.83E-05 |
| GCSAM | -1.068670128 | | 6.12501784 | -5.468551332 | 3.26E-07 | 3.02E-05 |
| ADM | 1.803042995 | | 8.48926144 | 5.444994523 | 3.62E-07 | 3.31E-05 |
| ARL5B | 1.065778822 | | 7.38788111 | 5.43311352 | 3.81E-07 | 3.47E-05 |
| FOLR3 | 1.698075252 | | 6.128304203 | 5.416460052 | 4.09E-07 | 3.72E-05 |
| HAUS3 | 1.210145227 | | 8.78288112 | 5.411003264 | 4.19E-07 | 3.76E-05 |
| VNN2 | 1.333165729 | | 9.056128314 | 5.404511433 | 4.31E-07 | 3.84E-05 |
| RP11-96D1.11 | -1.056591755 | | 6.991290485 | -5.403667948 | 4.33E-07 | 3.84E-05 |
| KCTD12 | 1.562203011 | | 7.796036271 | 5.388652823 | 4.62E-07 | 4.00E-05 |
| DDX3Y | 1.490461678 | | 6.693593835 | 5.388374663 | 4.62E-07 | 4.00E-05 |
| LINC01000 | 1.054721666 | | 8.540879604 | 5.384933172 | 4.69E-07 | 4.03E-05 |
| LOC153682 | -1.373424596 | | 6.752798678 | -5.375069977 | 4.90E-07 | 4.19E-05 |
| TM6SF1 | 1.597669935 | | 5.792662665 | 5.357013997 | 5.29E-07 | 4.51E-05 |
| SCML1 | 1.145115517 | | 6.195254148 | 5.352979042 | 5.39E-07 | 4.57E-05 |
| CYP4F3 | 1.37640939 | | 6.041872241 | 5.341551414 | 5.66E-07 | 4.78E-05 |
| LOC284454 | 1.019973388 | | 8.565856297 | 5.340471504 | 5.68E-07 | 4.79E-05 |
| RP6-99M1.2 | 1.601128094 | | 7.237693188 | 5.33544967 | 5.81E-07 | 4.88E-05 |
| CEBPD | 1.197020634 | | 6.87090249 | 5.286339605 | 7.17E-07 | 5.91E-05 |
| RNF144B | 1.077090685 | | 6.696094718 | 5.280005538 | 7.37E-07 | 6.03E-05 |
| LYN | 1.204040089 | | 8.840301971 | 5.26694464 | 7.79E-07 | 6.31E-05 |
| EOMES | -1.342956487 | | 9.654421278 | -5.264432645 | 7.88E-07 | 6.36E-05 |
| SLC8A1-AS1 | 1.151832338 | | 5.528793391 | 5.255244874 | 8.19E-07 | 6.57E-05 |
| WDR86-AS1 | -1.08847962 | | 6.040688202 | -5.249023107 | 8.41E-07 | 6.70E-05 |
| MPP1 | 1.345882923 | | 7.206979681 | 5.245999648 | 8.52E-07 | 6.76E-05 |
| GIMAP4 | -1.578084483 | | 9.587149618 | -5.24119813 | 8.70E-07 | 6.88E-05 |
| TYROBP | 1.667831744 | | 9.434556421 | 5.238911018 | 8.78E-07 | 6.92E-05 |
| GIMAP8 | -1.131406162 | | 6.795023096 | -5.234996543 | 8.93E-07 | 7.02E-05 |
| RP11-443B7.1 | 1.029165463 | | 4.838098846 | 5.220612308 | 9.50E-07 | 7.39E-05 |
| RAB31 | 1.08902081 | | 8.34854462 | 5.220314717 | 9.51E-07 | 7.39E-05 |
| FURIN | 1.072151154 | | 9.004000842 | 5.19119514 | 1.08E-06 | 8.29E-05 |
| C15orf48 | 1.739045162 | | 7.588436068 | 5.187927709 | 1.09E-06 | 8.37E-05 |
| TIMP2 | 1.061843636 | | 8.422169504 | 5.184158383 | 1.11E-06 | 8.48E-05 |
| G0S2 | 1.278004802 | | 10.29945439 | 5.181215147 | 1.12E-06 | 8.56E-05 |
| ZFP3 | -1.243396132 | | 5.766420776 | -5.161132906 | 1.22E-06 | 9.26E-05 |
| S100A8 | 1.772162964 | | 10.81946283 | 5.159789084 | 1.23E-06 | 9.28E-05 |
| TNFAIP6 | 1.495789868 | | 6.460714466 | 5.140633316 | 1.33E-06 | 0.000100314 |
| SULF1 | 1.342049562 | | 5.253552524 | 5.125836335 | 1.42E-06 | 0.000105414 |
| B3GALT2 | -1.523079551 | | 6.097832102 | -5.124137386 | 1.43E-06 | 0.000105835 |
| MPEG1 | 1.844269136 | | 7.801803905 | 5.109775108 | 1.52E-06 | 0.000111724 |
| DUSP6 | 1.197513159 | | 7.891014402 | 5.085529889 | 1.68E-06 | 0.000121411 |
| KCNJ2 | 1.694627451 | | 8.237805589 | 5.075293238 | 1.75E-06 | 0.000125479 |
| EDN1 | 1.143337529 | | 6.886079456 | 5.069898046 | 1.79E-06 | 0.000127661 |
| DUSP4 | 1.11438183 | | 7.98180594 | 5.059885254 | 1.87E-06 | 0.000132443 |
| RAD54B | -1.158600071 | | 7.114507531 | -5.058776952 | 1.88E-06 | 0.000132536 |
| OGN | 1.180152801 | | 4.126170351 | 5.026647023 | 2.15E-06 | 0.000148003 |
| IDI2-AS1 | 1.01245884 | | 6.885226707 | 5.016725285 | 2.24E-06 | 0.000152901 |
| FCGR2A | 1.098557389 | | 5.303091267 | 5.000226548 | 2.40E-06 | 0.00016188 |
| PTGDR | -1.007678426 | | 7.005263487 | -4.985603239 | 2.55E-06 | 0.000169574 |
| RELT | 1.028583052 | | 6.648380426 | 4.97966765 | 2.61E-06 | 0.000172332 |
| FN1 | 1.018207575 | | 6.423728691 | 4.978442545 | 2.63E-06 | 0.000172722 |
| CLEC4A | 1.313358658 | | 7.047329675 | 4.974366871 | 2.67E-06 | 0.000175175 |
| FAM49A | 1.133424436 | | 6.362201842 | 4.955393944 | 2.89E-06 | 0.000185901 |
| ACKR3 | -1.126251173 | | 6.647524013 | -4.955326211 | 2.89E-06 | 0.000185901 |
| UTY | 1.335825645 | | 6.934244572 | 4.945885024 | 3.01E-06 | 0.000191199 |
| CTB-31O20.2 | 1.488112593 | | 7.718419616 | 4.942026039 | 3.06E-06 | 0.000193746 |
| CLEC12A | 1.109569273 | | 5.559184029 | 4.939976174 | 3.08E-06 | 0.000194865 |
| LILRB3 | 1.046699233 | | 7.96672315 | 4.932278396 | 3.18E-06 | 0.000198472 |
| RP11-747H7.3 | -1.038724132 | | 8.121384727 | -4.927060127 | 3.25E-06 | 0.000200781 |
| TNFAIP3 | 1.309889391 | | 10.82760611 | 4.917551879 | 3.38E-06 | 0.000207574 |
| LOC101928290 | 1.066675066 | | 5.995554205 | 4.910360885 | 3.48E-06 | 0.000213256 |
| CEBPB | 1.419535231 | | 9.304660517 | 4.888717611 | 3.81E-06 | 0.000230693 |
| IL23R | -1.101949375 | | 5.470317446 | -4.885454746 | 3.86E-06 | 0.000232598 |
| RASGEF1B | 1.098628831 | | 5.910835169 | 4.859684406 | 4.29E-06 | 0.000255885 |
| LINC00260 | -1.107978662 | | 6.561736062 | -4.856153464 | 4.35E-06 | 0.00025829 |
| HIST2H2BE | 1.23940761 | | 9.679960751 | 4.854590329 | 4.38E-06 | 0.000259289 |
| GIMAP1 | -1.041141423 | | 7.356069313 | -4.8513854 | 4.44E-06 | 0.000262047 |
| BRE-AS1 | 1.066029171 | | 5.098483869 | 4.843844457 | 4.57E-06 | 0.000267546 |
| ELOVL4 | -1.241015248 | | 7.774990105 | -4.840021001 | 4.65E-06 | 0.000269731 |
| TMEM176A | 1.187548399 | | 5.907265996 | 4.834283457 | 4.76E-06 | 0.000274761 |
| GIMAP1-GIMAP5 | -1.062160332 | | 9.461273505 | -4.821535347 | 5.01E-06 | 0.000284503 |
| ALDH1A1 | 1.427962267 | | 5.564954957 | 4.817990954 | 5.08E-06 | 0.000287919 |
| FGR | 1.825346216 | | 8.328008682 | 4.809311583 | 5.27E-06 | 0.000293353 |
| SIGLEC5 | 1.233658751 | | 6.047931832 | 4.80696632 | 5.32E-06 | 0.000294731 |
| NRGN | 1.287780064 | | 7.449774304 | 4.79948575 | 5.48E-06 | 0.000300981 |
| SLC15A3 | 1.112178576 | | 6.114610348 | 4.793172022 | 5.62E-06 | 0.000307231 |
| DENND2D | -1.097832189 | | 8.693045725 | -4.776878836 | 6.01E-06 | 0.000323554 |
| SLC31A2 | 1.179707808 | | 7.996316636 | 4.773163477 | 6.10E-06 | 0.000327237 |
| LOC731424 | 1.095196866 | | 5.925252589 | 4.747073658 | 6.78E-06 | 0.00035314 |
| LOC645984 | 1.353175531 | | 5.759737995 | 4.744221414 | 6.86E-06 | 0.000355646 |
| KIAA1598 | 1.119749281 | | 6.433919743 | 4.72596088 | 7.38E-06 | 0.000377813 |
| GCA | 1.357078726 | | 7.899418323 | 4.720253045 | 7.56E-06 | 0.00038576 |
| AMPD2 | 1.067764348 | | 9.221929867 | 4.716399547 | 7.67E-06 | 0.000390088 |
| RP11-373D23.2 | 1.282124071 | | 8.863701315 | 4.713352674 | 7.77E-06 | 0.000394045 |
| RP11-140I16.3 | -1.306924846 | | 5.940925715 | -4.70820244 | 7.93E-06 | 0.000399692 |
| CXCL3 | 1.364857727 | | 6.5565881 | 4.701798256 | 8.14E-06 | 0.000408357 |
| HMG20A | -1.064888218 | | 8.518051251 | -4.694856108 | 8.37E-06 | 0.000419008 |
| ZNF786 | -1.060282765 | | 7.043231249 | -4.690214565 | 8.53E-06 | 0.00042236 |
| FCGR1A | 1.057277696 | | 6.81059636 | 4.682759561 | 8.78E-06 | 0.000434265 |
| VSIG1 | -1.110580936 | | 6.292435369 | -4.619687557 | 1.13E-05 | 0.000543754 |
| TNF | 1.249835154 | | 7.961015982 | 4.613544115 | 1.16E-05 | 0.00055492 |
| FCGR1B | 1.251813154 | | 6.197216352 | 4.603407881 | 1.21E-05 | 0.00057186 |
| GVINP1 | -1.134183925 | | 8.317669866 | -4.591234031 | 1.27E-05 | 0.000594131 |
| LGALS2 | 1.118031072 | | 6.643387823 | 4.58832882 | 1.28E-05 | 0.000598606 |
| MARCKS | 1.03131874 | | 8.981034376 | 4.569649118 | 1.38E-05 | 0.000639634 |
| EPB41L3 | 1.000067352 | | 4.428926577 | 4.567481445 | 1.39E-05 | 0.000643661 |
| ZNF260 | -1.063904451 | | 8.514096284 | -4.558340102 | 1.44E-05 | 0.000660745 |
| FFAR2 | 1.122807666 | | 5.941487656 | 4.4999485 | 1.81E-05 | 0.000801526 |
| GABARAPL1 | 1.173065127 | | 10.76703917 | 4.490330927 | 1.88E-05 | 0.000823108 |
| ARRDC4 | 1.335279904 | | 5.847911733 | 4.488756112 | 1.89E-05 | 0.000826488 |
| CFP | 1.340849109 | | 6.687664065 | 4.482083682 | 1.95E-05 | 0.000844153 |
| TCEB3-AS1 | -1.048789721 | | 6.907953829 | -4.467804828 | 2.06E-05 | 0.000882175 |
| EIF1AY | 1.879079494 | | 6.350478151 | 4.462221429 | 2.10E-05 | 0.000899949 |
| AB488780 | -1.424143788 | | 8.186450939 | -4.457660956 | 2.14E-05 | 0.00091108 |
| TXLNGY | 1.848619648 | | 6.588344572 | 4.447050257 | 2.23E-05 | 0.000942643 |
| RPH3A | 1.02808159 | | 4.468010408 | 4.439741438 | 2.29E-05 | 0.000964589 |
| CSF1R | 1.082408663 | | 5.423608277 | 4.4158264 | 2.52E-05 | 0.001034212 |
| SGK1 | 1.144719302 | | 10.53826026 | 4.399518474 | 2.68E-05 | 0.001088208 |
| CTD-3025N20.3 | -1.100235317 | | 4.655711296 | -4.391391546 | 2.77E-05 | 0.001113113 |
| FTH1 | 1.09623375 | | 10.7770065 | 4.365412109 | 3.06E-05 | 0.001201565 |
| IRS2 | 1.104996334 | | 10.52105625 | 4.36136131 | 3.11E-05 | 0.00121431 |
| GIN1 | -1.165103106 | | 6.592535314 | -4.360435987 | 3.12E-05 | 0.001216603 |
| ZNF331 | 1.058179361 | | 8.891727116 | 4.351117105 | 3.23E-05 | 0.001250523 |
| LEO1 | -1.059851564 | | 7.294825991 | -4.349875031 | 3.25E-05 | 0.001254424 |
| PLEK | 1.007612213 | | 8.725815933 | 4.347718112 | 3.27E-05 | 0.001262769 |
| POP5 | -1.034808786 | | 9.199239341 | -4.343663661 | 3.33E-05 | 0.001278352 |
| HCK | 1.098624556 | | 6.963565375 | 4.342894117 | 3.34E-05 | 0.001278449 |
| CD302 | 1.17745968 | | 9.277205517 | 4.34218969 | 3.34E-05 | 0.001279281 |
| AMIGO2 | -1.252299142 | | 7.175167844 | -4.339727268 | 3.38E-05 | 0.00128931 |
| RP11-722E23.2 | -1.017066541 | | 6.374981707 | -4.331170798 | 3.49E-05 | 0.001327957 |
| PLA2G7 | 1.173052804 | | 4.285551694 | 4.312252595 | 3.75E-05 | 0.001412146 |
| KLF10 | 1.15544263 | | 8.973423795 | 4.307971163 | 3.81E-05 | 0.001430192 |
| GZMB | 1.743464559 | | 7.022409277 | 4.303900302 | 3.87E-05 | 0.001443312 |
| PRSS35 | -1.258799573 | | 5.374211135 | -4.302089862 | 3.90E-05 | 0.00144869 |
| TNFAIP8L2 | -1.064402958 | | 8.007780817 | -4.273578137 | 4.35E-05 | 0.001594526 |
| LINC00959 | -1.026421164 | | 6.745307952 | -4.263711529 | 4.51E-05 | 0.001645061 |
| EGR1 | 1.086024439 | | 9.776377736 | 4.234634353 | 5.04E-05 | 0.001792856 |
| EGR3 | 1.307661922 | | 7.812185087 | 4.225766015 | 5.21E-05 | 0.001836007 |
| RGS2 | 1.194258441 | | 10.4862004 | 4.209580945 | 5.54E-05 | 0.001919625 |
| ZNF185 | 1.003374965 | | 7.183526808 | 4.205028835 | 5.63E-05 | 0.001944136 |
| IFI30 | 1.079260393 | | 9.492525231 | 4.198765225 | 5.77E-05 | 0.001975867 |
| TTTY15 | 1.328848199 | | 5.821038705 | 4.195099225 | 5.85E-05 | 0.002000344 |
| LIF | 1.014341551 | | 5.574942933 | 4.165398467 | 6.54E-05 | 0.00218251 |
| SPARCL1 | 1.248307793 | | 5.584283102 | 4.163282083 | 6.59E-05 | 0.002195791 |
| IER5 | 1.136029744 | | 8.789210804 | 4.161984927 | 6.62E-05 | 0.002203321 |
| LOC101927069 | 1.265732017 | | 6.874759529 | 4.155157794 | 6.79E-05 | 0.002253831 |
| CSF2RB | 1.196861758 | | 8.973838306 | 4.144152547 | 7.07E-05 | 0.002331752 |
| USP9Y | 1.85305928 | | 6.134963801 | 4.138769638 | 7.22E-05 | 0.002370551 |
| CTC-510F12.4 | 1.11341313 | | 7.53253803 | 4.111520338 | 7.99E-05 | 0.002570985 |
| TLR8 | 1.153456558 | | 5.502301767 | 4.108224259 | 8.09E-05 | 0.002591893 |
| CTA-29F11.1 | 1.176995159 | | 7.886761845 | 4.103277986 | 8.24E-05 | 0.002629032 |
| DNMBP | 1.099644083 | | 7.949308855 | 4.086675033 | 8.76E-05 | 0.00275795 |
| DEFA1 | 1.676435827 | | 7.675429612 | 4.082498635 | 8.89E-05 | 0.002785575 |
| CD1D | 1.291759759 | | 6.187309549 | 4.05923701 | 9.69E-05 | 0.003003111 |
| NOG | -1.094804054 | | 7.129574618 | -4.057941462 | 9.74E-05 | 0.003006732 |
| FGL2 | 1.152758982 | | 7.850093392 | 4.021527858 | 0.000111299 | 0.003346029 |
| RTN1 | 1.032505306 | | 4.726102879 | 4.011314739 | 0.000115535 | 0.003423797 |
| A2M-AS1 | -1.167192638 | | 8.699903876 | -4.009905368 | 0.000116131 | 0.003434483 |
| mir-223 | 1.355851457 | | 6.897505669 | 3.983137071 | 0.000128035 | 0.003692928 |
| NCF2 | 1.183095417 | | 8.570887727 | 3.981836841 | 0.000128642 | 0.003705854 |
| CREB5 | 1.065533742 | | 6.835490971 | 3.963987432 | 0.000137258 | 0.003901078 |
| PTGS2 | 1.43730797 | | 7.74598774 | 3.961763025 | 0.00013837 | 0.003922302 |
| RPS4Y1 | 2.465372871 | | 9.727511451 | 3.961100694 | 0.000138703 | 0.003923012 |
| TMEM60 | -1.139536931 | | 7.720019465 | -3.956077229 | 0.00014125 | 0.0039758 |
| PFKFB3 | 1.13326756 | | 11.53339634 | 3.944227273 | 0.000147439 | 0.004100856 |
| OR52K3P | 1.105783218 | | 3.805568488 | 3.943349114 | 0.000147908 | 0.004105816 |
| GZMK | -1.115589168 | | 10.58076775 | -3.93497779 | 0.000152449 | 0.00419982 |
| KDM5D | 2.380027368 | | 8.098177044 | 3.908243766 | 0.00016786 | 0.004543895 |
| LOC283357 | -1.207075538 | | 8.114857149 | -3.876646997 | 0.000187992 | 0.004984783 |
| CCL14 | 1.043974608 | | 5.266139448 | 3.872296979 | 0.000190938 | 0.005036943 |
| GEMIN5 | -1.065566293 | | 6.246285667 | -3.866936218 | 0.000194629 | 0.005114266 |
| ZFY | 1.081355357 | | 4.278621609 | 3.858453603 | 0.000200608 | 0.00521602 |
| BTLA | -1.008701832 | | 7.900982483 | -3.847491522 | 0.000208595 | 0.005396194 |
| MEST | -1.038130693 | | 5.273870531 | -3.824359251 | 0.000226456 | 0.00571256 |
| ZNF792 | -1.192362932 | | 6.23942458 | -3.81890628 | 0.000230873 | 0.005805168 |
| GJA1 | 1.486985623 | | 7.709085616 | 3.802294098 | 0.000244842 | 0.006115709 |
| CLEC1A | 1.024667239 | | 3.384394954 | 3.797558626 | 0.000248969 | 0.00618685 |
| PKI55 | -1.006344953 | | 6.505907798 | -3.746965676 | 0.000297399 | 0.007117439 |
| SRGN | 1.017475901 | | 10.49746772 | 3.739589405 | 0.000305167 | 0.007266025 |
| LRRK2 | 1.357569344 | | 8.685902564 | 3.736694704 | 0.000308268 | 0.007287863 |
| MOP-1 | 1.041108721 | | 4.781273183 | 3.686788994 | 0.00036667 | 0.008330952 |
| AX746755 | -1.004148695 | | 7.462237112 | -3.685173373 | 0.000368726 | 0.008366833 |
| MNDA | 1.190819125 | | 8.112051981 | 3.66770434 | 0.000391665 | 0.008753936 |
| LOC100130357 | 1.109354541 | | 6.604609645 | 3.666589876 | 0.000393173 | 0.00877924 |
| MS4A7 | 1.035773913 | | 5.722937819 | 3.643055509 | 0.000426343 | 0.009306154 |
| TUBB2A | 1.33310856 | | 7.030482507 | 3.635052881 | 0.000438213 | 0.009441617 |
| ZNF189 | -1.069541879 | | 6.884704451 | -3.527598267 | 0.000631177 | 0.012481255 |
| LINC00094 | -1.023842041 | | 8.074358344 | -3.50535274 | 0.000680084 | 0.013235258 |
| SLC22A4 | 1.083908815 | | 5.335311657 | 3.423104278 | 0.00089373 | 0.016254321 |
| SERPINB2 | 1.16301064 | | 4.551323002 | 3.379487185 | 0.001031233 | 0.018005115 |
| ZNF204P | -1.006955459 | | 5.682932929 | -3.282829331 | 0.001409827 | 0.022485929 |
| PPBP | 1.105647536 | | 10.10067252 | 3.074938714 | 0.002704715 | 0.036354736 |
| ASH1L-AS1 | 1.029021595 | | 7.054039909 | 3.014318611 | 0.003252626 | 0.041519019 |

TABLE S7 Differential expressed genes in GSE75181.

| **Gene ID** | **logFC** | **AveExpr** | **t** | **P.Value** | **adj.P.Val** |
| --- | --- | --- | --- | --- | --- |
| SLC3A2 | -2.832111293 | 9.420892943 | -29.64652378 | 3.51E-20 | 1.10E-15 |
| SQLE | 1.459055585 | 8.972310244 | 20.00754604 | 2.65E-16 | 4.15E-12 |
| ATP6V0B | -1.5647114 | 9.825663762 | -18.94243477 | 8.96E-16 | 8.57E-12 |
| KLF2 | 1.14669118 | 10.08485212 | 18.77159587 | 1.10E-15 | 8.57E-12 |
| ZFAND2A | -2.901584174 | 10.56380008 | -16.55030902 | 1.75E-14 | 8.15E-11 |
| PDE7B | 1.406780194 | 7.996826168 | 16.51865934 | 1.82E-14 | 8.15E-11 |
| UGP2 | 1.231699071 | 9.337267926 | 16.28335615 | 2.49E-14 | 9.71E-11 |
| LOC731878 | -1.208412882 | 9.05324465 | -16.19726404 | 2.79E-14 | 9.71E-11 |
| TRIB3 | -2.895617639 | 9.530776567 | -15.91587501 | 4.09E-14 | 1.28E-10 |
| PPP1R15A | -3.042340059 | 11.25965502 | -15.83033162 | 4.59E-14 | 1.31E-10 |
| LOC646463 | -1.237727936 | 8.174340448 | -15.73803983 | 5.21E-14 | 1.36E-10 |
| C20orf111 | -2.402255898 | 10.09029393 | -15.65857482 | 5.81E-14 | 1.40E-10 |
| RPS7 | -2.498446082 | 9.516636525 | -15.44447652 | 7.82E-14 | 1.75E-10 |
| DDIT4 | -1.846576853 | 8.994408551 | -14.86844425 | 1.77E-13 | 3.46E-10 |
| PAQR4 | 1.066469518 | 8.067893737 | 14.79767485 | 1.96E-13 | 3.55E-10 |
| CCND2 | 1.141624848 | 9.724175392 | 14.76917035 | 2.04E-13 | 3.55E-10 |
| DEDD2 | -2.138542275 | 9.743709444 | -14.38412091 | 3.59E-13 | 5.72E-10 |
| ZHX3 | 1.057619616 | 7.873371653 | 14.13221503 | 5.22E-13 | 7.43E-10 |
| HSPA1B | -2.83515287 | 11.59719612 | -14.06857706 | 5.75E-13 | 7.82E-10 |
| KLHL21 | -1.406404633 | 8.057029725 | -13.54509574 | 1.28E-12 | 1.62E-09 |
| PARP1 | 1.219896064 | 8.970149894 | 13.37384812 | 1.67E-12 | 2.01E-09 |
| UPP1 | -1.829172402 | 8.914683259 | -13.32680212 | 1.80E-12 | 2.08E-09 |
| RGMA | 1.145811916 | 8.499918225 | 13.25972377 | 2.00E-12 | 2.23E-09 |
| LOC644132 | -1.695510522 | 8.41034766 | -13.0884604 | 2.62E-12 | 2.73E-09 |
| STIP1 | -1.415828622 | 9.349486294 | -13.04103099 | 2.83E-12 | 2.85E-09 |
| LOC641825 | -1.782093308 | 8.272968313 | -12.99792102 | 3.03E-12 | 2.96E-09 |
| TBC1D15 | -1.639126239 | 9.461823921 | -12.75066754 | 4.51E-12 | 4.28E-09 |
| GLA | -2.17015201 | 10.03036532 | -12.56234126 | 6.14E-12 | 5.34E-09 |
| UTP11L | -1.436492707 | 9.29549774 | -12.43463113 | 7.58E-12 | 6.14E-09 |
| SLC7A5 | -1.767535711 | 9.24015768 | -12.40979698 | 7.90E-12 | 6.18E-09 |
| FBXO32 | -1.64303883 | 8.744677466 | -12.31690112 | 9.22E-12 | 7.04E-09 |
| TMEM43 | 1.508647609 | 10.37144371 | 11.93756038 | 1.75E-11 | 1.19E-08 |
| GOLGA3 | 1.123601648 | 9.786561094 | 11.91143566 | 1.83E-11 | 1.19E-08 |
| BAG3 | -2.373549937 | 11.23035958 | -11.86372297 | 1.98E-11 | 1.19E-08 |
| BEX2 | -1.805261029 | 8.740080105 | -11.84932144 | 2.03E-11 | 1.19E-08 |
| MCM6 | 1.452481352 | 9.739589589 | 11.83496977 | 2.08E-11 | 1.19E-08 |
| DNMT1 | 1.04172716 | 10.06879251 | 11.81115543 | 2.17E-11 | 1.19E-08 |
| AKIRIN2 | -1.445736023 | 9.78567321 | -11.7202268 | 2.54E-11 | 1.34E-08 |
| DDIT3 | -3.029097916 | 9.461625866 | -11.59346218 | 3.16E-11 | 1.57E-08 |
| SMAD6 | 1.632421931 | 9.143122967 | 11.59320751 | 3.17E-11 | 1.57E-08 |
| HSPH1 | -2.46951172 | 11.18092029 | -11.54674875 | 3.43E-11 | 1.68E-08 |
| MKNK2 | -1.694613219 | 9.174450201 | -11.50479356 | 3.70E-11 | 1.75E-08 |
| C7orf40 | -1.460941998 | 8.904357863 | -11.47337066 | 3.90E-11 | 1.82E-08 |
| MOAP1 | -1.404304013 | 8.539736886 | -11.43752153 | 4.16E-11 | 1.88E-08 |
| LOC648390 | -1.322496184 | 11.98048219 | -11.43748227 | 4.16E-11 | 1.88E-08 |
| RNU6-1 | -2.657091651 | 11.24958607 | -11.42994139 | 4.21E-11 | 1.88E-08 |
| AXUD1 | -2.23257033 | 9.26234919 | -11.40184398 | 4.43E-11 | 1.95E-08 |
| C17orf91 | -1.887079103 | 10.10412552 | -11.30407455 | 5.26E-11 | 2.23E-08 |
| DUSP5 | -1.929224249 | 8.7128619 | -11.22413984 | 6.07E-11 | 2.50E-08 |
| FIBIN | 1.092094315 | 7.802435 | 11.17935009 | 6.57E-11 | 2.60E-08 |
| FKBP4 | -1.13730121 | 8.450317973 | -11.17283306 | 6.65E-11 | 2.60E-08 |
| PDXK | 1.227153304 | 8.807299505 | 11.16736554 | 6.72E-11 | 2.60E-08 |
| TNS3 | 2.188554751 | 10.51670687 | 11.14858573 | 6.95E-11 | 2.65E-08 |
| PHLDB1 | 1.394367036 | 9.022366734 | 11.05368589 | 8.24E-11 | 3.11E-08 |
| RNU6-15 | -2.591663358 | 11.31076321 | -11.03026593 | 8.59E-11 | 3.20E-08 |
| SSR1 | 1.200943534 | 9.696017124 | 10.95833378 | 9.79E-11 | 3.56E-08 |
| MIR1974 | -1.946363934 | 11.06390796 | -10.91051369 | 1.07E-10 | 3.75E-08 |
| ADAMTS5 | 1.99569053 | 8.312656548 | 10.88662311 | 1.12E-10 | 3.88E-08 |
| ISM1 | 1.766826099 | 9.938710948 | 10.86612097 | 1.16E-10 | 3.98E-08 |
| AHSA1 | -1.796191823 | 9.643094157 | -10.85881549 | 1.17E-10 | 3.99E-08 |
| HIST1H4H | -1.203360694 | 8.057588031 | -10.84833881 | 1.20E-10 | 4.02E-08 |
| RIOK3 | -1.410202957 | 9.725316387 | -10.7287488 | 1.49E-10 | 4.90E-08 |
| MXD4 | 1.300307934 | 9.796626846 | 10.72378738 | 1.50E-10 | 4.90E-08 |
| RNU1A3 | -3.551797809 | 9.843537139 | -10.70350612 | 1.56E-10 | 5.03E-08 |
| LPP | 1.310469951 | 9.939986985 | 10.68575173 | 1.61E-10 | 5.15E-08 |
| ZNHIT6 | 1.183178558 | 8.79832197 | 10.64328329 | 1.74E-10 | 5.51E-08 |
| FSCN1 | 1.087439274 | 12.22085076 | 10.61015058 | 1.85E-10 | 5.80E-08 |
| HBEGF | -1.99769639 | 8.354465507 | -10.59586959 | 1.90E-10 | 5.84E-08 |
| HMGCS1 | 1.651812328 | 9.642715248 | 10.58879806 | 1.93E-10 | 5.86E-08 |
| EEF2K | 1.014755531 | 8.318490683 | 10.51766886 | 2.20E-10 | 6.60E-08 |
| LOC399988 | -1.291019692 | 10.96408355 | -10.47268351 | 2.39E-10 | 6.84E-08 |
| SH3PXD2A | 1.12009124 | 8.61926194 | 10.46525769 | 2.43E-10 | 6.84E-08 |
| DIO2 | 1.701948552 | 8.608990127 | 10.44559483 | 2.52E-10 | 6.91E-08 |
| LOC387763 | -2.159272077 | 9.15273716 | -10.40607326 | 2.71E-10 | 7.25E-08 |
| LOC653171 | -1.014095993 | 8.641911622 | -10.39506505 | 2.77E-10 | 7.34E-08 |
| TRIB1 | -2.224749118 | 9.359771294 | -10.35863634 | 2.96E-10 | 7.67E-08 |
| ARIH1 | -1.415258869 | 8.431798156 | -10.33110429 | 3.12E-10 | 7.76E-08 |
| SLU7 | -1.76172243 | 8.96008543 | -10.33061821 | 3.13E-10 | 7.76E-08 |
| SLC40A1 | 2.33313044 | 9.024853348 | 10.29554127 | 3.34E-10 | 8.10E-08 |
| PEX11B | 1.0442241 | 8.498331898 | 10.28600562 | 3.40E-10 | 8.12E-08 |
| MAGED1 | 1.113587884 | 10.72736387 | 10.1889362 | 4.09E-10 | 9.55E-08 |
| TRAM2 | 1.907878986 | 9.476121826 | 10.15592071 | 4.35E-10 | 1.01E-07 |
| HIST2H2AC | -1.104952011 | 8.332588998 | -10.1439244 | 4.45E-10 | 1.03E-07 |
| GLS | 1.400524654 | 9.0759127 | 10.11850469 | 4.68E-10 | 1.06E-07 |
| MCL1 | -1.008578471 | 8.904726416 | -10.09623348 | 4.88E-10 | 1.08E-07 |
| VPS37B | -1.108743122 | 8.547186095 | -10.08047369 | 5.03E-10 | 1.10E-07 |
| DPYSL2 | 1.420350812 | 11.62378047 | 10.06057755 | 5.22E-10 | 1.13E-07 |
| LOC400013 | -1.541851438 | 8.993522204 | -10.01194371 | 5.74E-10 | 1.19E-07 |
| DNAJA1 | -1.483608921 | 12.05572444 | -10.00259238 | 5.84E-10 | 1.20E-07 |
| LOC730167 | -2.061932592 | 9.025194386 | -9.991668995 | 5.96E-10 | 1.22E-07 |
| ARHGAP21 | 1.156211788 | 10.47471315 | 9.96129943 | 6.32E-10 | 1.26E-07 |
| HIST2H2AA4 | -1.399461881 | 8.746020698 | -9.959644041 | 6.34E-10 | 1.26E-07 |
| CEBPG | -1.384209657 | 8.747226121 | -9.949855724 | 6.47E-10 | 1.27E-07 |
| TGFBR2 | 1.470491761 | 10.12680185 | 9.948594057 | 6.48E-10 | 1.27E-07 |
| ZNF622 | -1.450668001 | 9.724926068 | -9.94353399 | 6.54E-10 | 1.27E-07 |
| WDR68 | -1.062798659 | 8.41215499 | -9.921329119 | 6.83E-10 | 1.32E-07 |
| ANGPTL2 | 2.042436141 | 10.92451482 | 9.89297913 | 7.22E-10 | 1.38E-07 |
| GAS1 | 2.204327557 | 9.94634985 | 9.87607551 | 7.46E-10 | 1.41E-07 |
| AP1M1 | 1.150695291 | 9.177771756 | 9.869924582 | 7.55E-10 | 1.41E-07 |
| NR4A2 | -1.451905665 | 8.173183468 | -9.857646224 | 7.73E-10 | 1.44E-07 |
| GDF15 | -3.010322348 | 9.608414705 | -9.800208134 | 8.65E-10 | 1.56E-07 |
| RNU1-3 | -3.769095904 | 9.692504019 | -9.794262614 | 8.75E-10 | 1.57E-07 |
| RNU1-5 | -3.788278042 | 9.801739813 | -9.782001572 | 8.96E-10 | 1.60E-07 |
| LOC644877 | -1.679889318 | 9.109033038 | -9.75222126 | 9.50E-10 | 1.66E-07 |
| HIST2H2AA3 | -1.440084245 | 8.847364754 | -9.75031998 | 9.53E-10 | 1.66E-07 |
| ATF3 | -1.624759772 | 7.9500359 | -9.746555738 | 9.60E-10 | 1.66E-07 |
| GADD45G | -2.453143162 | 8.555236617 | -9.74438476 | 9.64E-10 | 1.66E-07 |
| DNAJB9 | -1.84483843 | 9.347062876 | -9.729816002 | 9.92E-10 | 1.69E-07 |
| LOC388796 | -1.517050944 | 8.286360413 | -9.681592154 | 1.09E-09 | 1.81E-07 |
| TOP2A | 1.607972919 | 8.532473154 | 9.648076146 | 1.16E-09 | 1.86E-07 |
| PSMA3 | -1.091220373 | 9.910588532 | -9.645729069 | 1.17E-09 | 1.86E-07 |
| PSMC4 | -1.032946094 | 8.823062858 | -9.642992736 | 1.18E-09 | 1.86E-07 |
| GEM | -2.053474693 | 8.924773099 | -9.641424845 | 1.18E-09 | 1.86E-07 |
| HSPA6 | -5.207997839 | 9.821477414 | -9.622802421 | 1.22E-09 | 1.92E-07 |
| TAC1 | -2.190202964 | 8.219266979 | -9.61883117 | 1.23E-09 | 1.92E-07 |
| LOC100132564 | -2.66325243 | 8.836768598 | -9.585474851 | 1.32E-09 | 1.99E-07 |
| RNU1G2 | -3.692813844 | 9.614892535 | -9.578930009 | 1.34E-09 | 2.01E-07 |
| SERPINE1 | -1.631199596 | 9.221986042 | -9.545162895 | 1.43E-09 | 2.11E-07 |
| NXF1 | -1.573196478 | 8.737876163 | -9.543445868 | 1.43E-09 | 2.11E-07 |
| LOC650803 | -1.10145623 | 8.13023652 | -9.491038083 | 1.59E-09 | 2.24E-07 |
| KIAA0907 | -1.626693463 | 9.395282185 | -9.490161236 | 1.59E-09 | 2.24E-07 |
| PIK3R2 | 1.074289261 | 9.615738468 | 9.47338808 | 1.65E-09 | 2.29E-07 |
| ULBP1 | -2.180545351 | 8.403522028 | -9.469095313 | 1.66E-09 | 2.30E-07 |
| RFTN2 | 1.013375527 | 8.74400596 | 9.404508723 | 1.89E-09 | 2.56E-07 |
| PXDN | 1.156116393 | 10.12693739 | 9.39253809 | 1.94E-09 | 2.61E-07 |
| RRP8 | -1.232809162 | 8.497997264 | -9.381856362 | 1.98E-09 | 2.66E-07 |
| GPX8 | 1.03249184 | 9.331731611 | 9.349125686 | 2.11E-09 | 2.81E-07 |
| H2AFY2 | 1.08190427 | 8.20674519 | 9.343547206 | 2.14E-09 | 2.81E-07 |
| SHRM | 1.160076341 | 8.000369515 | 9.310601808 | 2.28E-09 | 2.95E-07 |
| FAT1 | 1.005280919 | 8.992850677 | 9.307437214 | 2.30E-09 | 2.95E-07 |
| DPYSL3 | 1.10080662 | 10.08724819 | 9.287540132 | 2.39E-09 | 3.00E-07 |
| TEX10 | -1.442567147 | 8.396674925 | -9.279681784 | 2.43E-09 | 3.04E-07 |
| MYC | -1.711341404 | 8.825954078 | -9.260395784 | 2.52E-09 | 3.14E-07 |
| HSP90AB1 | -2.272618382 | 11.30440446 | -9.238426976 | 2.64E-09 | 3.25E-07 |
| FOS | -2.877414626 | 8.936686182 | -9.234254358 | 2.66E-09 | 3.27E-07 |
| RIT1 | -1.039814772 | 8.028192392 | -9.23112888 | 2.68E-09 | 3.27E-07 |
| BHLHB2 | -1.199815014 | 10.60516588 | -9.228602104 | 2.69E-09 | 3.27E-07 |
| CDK6 | 1.048533881 | 8.018046306 | 9.227692175 | 2.70E-09 | 3.27E-07 |
| CCDC59 | -2.004368544 | 10.29733961 | -9.217904647 | 2.75E-09 | 3.32E-07 |
| SELK | -1.093980848 | 8.937713353 | -9.20226281 | 2.84E-09 | 3.37E-07 |
| RNU1F1 | -3.136060133 | 9.228120983 | -9.190456522 | 2.91E-09 | 3.43E-07 |
| HSPA1A | -2.854947819 | 12.05809912 | -9.134232404 | 3.26E-09 | 3.72E-07 |
| GMDS | 1.376259167 | 11.09833 | 9.116177236 | 3.38E-09 | 3.82E-07 |
| HSPA7 | -5.001172798 | 9.762756883 | -9.079495903 | 3.64E-09 | 4.02E-07 |
| RGL1 | 1.539930535 | 9.034207151 | 9.05170211 | 3.86E-09 | 4.22E-07 |
| CTGF | 1.281775177 | 13.54508057 | 9.013650025 | 4.17E-09 | 4.50E-07 |
| CXXC5 | 1.216245477 | 10.22889497 | 9.005973348 | 4.24E-09 | 4.54E-07 |
| LOC646723 | -1.262326931 | 8.21326693 | -9.003548955 | 4.26E-09 | 4.55E-07 |
| GADD45A | -2.114145221 | 9.729750386 | -8.98942942 | 4.38E-09 | 4.65E-07 |
| RDBP | -1.10271668 | 8.522997466 | -8.956256795 | 4.69E-09 | 4.95E-07 |
| NCRNA00219 | -1.008535662 | 10.41756436 | -8.934925161 | 4.90E-09 | 5.10E-07 |
| H1F0 | 1.541032412 | 9.340439148 | 8.93234324 | 4.93E-09 | 5.11E-07 |
| BSDC1 | -1.042031 | 9.221864294 | -8.921093377 | 5.05E-09 | 5.18E-07 |
| NGDN | -1.05251056 | 8.612466613 | -8.874340355 | 5.56E-09 | 5.58E-07 |
| SLC16A3 | 1.194265841 | 9.973304145 | 8.873913252 | 5.56E-09 | 5.58E-07 |
| LOC729978 | -1.241180052 | 10.6177395 | -8.861527346 | 5.71E-09 | 5.66E-07 |
| C12orf44 | -1.025787565 | 8.89479784 | -8.83838398 | 5.99E-09 | 5.83E-07 |
| HSPB8 | -1.701768961 | 9.307919628 | -8.804754557 | 6.42E-09 | 6.18E-07 |
| LOC653506 | 1.47843004 | 10.50658537 | 8.801413675 | 6.46E-09 | 6.21E-07 |
| C5orf13 | 2.096883417 | 9.474689814 | 8.786965015 | 6.66E-09 | 6.37E-07 |
| LOC613037 | -1.036158645 | 9.893530393 | -8.769464822 | 6.91E-09 | 6.53E-07 |
| GOT1 | -1.326573629 | 9.595067245 | -8.762542738 | 7.01E-09 | 6.61E-07 |
| CSNK1G2 | 1.049525748 | 10.05185089 | 8.756806392 | 7.09E-09 | 6.67E-07 |
| ARMCX2 | 1.009885224 | 9.919286921 | 8.749107851 | 7.21E-09 | 6.72E-07 |
| CGNL1 | 1.304621619 | 8.927859432 | 8.738370424 | 7.37E-09 | 6.83E-07 |
| LOC345041 | -1.516620289 | 9.979246333 | -8.726318356 | 7.56E-09 | 6.92E-07 |
| JUN | -1.333597992 | 9.597073709 | -8.717083719 | 7.71E-09 | 6.99E-07 |
| PPAP2B | 1.608510572 | 9.641533292 | 8.663863905 | 8.62E-09 | 7.60E-07 |
| ATP6V0D1 | -1.135725081 | 8.80859904 | -8.639832935 | 9.06E-09 | 7.91E-07 |
| FOSB | -2.813419125 | 8.687441102 | -8.639013665 | 9.08E-09 | 7.91E-07 |
| TTC3 | 1.015093214 | 9.581598505 | 8.630946211 | 9.23E-09 | 8.03E-07 |
| LOC85389 | -1.141452002 | 7.825003357 | -8.594910921 | 9.96E-09 | 8.49E-07 |
| LRIG1 | 1.078556428 | 8.692913335 | 8.553315906 | 1.09E-08 | 9.07E-07 |
| CHST15 | 1.257607779 | 8.46162666 | 8.551509751 | 1.09E-08 | 9.08E-07 |
| ZFP36 | -1.156050571 | 10.17018022 | -8.545336934 | 1.11E-08 | 9.16E-07 |
| ALB | -1.142985356 | 7.801556681 | -8.531295417 | 1.14E-08 | 9.24E-07 |
| INSIG1 | 1.292701379 | 9.744545626 | 8.530896925 | 1.14E-08 | 9.24E-07 |
| MIRLET7A1 | -1.235383675 | 7.709314646 | -8.505998824 | 1.20E-08 | 9.52E-07 |
| DCP1A | -1.049021276 | 8.032745967 | -8.502444164 | 1.21E-08 | 9.54E-07 |
| SFRS17A | -1.781218434 | 9.223584995 | -8.47807162 | 1.27E-08 | 1.00E-06 |
| PDK4 | -2.304630779 | 8.784700771 | -8.474847561 | 1.28E-08 | 1.00E-06 |
| CDK7 | -1.060893687 | 9.49281256 | -8.468624678 | 1.30E-08 | 1.01E-06 |
| RNY1 | -2.398535443 | 8.747549033 | -8.465773758 | 1.31E-08 | 1.01E-06 |
| CBX4 | -1.528588529 | 8.452205455 | -8.461091629 | 1.32E-08 | 1.02E-06 |
| SULF1 | 1.475262994 | 11.15397687 | 8.443293192 | 1.37E-08 | 1.04E-06 |
| KLF9 | 1.229552513 | 8.810421944 | 8.442292652 | 1.38E-08 | 1.04E-06 |
| RRAGC | -1.151338527 | 8.821264557 | -8.425462991 | 1.43E-08 | 1.07E-06 |
| RN5S9 | -3.809920216 | 9.625513323 | -8.404389479 | 1.49E-08 | 1.10E-06 |
| HSPE1 | -1.277620878 | 11.2166407 | -8.36864695 | 1.61E-08 | 1.18E-06 |
| FAM127A | 1.033618291 | 10.56795249 | 8.352108847 | 1.67E-08 | 1.21E-06 |
| SNHG1 | -1.645666412 | 9.051346264 | -8.330179851 | 1.75E-08 | 1.25E-06 |
| CHORDC1 | -1.294907923 | 8.19216748 | -8.329087936 | 1.75E-08 | 1.25E-06 |
| RAPH1 | 1.056931998 | 8.17246991 | 8.279697008 | 1.95E-08 | 1.36E-06 |
| BRD2 | -1.250219874 | 11.39111883 | -8.261624047 | 2.02E-08 | 1.40E-06 |
| NDN | 1.174777949 | 10.9563832 | 8.258840715 | 2.04E-08 | 1.41E-06 |
| CACYBP | -1.224027732 | 8.398128322 | -8.238836986 | 2.13E-08 | 1.46E-06 |
| DNAJC3 | -1.200438081 | 8.085741812 | -8.236435319 | 2.14E-08 | 1.46E-06 |
| RNY4 | -2.052606269 | 8.308097727 | -8.208945861 | 2.27E-08 | 1.53E-06 |
| AHNAK | 1.375827578 | 10.38485784 | 8.198214805 | 2.32E-08 | 1.55E-06 |
| TAF13 | -1.31104541 | 8.119846023 | -8.196741955 | 2.33E-08 | 1.55E-06 |
| IER5 | -2.327270661 | 9.091570616 | -8.192363642 | 2.35E-08 | 1.56E-06 |
| DNAJB1 | -3.055562563 | 9.83023926 | -8.19160134 | 2.35E-08 | 1.56E-06 |
| C8orf76 | -1.164575583 | 9.398376968 | -8.181231942 | 2.41E-08 | 1.60E-06 |
| NTN4 | 1.014681531 | 9.68112302 | 8.151016407 | 2.57E-08 | 1.68E-06 |
| POTEF | 1.020640177 | 11.23819953 | 8.104323335 | 2.84E-08 | 1.82E-06 |
| HSPD1 | -1.053800884 | 10.31321288 | -8.092429647 | 2.92E-08 | 1.86E-06 |
| AUTS2 | 2.102013612 | 10.02742914 | 8.078897812 | 3.00E-08 | 1.90E-06 |
| C16orf80 | -1.151286016 | 9.534127131 | -8.039908853 | 3.27E-08 | 2.03E-06 |
| KLF4 | -1.034852376 | 8.721496329 | -8.021219727 | 3.41E-08 | 2.11E-06 |
| PDHB | 1.457496788 | 10.34802676 | 8.004263669 | 3.54E-08 | 2.17E-06 |
| STX3 | -1.214015766 | 8.057313702 | -7.984178206 | 3.69E-08 | 2.23E-06 |
| BUD31 | -1.213956155 | 10.71625896 | -7.955374225 | 3.93E-08 | 2.34E-06 |
| EGR2 | -1.667390327 | 8.104047584 | -7.94517436 | 4.02E-08 | 2.39E-06 |
| HSPA8 | -1.085343229 | 12.47307806 | -7.931682363 | 4.14E-08 | 2.44E-06 |
| MXD1 | -1.79337721 | 8.465759908 | -7.912866185 | 4.32E-08 | 2.53E-06 |
| LDLR | 1.182209427 | 10.74289893 | 7.904672339 | 4.40E-08 | 2.57E-06 |
| MAGOH | -1.291433829 | 9.321102058 | -7.901765947 | 4.43E-08 | 2.57E-06 |
| MARCKS | 2.205103985 | 10.28740139 | 7.899699605 | 4.45E-08 | 2.57E-06 |
| SNORD52 | -2.483186647 | 8.508516824 | -7.894795481 | 4.49E-08 | 2.60E-06 |
| SNAI2 | 1.323482752 | 9.337308992 | 7.893332492 | 4.51E-08 | 2.60E-06 |
| C2orf30 | 1.161338845 | 9.750992038 | 7.85044997 | 4.96E-08 | 2.78E-06 |
| CCNL1 | -1.182355588 | 8.311850072 | -7.846409045 | 5.00E-08 | 2.80E-06 |
| FNDC3B | 1.383134046 | 9.515379699 | 7.843515696 | 5.03E-08 | 2.81E-06 |
| LOC728661 | 1.226052837 | 9.106659271 | 7.835213959 | 5.13E-08 | 2.84E-06 |
| RXRA | 1.40738779 | 8.672043275 | 7.795308925 | 5.60E-08 | 3.04E-06 |
| LOC652750 | -3.504937114 | 8.808233778 | -7.77368734 | 5.87E-08 | 3.16E-06 |
| MAP1LC3B | -1.125799663 | 9.636403409 | -7.753892755 | 6.14E-08 | 3.27E-06 |
| USPL1 | -1.857063315 | 8.684387587 | -7.750114025 | 6.19E-08 | 3.29E-06 |
| RNU6ATAC | -3.123348981 | 8.807348454 | -7.746916779 | 6.23E-08 | 3.30E-06 |
| JAZF1 | 1.028041475 | 8.587327617 | 7.746797236 | 6.23E-08 | 3.30E-06 |
| SC4MOL | 1.118637415 | 9.533870485 | 7.726282451 | 6.52E-08 | 3.41E-06 |
| TMEM181 | 1.233356452 | 9.182601749 | 7.718205716 | 6.64E-08 | 3.46E-06 |
| PDGFC | 1.096148917 | 8.701723174 | 7.705789004 | 6.83E-08 | 3.56E-06 |
| C18orf19 | -1.082555496 | 7.942531595 | -7.666824554 | 7.45E-08 | 3.83E-06 |
| NSMCE4A | 1.080236969 | 8.725019204 | 7.662271531 | 7.52E-08 | 3.86E-06 |
| RLF | -1.184943186 | 8.14390246 | -7.640844743 | 7.89E-08 | 4.02E-06 |
| RRAD | -2.08199884 | 8.167079441 | -7.590856668 | 8.82E-08 | 4.36E-06 |
| C11orf46 | 1.010169725 | 8.236274876 | 7.589451577 | 8.85E-08 | 4.36E-06 |
| MED31 | -1.132742394 | 8.224109326 | -7.587083208 | 8.90E-08 | 4.37E-06 |
| NNMT | 1.723643365 | 9.900055411 | 7.54812586 | 9.71E-08 | 4.68E-06 |
| C7orf53 | -1.570784162 | 7.917170234 | -7.523295764 | 1.03E-07 | 4.88E-06 |
| PDPN | 1.401336772 | 9.70148116 | 7.522219262 | 1.03E-07 | 4.88E-06 |
| SERPINC1 | -1.329310574 | 7.715415701 | -7.520002786 | 1.03E-07 | 4.90E-06 |
| CYP1B1 | 2.050037294 | 11.75780169 | 7.502432923 | 1.08E-07 | 5.05E-06 |
| SEPHS2 | -1.181141601 | 9.117155884 | -7.501719032 | 1.08E-07 | 5.05E-06 |
| NFIX | 1.140864312 | 10.88886385 | 7.478176673 | 1.14E-07 | 5.26E-06 |
| AK1 | 1.009665192 | 10.03297628 | 7.471348857 | 1.15E-07 | 5.32E-06 |
| SYNC1 | 1.209567376 | 8.370843325 | 7.45873938 | 1.19E-07 | 5.46E-06 |
| HMOX1 | -2.320951707 | 11.84690744 | -7.448269429 | 1.22E-07 | 5.56E-06 |
| PAFAH1B1 | 1.411028999 | 8.888457384 | 7.433949188 | 1.26E-07 | 5.71E-06 |
| DHDH | -1.55743104 | 7.891944361 | -7.42713747 | 1.28E-07 | 5.77E-06 |
| LDHA | 1.039217636 | 11.65183874 | 7.402888072 | 1.35E-07 | 6.01E-06 |
| HSPA4 | -1.088079001 | 9.122800618 | -7.402472949 | 1.35E-07 | 6.01E-06 |
| SNORD3D | -2.450645351 | 8.728430982 | -7.382173907 | 1.41E-07 | 6.23E-06 |
| XYLT2 | 1.018758061 | 8.794647549 | 7.36874577 | 1.46E-07 | 6.36E-06 |
| USP36 | -1.132323889 | 7.869586227 | -7.355250038 | 1.50E-07 | 6.53E-06 |
| SERTAD1 | -1.227364638 | 9.542113567 | -7.346369484 | 1.53E-07 | 6.64E-06 |
| MPHOSPH10 | -1.317233384 | 8.947708895 | -7.342518593 | 1.55E-07 | 6.68E-06 |
| GLTP | 1.212196167 | 11.01169236 | 7.336220227 | 1.57E-07 | 6.74E-06 |
| GPSM1 | 1.012931965 | 8.943681453 | 7.319505935 | 1.63E-07 | 6.91E-06 |
| GADD45B | -2.57204562 | 10.17700896 | -7.313923702 | 1.65E-07 | 6.97E-06 |
| CYR61 | 1.580273808 | 12.04222192 | 7.296599671 | 1.72E-07 | 7.14E-06 |
| ANGPTL4 | -1.161916254 | 8.395492562 | -7.292911071 | 1.73E-07 | 7.19E-06 |
| CDK5R1 | -1.436564591 | 7.882577895 | -7.281326601 | 1.78E-07 | 7.35E-06 |
| ADAMTS1 | 1.694091485 | 10.85936734 | 7.254065528 | 1.89E-07 | 7.72E-06 |
| TDG | -1.387368593 | 8.800547092 | -7.253687737 | 1.89E-07 | 7.72E-06 |
| HES4 | -2.090636584 | 9.088291098 | -7.23587189 | 1.97E-07 | 7.97E-06 |
| PDGFRA | 1.904126582 | 10.09978518 | 7.22141938 | 2.04E-07 | 8.18E-06 |
| CCDC6 | 1.021743915 | 10.04984916 | 7.2189419 | 2.05E-07 | 8.20E-06 |
| CTDSP2 | 1.407623992 | 9.817364545 | 7.20322532 | 2.12E-07 | 8.41E-06 |
| BRF2 | -1.203473917 | 8.18540521 | -7.161307661 | 2.34E-07 | 9.07E-06 |
| AP3B1 | 1.04128459 | 9.608984539 | 7.155661166 | 2.37E-07 | 9.16E-06 |
| VCAM1 | 1.919669471 | 10.8539823 | 7.150884641 | 2.39E-07 | 9.23E-06 |
| SNIP1 | -1.815389537 | 9.009548514 | -7.134534602 | 2.49E-07 | 9.52E-06 |
| PARP9 | 1.025156991 | 8.46240179 | 7.113692897 | 2.61E-07 | 9.86E-06 |
| LOC730316 | -1.211328608 | 9.893387008 | -7.112401846 | 2.62E-07 | 9.88E-06 |
| SUPV3L1 | -1.166998357 | 8.059994167 | -7.107621795 | 2.64E-07 | 9.94E-06 |
| COL5A2 | 1.759204565 | 11.01789336 | 7.104969565 | 2.66E-07 | 9.94E-06 |
| DUSP1 | -1.365787299 | 11.00768936 | -7.086967658 | 2.77E-07 | 1.02E-05 |
| MXRA5 | 1.969141884 | 10.72845165 | 7.067505626 | 2.90E-07 | 1.06E-05 |
| RN7SK | -2.538475684 | 8.923415929 | -7.053143075 | 3.00E-07 | 1.08E-05 |
| TCP1 | -1.383944002 | 9.619489431 | -7.02514777 | 3.20E-07 | 1.14E-05 |
| GALK1 | 1.014707172 | 9.237403365 | 6.964587344 | 3.68E-07 | 1.28E-05 |
| SNORA41 | -1.274213681 | 7.81629488 | -6.950508541 | 3.80E-07 | 1.31E-05 |
| LOC727895 | -1.404331269 | 7.843961618 | -6.945292307 | 3.85E-07 | 1.32E-05 |
| ACTB | 1.04057172 | 12.57782875 | 6.940423899 | 3.89E-07 | 1.34E-05 |
| SCD | 1.312815344 | 11.28481524 | 6.904113924 | 4.24E-07 | 1.43E-05 |
| CYTSA | 1.018826884 | 8.728348163 | 6.90094923 | 4.27E-07 | 1.44E-05 |
| MED30 | -1.018616687 | 8.824399161 | -6.869801409 | 4.59E-07 | 1.52E-05 |
| CLK1 | -1.617382704 | 9.610933618 | -6.869106914 | 4.60E-07 | 1.52E-05 |
| LOXL4 | 1.477651231 | 10.58273303 | 6.859481799 | 4.70E-07 | 1.55E-05 |
| NP | -1.103440373 | 8.87044331 | -6.854533641 | 4.76E-07 | 1.57E-05 |
| PRSS23 | 1.223670315 | 10.77513739 | 6.852704677 | 4.78E-07 | 1.57E-05 |
| CENPB | 1.384445772 | 10.28667107 | 6.843309898 | 4.88E-07 | 1.60E-05 |
| DNHD2 | -1.171394927 | 7.611923225 | -6.83120274 | 5.02E-07 | 1.64E-05 |
| KIAA0367 | 1.278527551 | 9.3584855 | 6.827756561 | 5.06E-07 | 1.65E-05 |
| MALL | 1.184296271 | 10.48978798 | 6.81376936 | 5.23E-07 | 1.69E-05 |
| LPAR1 | 1.152681412 | 9.118532649 | 6.809023507 | 5.29E-07 | 1.70E-05 |
| LOC401397 | 1.183747935 | 9.959382744 | 6.803644284 | 5.36E-07 | 1.72E-05 |
| ANKRD10 | -1.239640966 | 8.497807605 | -6.801021629 | 5.39E-07 | 1.72E-05 |
| GCM1 | -1.222332029 | 7.849329402 | -6.786610834 | 5.57E-07 | 1.77E-05 |
| M6PRBP1 | 1.019670017 | 11.88481073 | 6.77139947 | 5.78E-07 | 1.83E-05 |
| STAT2 | 1.140319535 | 10.02914305 | 6.754053235 | 6.02E-07 | 1.89E-05 |
| BCAT1 | 1.387445722 | 9.896073856 | 6.741004583 | 6.20E-07 | 1.93E-05 |
| STK39 | 1.04243879 | 9.027080402 | 6.735152253 | 6.29E-07 | 1.95E-05 |
| PRRX1 | 1.519636097 | 9.491881579 | 6.731647356 | 6.34E-07 | 1.95E-05 |
| KIAA1370 | -1.41247001 | 8.556248486 | -6.703490161 | 6.78E-07 | 2.06E-05 |
| HIST1H4C | 1.305378062 | 11.95572057 | 6.660491778 | 7.50E-07 | 2.22E-05 |
| MSX1 | -1.640031064 | 8.990211713 | -6.655881129 | 7.58E-07 | 2.24E-05 |
| FNBP4 | -1.07712865 | 9.098304569 | -6.636977879 | 7.93E-07 | 2.32E-05 |
| THBS1 | 1.589834619 | 11.56692434 | 6.630278551 | 8.05E-07 | 2.34E-05 |
| DNAJA4 | -1.71678128 | 8.587413388 | -6.629655865 | 8.07E-07 | 2.35E-05 |
| SNORD3A | -2.101733336 | 8.496771676 | -6.627342188 | 8.11E-07 | 2.35E-05 |
| DAB2 | 1.49432007 | 10.37734996 | 6.624539534 | 8.16E-07 | 2.36E-05 |
| EFR3A | 1.06811111 | 8.597273265 | 6.577482303 | 9.13E-07 | 2.58E-05 |
| RBM14 | -1.043837288 | 9.598641916 | -6.56657598 | 9.36E-07 | 2.64E-05 |
| COL3A1 | 1.955286842 | 11.58270838 | 6.532647763 | 1.01E-06 | 2.82E-05 |
| SYNCRIP | 1.029937013 | 9.2483714 | 6.529733265 | 1.02E-06 | 2.83E-05 |
| ACAT2 | 1.011815616 | 8.863504087 | 6.51243819 | 1.06E-06 | 2.93E-05 |
| ELL2 | 1.062125182 | 9.796853245 | 6.509630497 | 1.07E-06 | 2.95E-05 |
| KIAA1683 | -1.321887796 | 7.934895543 | -6.497149404 | 1.10E-06 | 3.02E-05 |
| SOD2 | -1.240625687 | 10.12926395 | -6.491446823 | 1.12E-06 | 3.06E-05 |
| SLC2A3 | -1.481117996 | 9.839627199 | -6.477294768 | 1.16E-06 | 3.15E-05 |
| PRIC285 | 1.035541674 | 8.276189783 | 6.456134015 | 1.22E-06 | 3.28E-05 |
| CPNE3 | 1.010781682 | 9.723515516 | 6.43973599 | 1.27E-06 | 3.39E-05 |
| RSRC2 | -1.151344463 | 9.271064527 | -6.426888583 | 1.31E-06 | 3.48E-05 |
| ZBTB43 | -1.062313817 | 7.987457819 | -6.419041566 | 1.33E-06 | 3.53E-05 |
| COBLL1 | 1.11725727 | 9.579640739 | 6.398163607 | 1.40E-06 | 3.66E-05 |
| CD14 | 1.026341942 | 9.904401814 | 6.387010598 | 1.44E-06 | 3.74E-05 |
| LOC646197 | -2.148813879 | 9.167848513 | -6.375550777 | 1.48E-06 | 3.81E-05 |
| TRK1 | -1.138188571 | 8.030747509 | -6.368476525 | 1.50E-06 | 3.85E-05 |
| DUSP2 | -1.221839798 | 7.790837893 | -6.367487748 | 1.50E-06 | 3.85E-05 |
| RRAGA | 1.357539378 | 10.66674711 | 6.352828941 | 1.56E-06 | 3.95E-05 |
| MRPS6 | 1.124202616 | 12.48515166 | 6.35269588 | 1.56E-06 | 3.95E-05 |
| MIR886 | -2.559721657 | 8.550769098 | -6.34750203 | 1.58E-06 | 3.98E-05 |
| YWHAB | 1.081024429 | 10.25159036 | 6.265069148 | 1.92E-06 | 4.66E-05 |
| CCDC49 | -1.072621287 | 8.200454545 | -6.260937329 | 1.94E-06 | 4.70E-05 |
| PITX1 | 1.162986522 | 11.8701166 | 6.247009495 | 2.01E-06 | 4.82E-05 |
| POM121C | -1.131649814 | 9.815355972 | -6.236972618 | 2.06E-06 | 4.91E-05 |
| GREM1 | 1.668046374 | 10.11879241 | 6.213780345 | 2.18E-06 | 5.14E-05 |
| C1orf52 | -1.089131786 | 9.185619103 | -6.212891779 | 2.18E-06 | 5.15E-05 |
| HOXD1 | -1.223216254 | 7.676947719 | -6.191109866 | 2.30E-06 | 5.37E-05 |
| CEBPD | 1.437339671 | 10.44442605 | 6.174371984 | 2.39E-06 | 5.55E-05 |
| SNORA63 | -1.074961629 | 7.779642717 | -6.163796587 | 2.46E-06 | 5.66E-05 |
| CREB3L2 | 1.070785472 | 9.814531146 | 6.146630087 | 2.56E-06 | 5.86E-05 |
| EIF2AK3 | -1.079027676 | 8.147769564 | -6.081251733 | 3.00E-06 | 6.65E-05 |
| TSPYL1 | 1.129130866 | 8.960597198 | 6.078088579 | 3.02E-06 | 6.69E-05 |
| RGS16 | -1.369453491 | 8.274696623 | -6.048019645 | 3.25E-06 | 7.10E-05 |
| LOC650832 | -1.21989184 | 9.203094176 | -6.047532153 | 3.25E-06 | 7.10E-05 |
| KLHL9 | 1.791846421 | 8.869950336 | 6.021798449 | 3.46E-06 | 7.46E-05 |
| NKX3-1 | -1.400178162 | 8.916801613 | -6.004694335 | 3.61E-06 | 7.69E-05 |
| UCHL1 | -1.115310128 | 8.960687621 | -5.948682299 | 4.14E-06 | 8.61E-05 |
| LMCD1 | 1.43274614 | 9.866743621 | 5.930486851 | 4.33E-06 | 8.91E-05 |
| LOC643310 | -1.084027379 | 10.00954927 | -5.87708515 | 4.93E-06 | 9.96E-05 |
| LOC151579 | 1.008709654 | 11.32732394 | 5.866510679 | 5.06E-06 | 0.000101991 |
| HSPA14 | -1.013286747 | 8.435287897 | -5.86148077 | 5.12E-06 | 0.000102923 |
| MIR21 | -1.079025207 | 7.788827609 | -5.844079808 | 5.35E-06 | 0.000106446 |
| C9orf152 | -1.013290095 | 7.547356721 | -5.820458116 | 5.66E-06 | 0.000110886 |
| HNRPDL | -1.112825131 | 8.77520246 | -5.730857169 | 7.06E-06 | 0.000132622 |
| FASTKD5 | -1.011672386 | 8.302962687 | -5.669418014 | 8.21E-06 | 0.000149968 |
| PARP4 | 1.097054998 | 9.296526955 | 5.625377235 | 9.16E-06 | 0.000163838 |
| SRXN1 | -1.521139057 | 8.902046836 | -5.602762976 | 9.68E-06 | 0.000171671 |
| EGR1 | -1.408141509 | 9.993085136 | -5.576829698 | 1.03E-05 | 0.000181 |
| RNY5 | -1.620698585 | 8.082082134 | -5.575284244 | 1.04E-05 | 0.00018149 |
| ZMIZ1 | 1.554008474 | 10.10525891 | 5.57421797 | 1.04E-05 | 0.000181567 |
| IGFBP5 | 2.037818023 | 10.85634763 | 5.568122052 | 1.05E-05 | 0.000183911 |
| EAF1 | -1.411672935 | 8.726943218 | -5.557772087 | 1.08E-05 | 0.000187349 |
| THBS2 | 1.427847306 | 9.018778395 | 5.539694718 | 1.13E-05 | 0.000194396 |
| PALLD | 1.022646451 | 10.27674088 | 5.532061695 | 1.15E-05 | 0.000197148 |
| SPTY2D1 | -1.049485596 | 8.074069949 | -5.5025584 | 1.24E-05 | 0.000208333 |
| FAM129B | 1.110318348 | 9.923837399 | 5.491383148 | 1.28E-05 | 0.000213273 |
| PPP1R3C | 2.020701879 | 11.28325392 | 5.488703104 | 1.28E-05 | 0.000214467 |
| ARPC5 | 1.20528555 | 10.72720338 | 5.446615302 | 1.43E-05 | 0.000233845 |
| IMP3 | 1.467919805 | 9.612252181 | 5.415374724 | 1.54E-05 | 0.000249199 |
| C7orf30 | 1.156615891 | 10.01893304 | 5.339379227 | 1.86E-05 | 0.000289448 |
| PSMD14 | -1.208049168 | 9.961530284 | -5.282551541 | 2.14E-05 | 0.000326048 |
| HMGB2 | 1.14935462 | 9.423975668 | 5.273701529 | 2.19E-05 | 0.00033124 |
| HERPUD1 | -1.548204788 | 10.29441214 | -5.19408576 | 2.68E-05 | 0.000387158 |
| RND3 | -1.664501 | 10.60337026 | -5.171154968 | 2.83E-05 | 0.000405523 |
| HPS6 | 1.191114404 | 8.605886269 | 5.168453793 | 2.85E-05 | 0.000407715 |
| SGK1 | -1.244877736 | 10.06579933 | -5.161922479 | 2.90E-05 | 0.00041199 |
| OKL38 | -1.958104459 | 9.655967258 | -5.154980339 | 2.95E-05 | 0.000417044 |
| TUFT1 | -1.064468171 | 8.728214476 | -5.148568079 | 3.00E-05 | 0.000422547 |
| DUSP6 | -1.074625221 | 8.320868284 | -5.13664411 | 3.09E-05 | 0.000432436 |
| IER3 | -1.250429019 | 10.82651098 | -5.134736858 | 3.10E-05 | 0.000433668 |
| FAM43A | -1.045410794 | 8.33540436 | -5.133139555 | 3.12E-05 | 0.000434501 |
| SNORD3C | -1.37976401 | 7.853396142 | -5.130771348 | 3.14E-05 | 0.000436309 |
| FAM53C | -1.113668885 | 9.11984177 | -5.058246235 | 3.76E-05 | 0.000508181 |
| SLC26A4 | 1.085388098 | 8.30418386 | 5.002133576 | 4.33E-05 | 0.000568373 |
| PTGS2 | -1.417053317 | 9.560270607 | -4.98242117 | 4.55E-05 | 0.000592509 |
| LOC100129668 | -1.032969859 | 9.043132703 | -4.978251977 | 4.60E-05 | 0.000597754 |
| PPIC | 1.01503833 | 10.3641663 | 4.966212404 | 4.74E-05 | 0.000612809 |
| ZBED5 | 1.089406409 | 8.620607839 | 4.945142693 | 5.00E-05 | 0.000639538 |
| CPA4 | 1.204573159 | 8.462758455 | 4.885810064 | 5.80E-05 | 0.000725285 |
| SGK | -1.145007932 | 10.68602429 | -4.840746306 | 6.50E-05 | 0.000795887 |
| SNORA20 | -1.491682773 | 7.819998574 | -4.809601601 | 7.03E-05 | 0.000848575 |
| MAP3K8 | -1.061184039 | 8.384243546 | -4.800068199 | 7.20E-05 | 0.000865176 |
| FADD | 1.086679234 | 8.291866832 | 4.797401681 | 7.25E-05 | 0.000870007 |
| COL1A1 | 2.228976709 | 11.10118636 | 4.748242734 | 8.20E-05 | 0.00096442 |
| IFIT1 | 1.388959718 | 8.778808054 | 4.734214258 | 8.50E-05 | 0.000993915 |
| HSPA5 | -1.628544187 | 9.895254241 | -4.599454256 | 0.000119382 | 0.00130849 |
| RCAN2 | 1.143318995 | 8.694987541 | 4.591732202 | 0.000121731 | 0.001329442 |
| COL1A2 | 1.726928576 | 11.93808274 | 4.539758765 | 0.000138795 | 0.001486961 |
| RRAS | 1.006789875 | 10.09179116 | 4.533359881 | 0.000141055 | 0.001507862 |
| GCLM | -1.102418933 | 9.135085666 | -4.53084116 | 0.000141955 | 0.001516443 |
| BTG1 | 1.233791365 | 12.28598812 | 4.452132216 | 0.000173155 | 0.001783402 |
| ANKRD1 | -1.243567245 | 7.879860946 | -4.40114375 | 0.000196937 | 0.001966853 |
| SPRED1 | 1.069851094 | 9.39141774 | 4.391912768 | 0.000201578 | 0.002003621 |
| STAT1 | 1.095912661 | 10.24010098 | 4.377809735 | 0.000208882 | 0.002061243 |
| MYLIP | -1.113536824 | 9.124214517 | -4.314542033 | 0.000245032 | 0.002351165 |
| DHRS3 | 1.057891544 | 9.889533732 | 4.309632056 | 0.000248085 | 0.002373494 |
| CCL2 | 1.323514014 | 10.22395529 | 4.262050366 | 0.000279712 | 0.002614205 |
| PAPPA | 1.005926442 | 8.650905836 | 4.208497704 | 0.00032013 | 0.002915352 |
| FKSG30 | 1.095146189 | 10.11764649 | 4.202101127 | 0.000325331 | 0.002958821 |
| FZD9 | 1.063497803 | 9.400055418 | 4.194816931 | 0.000331355 | 0.003003597 |
| MT1G | -1.384673367 | 10.26552146 | -4.175619245 | 0.00034777 | 0.003123446 |
| COL12A1 | 1.179898005 | 10.18544694 | 4.153820483 | 0.000367391 | 0.003265003 |
| FLNC | -1.035539357 | 8.197765236 | -4.140202993 | 0.000380202 | 0.003360726 |
| RPESP | 1.046816904 | 9.518788764 | 4.05957128 | 0.000465673 | 0.003980522 |
| FZD8 | 1.095674794 | 8.721317637 | 4.048235353 | 0.000479127 | 0.004072035 |
| METTL7A | 1.073324936 | 9.47596662 | 4.036519514 | 0.000493437 | 0.004171958 |
| CDH11 | 1.208618469 | 10.40688351 | 4.01841798 | 0.000516384 | 0.004319298 |
| FBXO30 | -1.154835852 | 8.50162886 | -3.971094942 | 0.000581486 | 0.004763288 |
| LOC100132728 | -1.091422279 | 8.063606798 | -3.741541126 | 0.001031907 | 0.007626609 |
| COL11A1 | 1.191665969 | 9.906899049 | 3.608117415 | 0.001436858 | 0.009973639 |
| RGS2 | -1.191710039 | 10.00964095 | -3.28917129 | 0.003139798 | 0.019030922 |
| UAP1 | 1.004509362 | 10.09679437 | 3.087947548 | 0.00509494 | 0.028225617 |
| KIAA1199 | 1.115801888 | 11.29322666 | 3.033170537 | 0.005804006 | 0.031348644 |
| MX1 | 1.050041451 | 10.89647078 | 3.031840934 | 0.005822342 | 0.031436832 |
| LOC400750 | -1.015870125 | 8.280929454 | -3.031652764 | 0.005824942 | 0.031445444 |
| ACTA2 | 1.118993858 | 10.25601217 | 2.920755583 | 0.007566551 | 0.039039438 |
| TXNL1 | -1.213673168 | 9.401440322 | -2.885646865 | 0.008214588 | 0.041651808 |

TABLE S8 Significantly different immune cells in GSE66360 and GSE75181.

| GSE66360 | |  | GSE75181 | |
| --- | --- | --- | --- | --- |
| Immune cells | p-value |  | Immune cells | p-value |
| Plasma cells | 0.036056451 |  | Plasma cells | 0.000585541 |
| T cells CD8 | 0.007700052 |  | T cells CD4 memory resting | 0.006811737 |
| T cells CD4 memory resting | 3.31E-06 |  | T cells follicular helper | 0.013721331 |
| T cells follicular helper | 0.000273746 |  |  |  |
| T cells regulatory (Tregs) | 0.003403605 |  |  |  |
| T cells gamma delta | 0.001504321 |  |  |  |
| NK cells resting | 0.008214084 |  |  |  |
| NK cells activated | 0.005767538 |  |  |  |
| Monocytes | 0.000209686 |  |  |  |
| Macrophages M0 | 0.032325435 |  |  |  |
| Dendritic cells activated | 0.000257161 |  |  |  |
| Mast cells resting | 0.000654469 |  |  |  |
| Mast cells activated | 5.94E-11 |  |  |  |
| Neutrophils | 1.96E-09 |  |  |  |

TABLE S9 Binding sites of transcription factor FOXC1 to three hub genes.

| Matrix ID | Name | Score | Relative score | Sequence ID | Species | Start | End | Strand | Predicted sequence |
| --- | --- | --- | --- | --- | --- | --- | --- | --- | --- |
| MA0032.1 | MA0032.1.FOXC1 | 6.763779 | 0.960406357 | DUSP1 | Homo sapiens | 966 | 973 | + | CCTGAGTA |
| MA0032.2 | MA0032.2.FOXC1 | 13.705637 | 0.959226672 | DUSP1 | Homo sapiens | 2535 | 2545 | - | TAAATAAATAA |
| MA0032.2 | MA0032.2.FOXC1 | 12.809513 | 0.945774938 | DUSP1 | Homo sapiens | 2803 | 2813 | - | AAAATAAATAA |
| MA0032.1 | MA0032.1.FOXC1 | 6.138175 | 0.925411632 | DUSP1 | Homo sapiens | 2031 | 2038 | + | CTCAAGTA |
| MA0032.1 | MA0032.1.FOXC1 | 6.056561 | 0.920846348 | DUSP1 | Homo sapiens | 2754 | 2761 | - | GGTAGGTA |
| MA0032.2 | MA0032.2.FOXC1 | 11.145472 | 0.920795978 | DUSP1 | Homo sapiens | 1261 | 1271 | - | TAAATAAATAG |
| MA0032.1 | MA0032.1.FOXC1 | 5.9861717 | 0.916908949 | DUSP1 | Homo sapiens | 1441 | 1448 | + | AGTGCGTA |
| MA0032.1 | MA0032.1.FOXC1 | 5.9157825 | 0.91297155 | DUSP1 | Homo sapiens | 1730 | 1737 | + | ATTCAGTA |
| MA0032.1 | MA0032.1.FOXC1 | 5.9157825 | 0.91297155 | DUSP1 | Homo sapiens | 2889 | 2896 | - | GTACAGTA |
| MA0032.1 | MA0032.1.FOXC1 | 5.856889 | 0.909677186 | DUSP1 | Homo sapiens | 2883 | 2890 | + | TTTGAGTA |
| MA0032.1 | MA0032.1.FOXC1 | 5.822673 | 0.907763232 | DUSP1 | Homo sapiens | 1994 | 2001 | - | ACAGTGTA |
| MA0032.1 | MA0032.1.FOXC1 | 5.763779 | 0.904468868 | DUSP1 | Homo sapiens | 1087 | 1094 | + | GAAGAGTA |
| MA0032.1 | MA0032.1.FOXC1 | 5.7231374 | 0.902195468 | DUSP1 | Homo sapiens | 791 | 798 | + | CCGATGTA |
| MA0032.1 | MA0032.1.FOXC1 | 5.6642437 | 0.898901104 | DUSP1 | Homo sapiens | 1035 | 1042 | + | GGTTAGTA |
| MA0032.1 | MA0032.1.FOXC1 | 5.5938544 | 0.894963705 | DUSP1 | Homo sapiens | 717 | 724 | + | CTGGAGTA |
| MA0032.2 | MA0032.2.FOXC1 | 9.346194 | 0.893786991 | DUSP1 | Homo sapiens | 1265 | 1275 | - | AGGATAAATAA |
| MA0032.1 | MA0032.1.FOXC1 | 5.571134 | 0.893692786 | DUSP1 | Homo sapiens | 2640 | 2647 | + | AGTGGGTA |
| MA0032.1 | MA0032.1.FOXC1 | 5.5596385 | 0.893049752 | DUSP1 | Homo sapiens | 1995 | 2002 | + | ACACTGTA |
| MA0032.1 | MA0032.1.FOXC1 | 5.500745 | 0.889755387 | DUSP1 | Homo sapiens | 1922 | 1929 | + | GAACAGTA |
| MA0032.1 | MA0032.1.FOXC1 | 5.4012094 | 0.884187623 | DUSP1 | Homo sapiens | 612 | 619 | - | AGGGCGTA |
| MA0032.1 | MA0032.1.FOXC1 | 5.4012094 | 0.884187623 | DUSP1 | Homo sapiens | 1061 | 1068 | + | AAGAAGTA |
| MA0032.1 | MA0032.1.FOXC1 | 5.4012094 | 0.884187623 | DUSP1 | Homo sapiens | 2894 | 2901 | - | GGATAGTA |
| MA0032.2 | MA0032.2.FOXC1 | 8.6495495 | 0.883329642 | DUSP1 | Homo sapiens | 1951 | 1961 | - | ATGGTCAACAT |
| MA0032.1 | MA0032.1.FOXC1 | 5.33082 | 0.880250224 | DUSP1 | Homo sapiens | 1011 | 1018 | + | CTGCAGTA |
| MA0032.1 | MA0032.1.FOXC1 | 5.3080997 | 0.878979305 | DUSP1 | Homo sapiens | 1432 | 1439 | - | CCCAGGTA |
| MA0032.1 | MA0032.1.FOXC1 | 5.3080997 | 0.878979305 | DUSP1 | Homo sapiens | 2001 | 2008 | - | CCCAGGTA |
| MA0032.1 | MA0032.1.FOXC1 | 5.3080997 | 0.878979305 | DUSP1 | Homo sapiens | 2785 | 2792 | - | CATATGTA |
| MA0032.2 | MA0032.2.FOXC1 | 8.285909 | 0.877871023 | DUSP1 | Homo sapiens | 2900 | 2910 | + | CCTGTAAATAT |
| MA0032.1 | MA0032.1.FOXC1 | 5.249206 | 0.875684941 | DUSP1 | Homo sapiens | 1335 | 1342 | + | TGTGGGTA |
| MA0032.1 | MA0032.1.FOXC1 | 5.2377105 | 0.875041906 | DUSP1 | Homo sapiens | 2888 | 2895 | + | GTACTGTA |
| MA0032.1 | MA0032.1.FOXC1 | 5.2085643 | 0.873411541 | DUSP1 | Homo sapiens | 2396 | 2403 | - | TGAAGGTA |
| MA0032.1 | MA0032.1.FOXC1 | 5.178817 | 0.871747542 | DUSP1 | Homo sapiens | 2812 | 2819 | + | TTTGTGTA |
| MA0032.1 | MA0032.1.FOXC1 | 5.138175 | 0.869474142 | DUSP1 | Homo sapiens | 972 | 979 | - | CGCTAGTA |
| MA0032.1 | MA0032.1.FOXC1 | 5.0857077 | 0.866539251 | DUSP1 | Homo sapiens | 659 | 666 | + | GCCCGGTA |
| MA0032.1 | MA0032.1.FOXC1 | 5.0857077 | 0.866539251 | DUSP1 | Homo sapiens | 1296 | 1303 | + | ACTCGGTA |
| MA0032.2 | MA0032.2.FOXC1 | 7.420205 | 0.86487593 | DUSP1 | Homo sapiens | 1308 | 1318 | - | AAAGTCAATTT |
| MA0032.1 | MA0032.1.FOXC1 | 4.974676 | 0.860328425 | DUSP1 | Homo sapiens | 2898 | 2905 | + | ATCCTGTA |
| MA0032.1 | MA0032.1.FOXC1 | 4.9157825 | 0.857034061 | DUSP1 | Homo sapiens | 1426 | 1433 | + | TTTCTGTA |
| MA0032.1 | MA0032.1.FOXC1 | 4.9157825 | 0.857034061 | DUSP1 | Homo sapiens | 1540 | 1547 | + | TACCAGTA |
| MA0032.1 | MA0032.1.FOXC1 | 4.9157825 | 0.857034061 | DUSP1 | Homo sapiens | 2758 | 2765 | + | TACCAGTA |
| MA0032.1 | MA0032.1.FOXC1 | 4.856889 | 0.853739696 | DUSP1 | Homo sapiens | 2848 | 2855 | - | TAGGAGTA |
| MA0032.1 | MA0032.1.FOXC1 | 4.822673 | 0.851825743 | DUSP1 | Homo sapiens | 1795 | 1802 | - | AATCTGTA |
| MA0032.1 | MA0032.1.FOXC1 | 4.816247 | 0.851466297 | DUSP1 | Homo sapiens | 2646 | 2653 | - | TTGATGTA |
| MA0032.1 | MA0032.1.FOXC1 | 4.7231374 | 0.846257979 | DUSP1 | Homo sapiens | 723 | 730 | - | TAAATGTA |
| MA0032.1 | MA0032.1.FOXC1 | 4.7231374 | 0.846257979 | DUSP1 | Homo sapiens | 2144 | 2151 | - | ATAAGGTA |
| MA0032.1 | MA0032.1.FOXC1 | 4.7231374 | 0.846257979 | DUSP1 | Homo sapiens | 2799 | 2806 | - | ATAAGGTA |
| MA0032.1 | MA0032.1.FOXC1 | 4.652748 | 0.84232058 | DUSP1 | Homo sapiens | 1758 | 1765 | + | TTCCTGTA |
| MA0032.2 | MA0032.2.FOXC1 | 5.7884502 | 0.840381625 | DUSP1 | Homo sapiens | 762 | 772 | + | TCTGGAAACAA |
| MA0032.2 | MA0032.2.FOXC1 | 5.7425056 | 0.839691948 | DUSP1 | Homo sapiens | 2139 | 2149 | - | AAGGTAAGCAA |
| MA0032.1 | MA0032.1.FOXC1 | 4.5938544 | 0.839026215 | DUSP1 | Homo sapiens | 1213 | 1220 | - | TTTCCGTA |
| MA0032.2 | MA0032.2.FOXC1 | 5.683998 | 0.838813692 | DUSP1 | Homo sapiens | 2789 | 2799 | + | TATGAGAATAT |
| MA0032.2 | MA0032.2.FOXC1 | 5.639356 | 0.83814357 | DUSP1 | Homo sapiens | 1129 | 1139 | - | GTTATAAATAA |
| MA0032.2 | MA0032.2.FOXC1 | 5.631629 | 0.838027578 | DUSP1 | Homo sapiens | 1619 | 1629 | + | TAGGTAAATGG |
| MA0032.1 | MA0032.1.FOXC1 | 4.571134 | 0.837755297 | DUSP1 | Homo sapiens | 1617 | 1624 | + | CATAGGTA |
| MA0032.2 | MA0032.2.FOXC1 | 5.456321 | 0.835396023 | DUSP1 | Homo sapiens | 370 | 380 | + | TCTGTCAACGT |
| MA0032.2 | MA0032.2.FOXC1 | 5.443676 | 0.835206212 | DUSP1 | Homo sapiens | 865 | 875 | + | AAAAAAAATGT |
| MA0032.2 | MA0032.2.FOXC1 | 5.295287 | 0.832978744 | DUSP1 | Homo sapiens | 2539 | 2549 | - | AAGTTAAATAA |
| MA0032.1 | MA0032.1.FOXC1 | 4.441851 | 0.830523533 | DUSP1 | Homo sapiens | 895 | 902 | - | GCTTCGTA |
| MA0032.1 | MA0032.1.FOXC1 | 4.441851 | 0.830523533 | DUSP1 | Homo sapiens | 1017 | 1024 | - | GTGGGGTA |
| MA0032.2 | MA0032.2.FOXC1 | 4.9756484 | 0.82818064 | DUSP1 | Homo sapiens | 2807 | 2817 | - | CACAAAAATAA |
| MA0032.2 | MA0032.2.FOXC1 | 4.724948 | 0.824417371 | DUSP1 | Homo sapiens | 793 | 803 | + | GATGTAAACTT |
| MA0032.2 | MA0032.2.FOXC1 | 4.7069283 | 0.824146877 | DUSP1 | Homo sapiens | 2584 | 2594 | - | TAAGTCATCAC |
| MA0032.1 | MA0032.1.FOXC1 | 4.3080997 | 0.823041816 | DUSP1 | Homo sapiens | 606 | 613 | + | CAAAGGTA |
| MA0032.2 | MA0032.2.FOXC1 | 4.5615506 | 0.82196461 | DUSP1 | Homo sapiens | 2090 | 2100 | - | TGGACAAACAC |
| MA0032.2 | MA0032.2.FOXC1 | 4.3131347 | 0.818235634 | DUSP1 | Homo sapiens | 2939 | 2949 | + | ATGGAAAATAC |
| MA0032.2 | MA0032.2.FOXC1 | 4.246675 | 0.817238006 | DUSP1 | Homo sapiens | 744 | 754 | - | AAAGCCAATGA |
| MA0032.2 | MA0032.2.FOXC1 | 4.211917 | 0.816716252 | DUSP1 | Homo sapiens | 2874 | 2884 | - | AAAACAAAAAT |
| MA0032.1 | MA0032.1.FOXC1 | 4.178817 | 0.815810052 | DUSP1 | Homo sapiens | 875 | 882 | + | TCTTTGTA |
| MA0032.2 | MA0032.2.FOXC1 | 4.024027 | 0.813895831 | DUSP1 | Homo sapiens | 2781 | 2791 | + | CATATACATAT |
| MA0032.1 | MA0032.1.FOXC1 | 4.0792813 | 0.810242288 | DUSP1 | Homo sapiens | 2867 | 2874 | - | TTGAGGTA |
| MA0032.2 | MA0032.2.FOXC1 | 3.4682674 | 0.805553315 | DUSP1 | Homo sapiens | 2780 | 2790 | - | TATGTATATGT |
| MA0032.2 | MA0032.2.FOXC1 | 3.20874 | 0.801657543 | DUSP1 | Homo sapiens | 2924 | 2934 | - | GCTGAAAACAA |
| MA0032.2 | MA0032.2.FOXC1 | 3.1519616 | 0.800805241 | DUSP1 | Homo sapiens | 2809 | 2819 | - | TACACAAAAAT |
| MA0032.1 | MA0032.1.FOXC1 | 6.571134 | 0.949630275 | FOS | Homo sapiens | 701 | 708 | + | GGTGTGTA |
| MA0032.1 | MA0032.1.FOXC1 | 6.500745 | 0.945692876 | FOS | Homo sapiens | 1372 | 1379 | + | GCCCAGTA |
| MA0032.1 | MA0032.1.FOXC1 | 6.500745 | 0.945692876 | FOS | Homo sapiens | 1846 | 1853 | - | ACAGAGTA |
| MA0032.1 | MA0032.1.FOXC1 | 6.4012094 | 0.940125112 | FOS | Homo sapiens | 1485 | 1492 | - | TGAGAGTA |
| MA0032.1 | MA0032.1.FOXC1 | 6.3080997 | 0.934916795 | FOS | Homo sapiens | 1828 | 1835 | - | GGAGTGTA |
| MA0032.1 | MA0032.1.FOXC1 | 6.3080997 | 0.934916795 | FOS | Homo sapiens | 2818 | 2825 | + | AGTGTGTA |
| MA0032.1 | MA0032.1.FOXC1 | 6.2377105 | 0.930979396 | FOS | Homo sapiens | 1660 | 1667 | - | CCCCAGTA |
| MA0032.1 | MA0032.1.FOXC1 | 6.178817 | 0.927685031 | FOS | Homo sapiens | 1908 | 1915 | + | CTTGAGTA |
| MA0032.1 | MA0032.1.FOXC1 | 6.0792813 | 0.922117267 | FOS | Homo sapiens | 2444 | 2451 | - | TGGGAGTA |
| MA0032.1 | MA0032.1.FOXC1 | 6.056561 | 0.920846348 | FOS | Homo sapiens | 1840 | 1847 | + | GGTAGGTA |
| MA0032.1 | MA0032.1.FOXC1 | 6.026814 | 0.919182375 | FOS | Homo sapiens | 376 | 383 | + | GATGAGTA |
| MA0032.1 | MA0032.1.FOXC1 | 5.9157825 | 0.91297155 | FOS | Homo sapiens | 1698 | 1705 | - | ATTCAGTA |
| MA0032.1 | MA0032.1.FOXC1 | 5.822673 | 0.907763232 | FOS | Homo sapiens | 1378 | 1385 | - | CCTCTGTA |
| MA0032.1 | MA0032.1.FOXC1 | 5.822673 | 0.907763232 | FOS | Homo sapiens | 1943 | 1950 | + | CCTCTGTA |
| MA0032.2 | MA0032.2.FOXC1 | 10.213778 | 0.9068103 | FOS | Homo sapiens | 2971 | 2981 | - | TTTGAAAATAT |
| MA0032.1 | MA0032.1.FOXC1 | 5.763779 | 0.904468868 | FOS | Homo sapiens | 1318 | 1325 | - | GAAGAGTA |
| MA0032.1 | MA0032.1.FOXC1 | 5.652748 | 0.898258069 | FOS | Homo sapiens | 368 | 375 | + | CTCCAGTA |
| MA0032.1 | MA0032.1.FOXC1 | 5.6117764 | 0.895966212 | FOS | Homo sapiens | 82 | 89 | - | GCTGGGTA |
| MA0032.1 | MA0032.1.FOXC1 | 5.5938544 | 0.894963705 | FOS | Homo sapiens | 1224 | 1231 | - | CTGGAGTA |
| MA0032.1 | MA0032.1.FOXC1 | 5.5304923 | 0.891419387 | FOS | Homo sapiens | 292 | 299 | + | CGCAGGTA |
| MA0032.1 | MA0032.1.FOXC1 | 5.500745 | 0.889755387 | FOS | Homo sapiens | 2657 | 2664 | - | TCTCTGTA |
| MA0032.1 | MA0032.1.FOXC1 | 5.4012094 | 0.884187623 | FOS | Homo sapiens | 1479 | 1486 | + | AAGAAGTA |
| MA0032.1 | MA0032.1.FOXC1 | 5.4012094 | 0.884187623 | FOS | Homo sapiens | 2856 | 2863 | + | AGTTAGTA |
| MA0032.1 | MA0032.1.FOXC1 | 5.0857077 | 0.866539251 | FOS | Homo sapiens | 2463 | 2470 | - | GACGTGTA |
| MA0032.1 | MA0032.1.FOXC1 | 4.9861717 | 0.86097146 | FOS | Homo sapiens | 2797 | 2804 | + | GAGATGTA |
| MA0032.1 | MA0032.1.FOXC1 | 4.974676 | 0.860328425 | FOS | Homo sapiens | 1951 | 1958 | - | CTCCTGTA |
| MA0032.1 | MA0032.1.FOXC1 | 4.9157825 | 0.857034061 | FOS | Homo sapiens | 475 | 482 | + | CCATAGTA |
| MA0032.1 | MA0032.1.FOXC1 | 4.9157825 | 0.857034061 | FOS | Homo sapiens | 1312 | 1319 | + | CTAGCGTA |
| MA0032.2 | MA0032.2.FOXC1 | 6.4506187 | 0.850321449 | FOS | Homo sapiens | 785 | 795 | - | GTTATAAATAT |
| MA0032.1 | MA0032.1.FOXC1 | 4.652748 | 0.84232058 | FOS | Homo sapiens | 2690 | 2697 | + | TTCCTGTA |
| MA0032.2 | MA0032.2.FOXC1 | 5.9072485 | 0.842164908 | FOS | Homo sapiens | 2866 | 2876 | - | TGGCTCAACAT |
| MA0032.1 | MA0032.1.FOXC1 | 4.5596385 | 0.837112262 | FOS | Homo sapiens | 2438 | 2445 | + | CACCTGTA |
| MA0032.1 | MA0032.1.FOXC1 | 4.500745 | 0.833817898 | FOS | Homo sapiens | 1413 | 1420 | + | CATCCGTA |
| MA0032.1 | MA0032.1.FOXC1 | 4.4943185 | 0.833458425 | FOS | Homo sapiens | 240 | 247 | - | TGGTAGTA |
| MA0032.1 | MA0032.1.FOXC1 | 4.441851 | 0.830523533 | FOS | Homo sapiens | 2487 | 2494 | - | TCGGGGTA |
| MA0032.2 | MA0032.2.FOXC1 | 4.2042356 | 0.816600947 | FOS | Homo sapiens | 717 | 727 | - | TTTATCAATGA |
| MA0032.1 | MA0032.1.FOXC1 | 4.178817 | 0.815810052 | FOS | Homo sapiens | 183 | 190 | - | GCCTCGTA |
| MA0032.2 | MA0032.2.FOXC1 | 3.969305 | 0.8130744 | FOS | Homo sapiens | 1609 | 1619 | - | TGTGAAACCAT |
| MA0032.2 | MA0032.2.FOXC1 | 3.6823292 | 0.8087666 | FOS | Homo sapiens | 1928 | 1938 | - | AAGGAAAGCAT |
| MA0032.1 | MA0032.1.FOXC1 | 3.9861717 | 0.80503397 | FOS | Homo sapiens | 2786 | 2793 | - | AAGAGGTA |
| MA0032.1 | MA0032.1.FOXC1 | 3.9157825 | 0.801096571 | FOS | Homo sapiens | 1514 | 1521 | - | ATGCGGTA |
| MA0032.1 | MA0032.1.FOXC1 | 7.2085643 | 0.98528652 | THBS1 | Homo sapiens | 600 | 607 | + | GGAAAGTA |
| MA0032.1 | MA0032.1.FOXC1 | 6.94553 | 0.970573039 | THBS1 | Homo sapiens | 2062 | 2069 | - | AGAAAGTA |
| MA0032.1 | MA0032.1.FOXC1 | 6.8866363 | 0.967278675 | THBS1 | Homo sapiens | 433 | 440 | - | GGGAAGTA |
| MA0032.1 | MA0032.1.FOXC1 | 6.7231374 | 0.958132958 | THBS1 | Homo sapiens | 379 | 386 | - | AGAGAGTA |
| MA0032.1 | MA0032.1.FOXC1 | 6.7231374 | 0.958132958 | THBS1 | Homo sapiens | 2294 | 2301 | + | CCCAAGTA |
| MA0032.1 | MA0032.1.FOXC1 | 6.6642437 | 0.954838593 | THBS1 | Homo sapiens | 2614 | 2621 | + | TGTGAGTA |
| MA0032.1 | MA0032.1.FOXC1 | 6.3080997 | 0.934916795 | THBS1 | Homo sapiens | 2777 | 2784 | - | CGTGTGTA |
| MA0032.1 | MA0032.1.FOXC1 | 6.178817 | 0.927685031 | THBS1 | Homo sapiens | 63 | 70 | - | CCGGAGTA |
| MA0032.1 | MA0032.1.FOXC1 | 6.178817 | 0.927685031 | THBS1 | Homo sapiens | 1933 | 1940 | - | CTTGAGTA |
| MA0032.1 | MA0032.1.FOXC1 | 6.178817 | 0.927685031 | THBS1 | Homo sapiens | 2328 | 2335 | - | CTTGAGTA |
| MA0032.1 | MA0032.1.FOXC1 | 6.0857077 | 0.92247674 | THBS1 | Homo sapiens | 2286 | 2293 | + | CCTGTGTA |
| MA0032.1 | MA0032.1.FOXC1 | 6.0450654 | 0.920203314 | THBS1 | Homo sapiens | 245 | 252 | - | AGAGTGTA |
| MA0032.1 | MA0032.1.FOXC1 | 6.0450654 | 0.920203314 | THBS1 | Homo sapiens | 427 | 434 | + | AGTCTGTA |
| MA0032.1 | MA0032.1.FOXC1 | 5.9861717 | 0.916908949 | THBS1 | Homo sapiens | 142 | 149 | - | TGTGTGTA |
| MA0032.1 | MA0032.1.FOXC1 | 5.9861717 | 0.916908949 | THBS1 | Homo sapiens | 2763 | 2770 | - | TGTGTGTA |
| MA0032.1 | MA0032.1.FOXC1 | 5.7231374 | 0.902195468 | THBS1 | Homo sapiens | 1754 | 1761 | + | TGTCTGTA |
| MA0032.1 | MA0032.1.FOXC1 | 5.7231374 | 0.902195468 | THBS1 | Homo sapiens | 1879 | 1886 | + | AACAAGTA |
| MA0032.1 | MA0032.1.FOXC1 | 5.7231374 | 0.902195468 | THBS1 | Homo sapiens | 2465 | 2472 | - | TGTCTGTA |
| MA0032.1 | MA0032.1.FOXC1 | 5.7231374 | 0.902195468 | THBS1 | Homo sapiens | 2791 | 2798 | - | TGCGTGTA |
| MA0032.2 | MA0032.2.FOXC1 | 9.852119 | 0.901381444 | THBS1 | Homo sapiens | 983 | 993 | - | TGAATAAATAC |
| MA0032.2 | MA0032.2.FOXC1 | 9.187263 | 0.891401263 | THBS1 | Homo sapiens | 2395 | 2405 | - | TATGTCAAAAT |
| MA0032.1 | MA0032.1.FOXC1 | 5.500745 | 0.889755387 | THBS1 | Homo sapiens | 2078 | 2085 | + | AATCAGTA |
| MA0032.1 | MA0032.1.FOXC1 | 5.4012094 | 0.884187623 | THBS1 | Homo sapiens | 1549 | 1556 | - | TCGATGTA |
| MA0032.1 | MA0032.1.FOXC1 | 5.4012094 | 0.884187623 | THBS1 | Homo sapiens | 2510 | 2517 | + | TACAAGTA |
| MA0032.1 | MA0032.1.FOXC1 | 5.4012094 | 0.884187623 | THBS1 | Homo sapiens | 2701 | 2708 | - | TTTATGTA |
| MA0032.1 | MA0032.1.FOXC1 | 5.2377105 | 0.875041906 | THBS1 | Homo sapiens | 1858 | 1865 | + | ATTCTGTA |
| MA0032.1 | MA0032.1.FOXC1 | 5.2377105 | 0.875041906 | THBS1 | Homo sapiens | 2737 | 2744 | - | CTTCTGTA |
| MA0032.1 | MA0032.1.FOXC1 | 5.2085643 | 0.873411541 | THBS1 | Homo sapiens | 2806 | 2813 | - | TGCAGGTA |
| MA0032.1 | MA0032.1.FOXC1 | 5.178817 | 0.871747542 | THBS1 | Homo sapiens | 1990 | 1997 | - | AAGGAGTA |
| MA0032.1 | MA0032.1.FOXC1 | 5.178817 | 0.871747542 | THBS1 | Homo sapiens | 2869 | 2876 | + | TAAGAGTA |
| MA0032.1 | MA0032.1.FOXC1 | 4.974676 | 0.860328425 | THBS1 | Homo sapiens | 746 | 753 | - | ATCCTGTA |
| MA0032.1 | MA0032.1.FOXC1 | 4.9157825 | 0.857034061 | THBS1 | Homo sapiens | 1062 | 1069 | - | CAGCAGTA |
| MA0032.1 | MA0032.1.FOXC1 | 4.822673 | 0.851825743 | THBS1 | Homo sapiens | 464 | 471 | - | GAACTGTA |
| MA0032.1 | MA0032.1.FOXC1 | 4.763779 | 0.848531378 | THBS1 | Homo sapiens | 2292 | 2299 | - | CTTGGGTA |
| MA0032.2 | MA0032.2.FOXC1 | 6.2366185 | 0.847109088 | THBS1 | Homo sapiens | 511 | 521 | - | AAGACCAACAA |
| MA0032.1 | MA0032.1.FOXC1 | 4.7231374 | 0.846257979 | THBS1 | Homo sapiens | 2769 | 2776 | + | CACACGTA |
| MA0032.2 | MA0032.2.FOXC1 | 6.1732674 | 0.846158122 | THBS1 | Homo sapiens | 1558 | 1568 | + | TGTGAAAAGAT |
| MA0032.2 | MA0032.2.FOXC1 | 6.110251 | 0.845212182 | THBS1 | Homo sapiens | 2885 | 2895 | - | AATGAAAACTT |
| MA0032.1 | MA0032.1.FOXC1 | 4.6642437 | 0.842963614 | THBS1 | Homo sapiens | 2710 | 2717 | - | TGGGGGTA |
| MA0032.1 | MA0032.1.FOXC1 | 4.652748 | 0.84232058 | THBS1 | Homo sapiens | 1742 | 1749 | - | TTCCTGTA |
| MA0032.2 | MA0032.2.FOXC1 | 5.7640896 | 0.840015947 | THBS1 | Homo sapiens | 2393 | 2403 | - | TGTCAAAATAA |
| MA0032.2 | MA0032.2.FOXC1 | 5.7433295 | 0.839704317 | THBS1 | Homo sapiens | 611 | 621 | - | AAGGACAACAG |
| MA0032.2 | MA0032.2.FOXC1 | 5.696231 | 0.838997318 | THBS1 | Homo sapiens | 1937 | 1947 | + | CAAGGAAACAA |
| MA0032.2 | MA0032.2.FOXC1 | 5.496076 | 0.835992791 | THBS1 | Homo sapiens | 2245 | 2255 | + | AGTGTCATTAT |
| MA0032.1 | MA0032.1.FOXC1 | 4.500745 | 0.833817898 | THBS1 | Homo sapiens | 402 | 409 | + | CCTTTGTA |
| MA0032.2 | MA0032.2.FOXC1 | 5.3495812 | 0.833793754 | THBS1 | Homo sapiens | 2701 | 2711 | + | TACATAAATTA |
| MA0032.2 | MA0032.2.FOXC1 | 4.971998 | 0.828125847 | THBS1 | Homo sapiens | 1660 | 1670 | + | AATGACAATTT |
| MA0032.2 | MA0032.2.FOXC1 | 4.8330674 | 0.826040354 | THBS1 | Homo sapiens | 1756 | 1766 | + | TCTGTAAACTA |
| MA0032.1 | MA0032.1.FOXC1 | 4.3080997 | 0.823041816 | THBS1 | Homo sapiens | 146 | 153 | + | CACAGGTA |
| MA0032.1 | MA0032.1.FOXC1 | 4.249206 | 0.819747451 | THBS1 | Homo sapiens | 2863 | 2870 | + | GGTTGGTA |
| MA0032.1 | MA0032.1.FOXC1 | 4.2377105 | 0.819104417 | THBS1 | Homo sapiens | 136 | 143 | + | CTCCGGTA |
| MA0032.1 | MA0032.1.FOXC1 | 4.2377105 | 0.819104417 | THBS1 | Homo sapiens | 1543 | 1550 | + | CAGCTGTA |
| MA0032.1 | MA0032.1.FOXC1 | 4.2377105 | 0.819104417 | THBS1 | Homo sapiens | 2164 | 2171 | - | AAGCTGTA |
| MA0032.2 | MA0032.2.FOXC1 | 4.152014 | 0.815817045 | THBS1 | Homo sapiens | 2005 | 2015 | + | TAAGAAAAGAG |
| MA0032.1 | MA0032.1.FOXC1 | 4.178817 | 0.815810052 | THBS1 | Homo sapiens | 264 | 271 | - | CTGGGGTA |
| MA0032.1 | MA0032.1.FOXC1 | 4.178817 | 0.815810052 | THBS1 | Homo sapiens | 2317 | 2324 | + | TCTTTGTA |
| MA0032.2 | MA0032.2.FOXC1 | 4.0736456 | 0.814640658 | THBS1 | Homo sapiens | 2930 | 2940 | + | CTTGACAACAA |
| MA0032.2 | MA0032.2.FOXC1 | 3.9824421 | 0.813271602 | THBS1 | Homo sapiens | 2154 | 2164 | - | AACAGCAACAA |
| MA0032.2 | MA0032.2.FOXC1 | 3.8153298 | 0.810763075 | THBS1 | Homo sapiens | 1519 | 1529 | - | TGCACAAACAG |
| MA0032.2 | MA0032.2.FOXC1 | 3.6044395 | 0.807597396 | THBS1 | Homo sapiens | 2664 | 2674 | + | ACCAAAAACAA |
| MA0032.1 | MA0032.1.FOXC1 | 4.026814 | 0.807307397 | THBS1 | Homo sapiens | 2620 | 2627 | - | GAGGGGTA |
